# Inter-annual variability of the effects of intrinsic and extrinsic drivers affecting West Nile virus vector *Culex pipiens* population dynamics in northeastern Italy

**DOI:** 10.1186/s13071-020-04143-w

**Published:** 2020-05-29

**Authors:** Diletta Fornasiero, Matteo Mazzucato, Marco Barbujani, Fabrizio Montarsi, Gioia Capelli, Paolo Mulatti

**Affiliations:** Istituto Zooprofilattico Sperimentale delle Venezie, Viale dell’Università 10, 35020 Legnaro, Padua Italy

**Keywords:** Mosquitoes, *Culex pipiens*, Population dynamics, West Nile, Italy

## Abstract

**Background:**

Vector-borne infectious diseases (VBDs) represent a major public health concern worldwide. Among VBDs, West Nile virus (WNV) showed an increasingly wider spread in temperate regions of Europe, including Italy. During the last decade, WNV outbreaks have been recurrently reported in mosquitoes, horses, wild birds, and humans, showing great variability in the temporal and spatial distribution pattern. Due to the complexity of the environment–host–vector–pathogen interaction and the incomplete understanding of the epidemiological pattern of the disease, WNV occurrences can be difficult to predict. The analyses of ecological drivers responsible for the earlier WNV reactivation and transmission are pivotal; in particular, variations in the vector population dynamics may represent a key point of the recent success of WNV and, more in general, of the VBDs.

**Methods:**

We investigated the variations of *Culex pipiens* population abundance using environmental, climatic and trapping data obtained over nine years (2010 to 2018) through the WNV entomological surveillance programme implemented in northeastern Italy. An information theoretic approach (IT-AIC_c_) and model-averaging algorithms were implemented to examine the relationship between the seasonal mosquito population growth rates and both intrinsic (e.g. intraspecific competition) and extrinsic (e.g. environmental and climatic variables) predictors, to identify the most significant combinations of variables outlining the *Cx. pipiens* population dynamics.

**Results:**

Population abundance (proxy for intraspecific competition) and length of daylight were the predominant factors regulating the mosquito population dynamics; however, other drivers encompassing environmental and climatic variables also had a significant impact, although sometimes counterintuitive and not univocal. The analyses of the single-year datasets, and the comparison with the results obtained from the overall model (all data available from 2010 to 2018), highlighted remarkable differences in coefficients magnitude, sign and significance. These outcomes indicate that different combinations of factors might have distinctive, and sometimes divergent, effects on mosquito population dynamics.

**Conclusions:**

A more realistic acquaintance of the intrinsic and extrinsic mechanisms of mosquito population fluctuations in relation to continuous changes in environmental and climatic conditions is paramount to properly reinforce VBDs risk-based surveillance activities, to plan targeted density control measures and to implement effective early detection programmes.
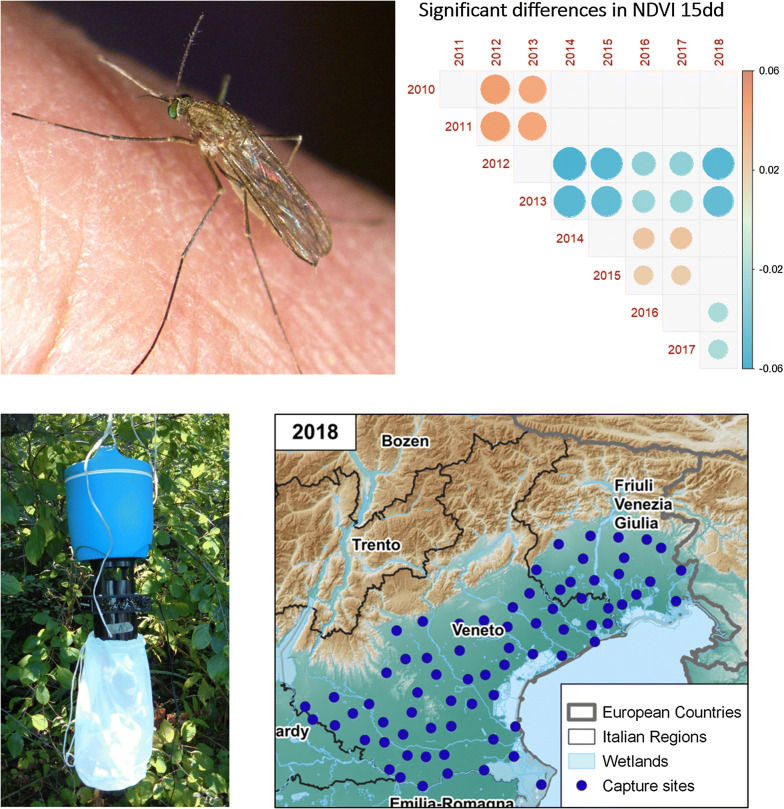

## Background

Climate changes have raised global awareness towards the spread of vector-borne infectious diseases (VBDs) at an international level [1]. VBDs, and arboviruses in particular, represent an important threat for human and animal health, as also stressed by the widespread diffusion of these pathogens in temperate European countries in the last decade [2–5]. Despite recent intensive research, the behavior and the successful expansion of VBDs have not been fully clarified yet. Due to the complexity of the biological cycle of arboviruses, and the limited knowledge of their epidemiological patterns, outbreaks might be temporally and spatially unpredictable [6, 7].

One of the most widely distributed arboviruses in the world is West Nile virus (WNV) [8]. The transmission cycle of WNV includes wild and domestic birds as maintenance hosts, and mosquitoes, primarily belonging to the genus *Culex*, as vectors. Humans and horses, and other mammals to a lesser extent, are susceptible to WNV, although they are considered dead-end hosts [9]. Usually, infections in humans and horses are asymptomatic; however, some infected subjects may develop disease with symptoms ranging from a mild flu syndrome to severe encephalitis or, in the worst cases, death [9].

WNV has been circulating in the Mediterranean Basin at least since the 1960s [10], and numerous outbreaks in human and equine populations have been witnessed since then. In Italy, the first outbreak of WNV was in 1998, in Tuscany, with 14 neuroinvasive cases in horses and no involvement of the human population [11]. The virus re-appeared in 2008, with 251 outbreaks confirmed in equines located in three different regions in northeast Italy [12]. Since then, WNV has been recurrently detected in horses, wild birds and humans. In northeast Italy, WNV was also detected in pools of *Culex pipiens* mosquito in 2010, and positive pools were thereafter found every year. Nowadays, WNV is considered endemic in the northeastern Italy [13], and *Cx. pipiens* it is deemed as its primary vector [14].

The first national veterinary surveillance system for WNV was implemented in 2001, and was further improved in 2010, after the re-emergence of the virus in north-east Italy. In 2013 a risk-based surveillance approach was attempted by the regional Veterinary Authorities in Veneto and Friuli Venezia Giulia (FVG), and based on the identification of recurrent WNV hotspots in humans, equines and mosquitoes [15]. The plan was annually updated with the areas considered most at risk of WNV reactivation, according to the continuous evolution of the epidemiological situation.

In the last decade, the timing and location of positive cases detected in Veneto and FVG varied substantially. In fact, the distribution of the outbreaks over the years did not seem to follow a predictable pattern, except for a progressively earlier detection of the first positive cases in mosquitoes and horses, and a massive increase in human cases, which peaked in 2018 with 257 confirmed cases [16]. The hypothesis underlying this trend over the years could be related to changes in several drivers of the disease, including the ecological and biological aspects of the vectors, virus ecology, reservoir hosts, and the human population [17–19]. There is a strong need for in-depth studies on the ecology of the environment-host-vector-pathogen interaction, in order to seek which factors could trigger earlier WNV reactivation. In particular, variations in vector population dynamics may represent a key point of the recent success of WNV and, more in general, of the VBDs [20].

It is widely acknowledged that vector population abundance is strongly affected by environmental and climatic variability [18], as well as by intrinsic, density-dependent variables [21, 22], even though no clear patterns of these interactions have emerged from several studies [23]. A thorough knowledge of the ecology of the vector population is the first step to analyse the complex phenomenon of the VBDs in their entirety.

Here, we investigated the variations of *Cx. pipiens* population abundance using environmental, climatic and trapping data obtained over nine years from WNV entomological surveillance programme implemented in northeastern Italy. The seasonal mosquito population growth rates were analyzed to evaluate the presence of possible differences among single-year and overall period (i.e. all data available from 2010 to 2018) data analyses, in order to identify the most significant combinations of variables outlining the *Cx. pipiens* population dynamics. The great variability in the pattern of occurrence of WNV cases in this territory led us to envisage a potential different response for vector population dynamics to different combinations of environmental conditions; this could affect the distribution pattern of the disease distinctively over the years. Ultimately, a more realistic acquaintance of vector population drivers could offer baseline information for planning targeted density control measures, improved risk-based surveillance activities and early detection programmes.

## Methods

### Area and period of study

**Fig. 1:**
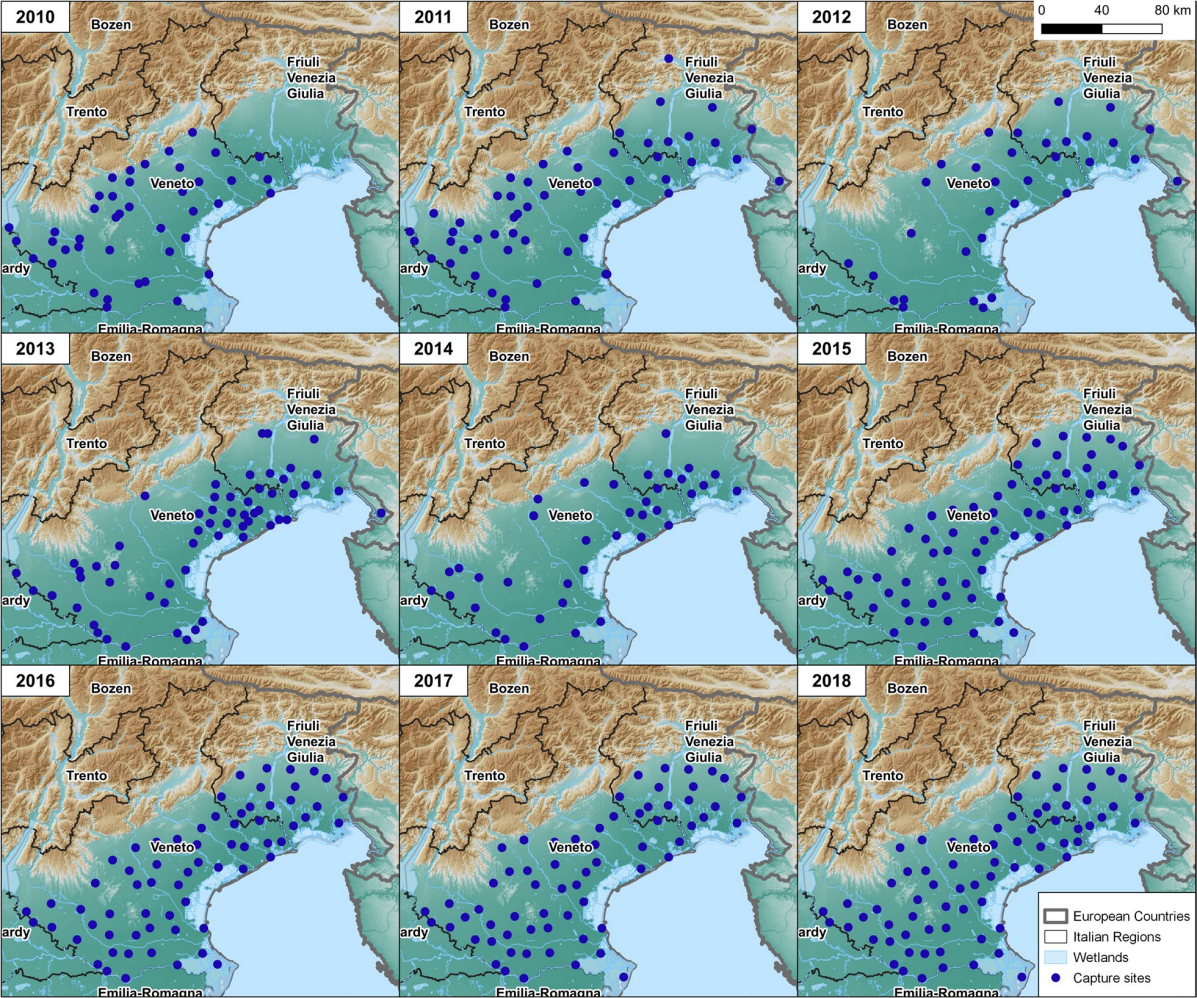
Geographical location of capture sites during the years of study (2010–2018)

The study was conducted exploiting the results of entomological surveillance plans carried out in the flatlands of Veneto (below 300 m above the sea level) and FVG Regions, northeastern Italy. Environmental, climatic, and entomological data were collected for the period 2010–2018 for Veneto, and for 2011–2018 for FVG, as entomological surveillance started later in this Region (Fig. 1).

### Mosquito data collection

Mosquito captures were conducted using CDC traps baited with dry ice pellets as source of carbon dioxide (IMT^®^, Italian Mosquito Trap, Cantù, Italy), aiming at attracting blood meal seeking females and powered by a 12 V battery. Traps were activated overnight every two weeks, from 17:00 h to 11:00 h on following day. The entomological surveillance season ran each year between approximately the first week of May until two consecutive captures with no mosquitoes collected (generally between the last week of October and the first week of November).

CDC traps were located mainly in rural/agricultural areas, but some natural and urban sites were also included (Additional file 1: Table S1). Collected adult mosquitoes were transported at 4 °C to the laboratory where they were stored at − 20 °C, and then identified under a stereomicroscope using taxonomic keys [24].

Only adult females belonging to the *Cx. pipiens* complex (i.e. encompassing both the *Cx. pipiens pipiens* and *Cx. pipiens molestus* forms, which are phenotypically indistinguishable) have been included in the analyses, as they are considered the most important WNV vectors in Italy [14]. To obtain a more reliable picture of the population dynamics, only traps that had at least four consecutive catches were included in the study.

### Environmental data

Environmental data were extracted through a system developed at the Istituto Zooprofilattico Sperimentale delle Venezie and named Environmental data for Veterinary Epidemiology (EVE), and included: temperature and vegetation measurements derived from NASA MODIS satellite imagery [25, 26], precipitation derived from interpolation of ground sensors (ARPA Veneto, https://www.arpa.veneto.it; ARPA FVG, https://www.osmer.fvg.it; Autonomous Province of Trento, https://www.meteotrentino.it; Autonomous Province Bozen, http://meteo.provincia.bz.it), and length of daylight (Table 1). All the environmental data were stored and managed as geographical raster images, with a spatial resolution of 1 km for precipitation and vegetation measurements, and 200 m for temperature.

**Table 1:** Extrinsic and intrinsic variables considered in the analysis, and variables included in the IT-AIC_c_ models

Variable	Description	Included in IT-AIC_c_ approach^a^
GMP	Density dependent variables measuring the saturation of the carrying capacity	Yes
DT.h	Length of daylight (hours)	Yes
DMI_15d_	De Martonne aridity index in 15 days prior to capture (mm/°C)	Yes
GDD_15d_	Growing degree days in 15 days prior to capture	Yes
NDVI_15d_	Maximum normalized difference vegetation index in 15 days prior to capture	Yes
PREC	Daily cumulative precipitation (mm)	Yes
PREC.avg_15d_	Average cumulative precipitation in 15 days prior to capture (mm)	No
PREC.k_15d_	Precipitation kurtosis in 15 days prior to capture (mm)	Yes
PREC.sd_15d_	Precipitation standard deviations in 15 days prior to capture (mm)	No
T.avg_15d_	Average temperature in 15 days prior to capture (°C)	No
T.k_15d_	Temperature kurtosis in 15 days prior to capture (°C)	Yes
T.sd_15d_	Temperature standard deviations in 15 days prior to capture (°C)	Yes
T.night	Average temperature recorded during the overnight capture period (18:00–6:00 h) (°C)	Yes

^a^Some variables were excluded due to collinearity

As *Cx. pipiens* larvae were assumed to develop into adults in approximately two weeks [27, 28], we extracted measures concurrent with the capture moments, and summarizing values for the 15 days prior to trapping. This allowed describing the potential influence of environmental and climatic factors on both adult mosquito dynamics and the development of the larval forms. Synoptic variables included: the average of the distributions of the cumulative daily precipitation (PREC.avg_15d_) and of the daily average temperature (T.avg_15d_); the De Martonne Index (DMI_15d_); the normalised difference vegetation index (NDVI_15d_); and the growing degree days (GDD_15d_). Furthermore, since mosquitoes had been observed being sensitive to fluctuations in weather trends [29], measures of climatic variability were also included in the analyses to better investigate associations between mosquito dynamics and their drivers. In addition to average measurements, the kurtosis and standard deviation of the distributions of cumulative daily precipitation (PREC.k_15d_ and PREC.sd_15d_), and of the daily average temperature (T.k_15d_ and T.sd_15d_) in the 15 days prior captures were also taken into account, to evaluate whether the extent of climatic variation may influence the mosquito population growth rates, during the assumed larval development period.

The De Martonne index provides information on the overall level of aridity of an area [30]. Values lower than 20 are considered indicative of arid or semi-arid lands. The formula proposed for monthly estimates [31] was adapted to account for only 15 days prior to capture as follows:

$$ DMI_{15d} = \frac{{24.3*PREC_{15d} }}{{T.avg_{15d} + 10}} $$

where *PREC*_*15d*_ and *T.avg*_*15d*_ are the cumulative precipitation and the average temperature during 15 days, respectively, and 24.3 is a scaling factor to account for calculation of DMI at a 15-day scale.

As NDVI showed negligible variation in time, in each single capture season, only the maximum values observed within 1 km from the capture sites in the 15 days prior to the trappings were included in further analyses.

The growing degree days (GDD) were considered to capture the direct effect of temperature on development cycle of mosquitoes. GDD estimates were considering a defined range for mosquito activity [23, 32]. A lower threshold (LT) was set at 11.5 °C and an upper threshold (UT) was set at 34.7 °C, accounting for corrections for average differences between air temperature and the land surface temperature (LST). GDD daily values were calculated as:

$$ \left\{ {\begin{array}{ll} 0 &  \quad {if\;T < LT} \\  T - LT & \quad  if\;T \ge LT\;and\;T \le UT\\ UT & \quad if\;T > UT  \end{array} } \right. $$

where *T* is mean daily LST.

Four daily values of LST were available for each trapping day, recorded at 13:30 h and 22:30 h on the day in which the trap was positioned, and at 1:30 h and 10:30 h on the following day. The four measurements were combined to obtain an estimate of the average temperature during the period of most intense *Cx. pipiens* activity, which was observed occurring between 18:00 h and 6:00 h in this area (T.night) [33, 34].

To capture the effect of diapause and the suppression of feeding behaviour on mosquito populations [35, 36], the length of daylight was included in the analyses (DT.h). In fact, the start and end of diapause had been described as strongly related to photoperiod, indicating that *Cx. pipiens* mosquitoes could enter a quiescent period when the length of daylight decreases [35]. The length of daylight was retrieved from the repositories of the Astronomical Applications Department of the USA Naval Observatory (http://aa.usno.navy.mil/).

### Statistical analyses

To investigate the trends of the mosquito population abundance over the years, growth rates were calculated at each capture site. Mosquito abundances were processed to compute the geometric moving average on three consecutive captures, allowing to flatten short term fluctuations and highlight longer term trends. The per-capita growth rate was then computed as:

$$ r_{t} = log\frac{{N_{t + 1} }}{{N_{t} }} $$

where *N*_*t*_ is the averaged population abundance at time *t* and *N*_*t+1*_ is the averaged population abundance at the succeeding capture.

A series of statistical models was fitted using Maximum Likelihood mixed-effects linear regression (linear mixed-effects model, LME) [37], to evaluate the influence of the environmental and climatic drivers on the mosquito population growth rate trends. An additional density-dependent variable was included in the analysis, considering that the population growth rates could follow a Gompertz-logistic model, which accounts for the saturation of the carrying capacity at each capture site [23, 38]. According to the Gompertz model, the density dependent variable (indicated as GMP) was calculated as:

$$ GMP = \frac{\log \left( N \right)}{\log \left( K \right)} $$

where *N* is the abundance of mosquitoes per capture and *K* is the carrying capacity, which was assumed to vary through years and capture sites and was set as the most abundant capture recorded for each year and site.

Data were grouped per capture site and year, as nested random effects, as we assumed that population growth rates might vary among different capture sites, and for each site, the population growth rates might vary in different years. Before model fitting, all variables included in the statistical analyses were scaled and centered to allow easier inferences through the direct comparison of the coefficients estimates: higher absolute values of the estimates indicate stronger effects on the population growth rates [39]. A temporal autocorrelation structure was also considered, to handle repeated measurements and seasonal data, allowing to account for the presence of potential serial correlation of the residuals. A series of statistical models were then built to investigate the effects of intrinsic and extrinsic drivers on the mosquito population dynamics. In particular, one model was fit including all data collected during the whole study period (2010–2018), while nine other models were fit on single-year data, separately. These full initial models were built including the GMP variable, and the environmental and climatic variables (Table 1). Further sets of models were then generated considering all of the possible combinations of variables.

Model selection was based on the information theoretic (IT) approach [40] based on the corrected Akaike information criterion (IT-AIC_c_) [40–42]. The IT-AIC_c_ approach is based on a comparative fit analysis through the calculation of Akaike weights (wAIC_c_) for each generated model and can be interpreted as the probability of each single model to be the AIC-best model [42]. Following the IT-AIC_c_ approach, models within each set were compared to assess whether there was a single model with high statistical support or many models with a similar fit. In the absence of a single outperforming model, model averaging was performed, combining the parameter estimates from the selected set of models considering the contribution of each model being proportional to its likelihood weight [40, 42]. For each generated set of models, a subset was selected so that their total cumulative wAIC_c_ was equal to 0.95. This subset of models was assumed to include the AIC_c_-best model, with a probability of 0.95. Furthermore, the number of selected models provides information on the level of uncertainty encountered. From models with averaged coefficients, the importance of each single predictor variable was calculated as the sum of the AIC_c_ weights over all of the models in which the variable of interest appeared, and was intended as the probability of each single predictor to appear in the AICc-best model [42].

The linear mixed-effect models were fitted using the *nlme* package [43], the IT-AIC_c_ approach was performed through the package *MuMIn* [44], using R statistical software version 3.5.2 [45].

## Results

### Annual trends in mosquito catches and abundance, and in environmental and climatic factors

A variable number of trap sites were operating in the study area each year, according to variations in the regional surveillance plans (Table 2).

**Table 2:** Number of mosquitoes collected during the sampling period 2010–2018

Year	2010^a^	2011	2012	2013	2014	2015	2016	2017	2018
Trapping period^b^	17 May–26 Oct	17 May–25 Oct	22 May–31 Oct	14 May–29 Oct	19 May–29 Oct	3 Jun–28 Oct	25 May–27 Oct	5 Jun–30 Oct	4 Jun–4 Oct
No. of active traps	42	55	33	60	38	65	66	65	72
Total no. of captures	423	552	310	732	395	611	611	667	577
Total no. of collected mosquitoes	119,742	69,080	88,210	275,450	88,580	92,194	188,570	101,424	104,069
Total no. of collected *Cx. pipiens*	110,892	59,573	70,182	257,970	75,567	79,733	172,759	82,950	87,197
Mean no. of *Cx. pipiens* per capture (95% CI)	262.15 (208.17–316.14)	107.92 (91.53–124.31)	226.39 (186.21–266.58)	352.42 (308.75–396.09)	191.31 (165.62–217.00)	130.50 (112.12–148.87)	246.45 (219.58–273.31)	137.33 (118.35–156.31)	150.34 (130.85–169.83)
Percentage of *Cx. pipiens*	92.61	86.24	79.56	93.65	85.31	86.48	91.62	81.79	83.79
Total no. of identified species	15	16	17	21	17	18	15	19	16

^a^In Veneto Region only

^b^Date of first capture - date of last capture

The mean abundance of captured mosquitoes varied significantly between several years (*F*_(8, 4899)_ = 28.55, *P* < 0.001; Additional file 1: Figure S1). Overall mosquito captures were most abundant in 2013 (*P* < 0.05 for all of the comparisons), followed by 2010. Conversely, 2011 in particular, but also 2015, 2017, and 2018 were the years with least abundant mosquito populations. Despite several statistical differences observed in the population consistency, the mosquito population growth rates did not vary significantly among years (*F*_(8, 4899)_ = 0.70, *P* = 0.697). Statistical differences were observed for the density-dependent variable (*F*_(8, 4899)_ = 4.83, *P* < 0.001); *post-hoc* tests showed an overall higher value for 2018 (compared with 2010, 2013 and 2015–2017), and lower estimates for 2017 when compared with 2011, 2014, and 2018 (Additional file 1: Figure S1).

The cumulative precipitation measured on the days of capture resulted significantly variable throughout the whole period of study (*F*_(8, 4899)_ = 12.70, *P* < 0.001), with *post-hoc* Tukey’s test indicating similar higher values for 2014 and 2015 (Additional file 1: Figure S1). Significant between-year differences were also detected for cumulative precipitations on 15 days prior to trappings (*F*_(8, 4899)_ = 17.90, *P* < 0.001), and the higher values were recorded in 2010 and 2014 (Additional file 1: Figure S1). Both temperatures concurrent with the trapping nights and average temperatures recorded 15 days before captures showed significant differences (*F*_(8, 4899)_ = 27.73, *P* < 0.001 and *F*_(8, 4899)_ = 43.16, *P* < 0.001, respectively). Overall, night temperatures concurrent with mosquito captures, and average temperatures in 15 days before trapping were markedly lower for 2010 with respect to other years, and for 2014 in comparison with 2015–2018. In contrast, 2018 showed the warmest temperatures in the whole period of study (on average, + 1.70 °C on the concurrent night and + 2.08 °C in the 15 days before capture, compared to the other years; Additional file 1: Figure S1). Also, the level of aridity significantly varied by year (*F*_(8, 4899)_ = 17.97, *P* < 0.001). *Post-hoc* tests indicated a similar trend with the average precipitation in 15 days: 2010 and 2014 showed an overall higher DMI_15d_, indicating lower aridity levels (Additional file 1: Figure S1). As for NDVI_15d_ (*F*_(8, 4899)_ = 18.90, *P* < 0.001), significantly lower values were observed in 2012 and 2013, while more abundant vegetation indices were reported for 2014, 2015, and 2018 (Additional file 1: Figure S1).

### Drivers of mosquito population growth rates

Preliminary analyses indicated the presence of a high correlation between some environmental variables (Additional file 1: Table S2). Therefore, before model fitting, the average values of precipitation and temperature were excluded, due to a high correlation with PREC.sd_15d_ and DMI_15d_, and with GDD_15d_, respectively. Variance inflation factors (VIFs) were calculated on a preliminary model fitted with the remaining variables, resulting in high VIF for DMI_15d_ and PREC.sd_15d_ (9.24 and 7.72, respectively), indicating potential collinearity. Following further exploratory GLMM considering either DMI_15d_ or PREC.sd_15d_ alone as independent variables, the model accounting for DMI_15d_ as a predictor of population growth rates showed greater statistical support (wAIC_c_ = 0.68). Therefore, we excluded PREC.sd_15d_ from further analyses. All of the initial models were then built including ten explanatory variables; GMP, DT.h, PREC, PREC.k_15d_, T.k_15d_, T.sd_15d_, T.night, DMI_15d_, GDD_15d_ and NDVI_15d_. The model selection process based on the IT-approach generated 1024 models for each year and for the whole period (2010–2018), encompassing all of the possible combinations of explanatory variables. For each set, different numbers of models were necessary to reach a total cumulative wAIC_c_ of at least 0.95 (Table 3). The subsets ranged from a minimum of 70 models for 2015 to a maximum of 195 models for 2017, indicating different levels of uncertainty among years. Additional file 2: Table S3 reports the subsets of selected models; the relative likelihood-ratio based pseudo-*R*^2^ ($R^{2}{_{\text{LR}} }$) were calculated to express the proportion of data variation explained by the models. $R^{2}{_{\text{LR}} }$ varied from a minimum of 0.70 in 2011 to a maximum of 0.82 in 2012, indicating and overall high goodness-of-fit.

**Table 3:** Number of selected models for each subset, with cumulative wAIC_c_ = 0.95

2010–2018	2010	2011	2012	2013	2014	2015	2016	2017	2018
84	106	152	129	79	104	70	92	195	107

**Fig. 2:**
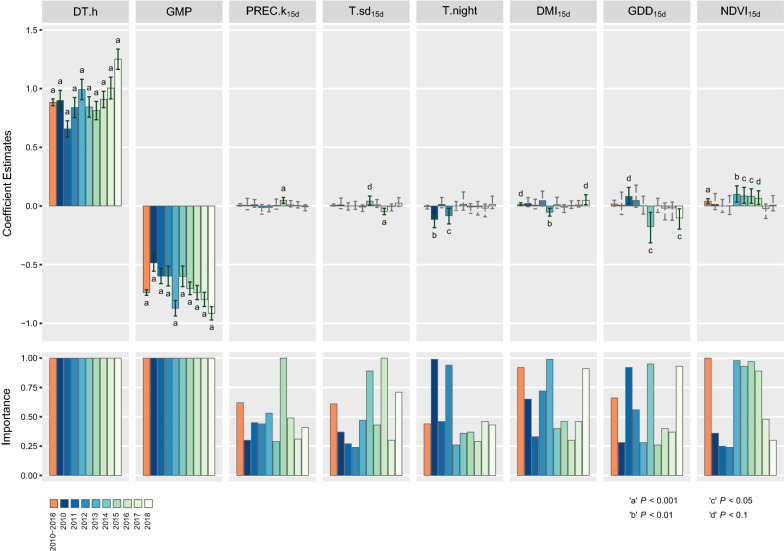
Graphical representation of the results of the average models per year; upper row: weighted averages of coefficients, 95% CI are shown (solid lines, significant coefficients; dashed lines, non-significant coefficients); lower row: importance of the variables. Only significant variables for at least one year of study (*P* < 0.1) are reported

The results of the analyses (i.e. coefficient estimates, confidence intervals, *P*-values and importance) for the significant variables are graphically shown in Fig. 2; Additional file 3: Table S4 reports the numerical coefficients estimates, confidence intervals and importance for all the variables included in the averaged models. Additional file 1: Figure S2 shows annual trends for temperature, aridity and vegetation indexes to improve the interpretation of the results of our study. The highest absolute values for the coefficients were associated with length of daylight (DT.h) and with the density-dependent variable (GMP) (Fig. 2, Additional file 3: Table S4). Coefficients of daylight were always positive and ranged between 0.657–1.251, suggesting that higher population growth rates were observed when days were longer. The density-dependent variable always had a negative sign, which reflects its inverse relationship with the population growth rates (i.e. the growth rates decreased when the carrying capacity was saturating). Both DT.h and GMP were always highly significant (*P* < 0.001) and had maximum importance (i.e. importance = 1), which indicated they were included in all of the models selected for the averaging process. Precipitation values concurrent with mosquito trapping (PREC) were not significant in any set of models (Additional file 3: Table S4). As factors measured on the day of capturing did not provide indications on the effects on population growth rates, but more on how the adult mosquitoes were affected, the results suggested rainfall did not likely influence the capture of adult mosquitoes. As for the variables summarizing the variation of rainfall during larval development, the kurtosis of the distribution of cumulative precipitation values (PREC.k_15d_) was significant only for 2015 (*P* < 0.001), although the coefficient was not as large as those observed for DT.h and GMP (Coef. = 0.048) (Fig. 2, Additional file 3: Table S4). The positive coefficient suggested that increases in population growth rates were more likely when the cumulative rainfall followed a leptokurtic distribution, which indicated that periods characterized by a more homogeneous rainfall may favor larval development and consequently the population growth rate. The coefficient for the GDD_15d_ was significant and highly important in 2014 and 2018 (*P* < 0.05, importance = 0.92 and 0.95, respectively), with negative coefficients (2014, Coef. = − 0.175; 2018, Coef. = − 0.102) (Fig. 2, Additional file 3: Table S4). Conversely, the coefficient for 2011 (*P* < 0.1) was positive, although with a relatively smaller effect on the population growth rate (Coef. = 0.081). Increased temperature variability during larval development (T.sd_15d_) appeared to be positively correlated to mosquito population dynamics in 2014 (Coef. = 0.042, *P* < 0.1), while an opposite effect was observed for 2016 (Coef. = − 0.047, *P* < 0.001) (Fig. 2, Additional file 3: Table S4). Overall temperatures in 2014 were, on average, significantly lower (Additional file 1: Figures S1, S2), and characterized by the smallest average of standard deviations, which corresponds to a smaller range of temperatures. This means that 2014 was characterized by a cooler summer and a less harsh winter. The results of our analyses suggested that even small rises in temperature during an on average cooler period may lead to increased population growth rates, as they probably promote the development of larval forms. On the contrary, in 2016 temperatures did not present peculiar trends compared to the other years, although temperatures seem to decrease less steeply after the July peak (Additional file 1: Figure S2). The slower decrease of summer temperatures may have determined a prolonged period characterized by more homogeneous temperatures, which may have been more favorable for the mosquito population growth. The average temperature during the captures (T.night) had a significant contribution on population growth rates for 2010 and 2012 only, with a negative effect (Coef. = − 0.114 and –0.082, *P* < 0.01 and *P* < 0.05 for 2010 and 2012, respectively; importance > 0.90 for both years) (Fig. 2, Additional file 3: Table S4). No other variables related to temperature appeared to have a significant influence on mosquito population dynamics (Additional file 3: Table S4). The coefficients for the De Martonne index (DMI_15d_) was significant for the averaged model built for 2013 (Coef. = − 0.052, *P* < 0.01, importance = 0.99), and for 2018 (Coef. = 0.049, *P* < 0.1, importance = 0.91) (Fig. 2, Additional file 3: Table S4). A smaller effect was also observed for the whole period of study (Coef. = 0.014, *P* < 0.1, importance = 0.92). A positive coefficient of 0.040 for the NDVI_15d_ was highly significant (*P* < 0.001) and important (importance = 1.00) in the averaged model built for the entire study period (2010–2018) (Fig. 2, Additional file 3: Table S4). As for single-year averaged models, the coefficient estimates showed high importance (importance > 0.80) only for years 2013–2016 and ranged between 0.064 (2016) and 0.100 (2013).

## Discussion

In-depth knowledge of mosquito population dynamics and variability over time is paramount to understand how WNV infections might spread in an area. Although *Cx. pipiens* population dynamics is known to be regulated by both density-dependent and climatic and environmental factors [23, 46, 47], the complexity of its ecology hampers the capacity to assess unequivocally to what extent the key drivers regulate the population dynamics. In this study, we aimed at evaluating whether the effect of environmental, climatic, and density-dependent factors affected the population dynamics in a consistent way, year by year, or if there were variations on how vector populations responded along the nine years of the study period.

From our results, it emerged that only two factors were regularly detected in the models, indicating consistency in how they are associated with *Cx. pipiens* population dynamics: the first, population density, appeared to always have a negative effect, while the second, length of daylight, had a positive influence on the population growth rate. The negative sign associated with the density-dependent variable reflects the trend of the growth rate saturation curve as described by a Gompertz-logistic model [38]: the growth rates decrease as the mosquito density reaches the population carrying capacity. The impact of population density stresses the relevance of endogenous factors in regulating the internal dynamics of the *Cx. pipiens* population [47, 48], however, they may not be completely sufficient to entirely explain the variation observed.

The length of daylight had the strongest effect, with positive coefficients, indicating that growth rates were higher in periods with a greater number of light hours, and dropped when the photoperiod decreased. This could be related to changes in the mosquito behavior [48, 49], as females prepare for the diapause and stop searching for blood meals, opting for a more sugary diet [50].

The effect of precipitation was appreciable only for one model out of ten, stressing the uncertainty already reported in literature on the effect of rainfall on mosquito populations [48, 51]. In general, it is acknowledged that precipitation could contribute to create small basins of enriched water, suitable as reproductive foci for mosquitoes [29]. Also, since larval stages are water-dependent, precipitation could play an important role in creating and maintaining the wet environment necessary for the development of mosquitoes [52]. On the contrary, an excess of rainfall flushes the development foci used by larvae, leading to a decrease in the number of adult mosquitoes at a later moment, determining a reduction in the population growth rates [29, 53].

Of the two factors we included in the model to account for the variability of temperature during the larval development period, only the standard deviation appeared to be significantly associated with variations in mosquito growth rates, although its effect was not univocal. This was also observed in other studies, which inferred that environmental drivers such as temperature might have complex and opposing impacts on the demographic rates of mosquito life-cycle stages [54]. Overall, increasing temperatures lead to a faster development of the immature stages and stimulate the flight activity of *Cx. pipiens*, but too much heat could dry up the eggs and larvae, as well as decrease the lifespan of adults. As for the growing degree days, within certain limits, higher GDD_15d_ appeared to significantly determine a greater growth rate, as the accumulated heat allowed eggs and larvae to develop into adults, so a larger number of adult mosquitoes can be trapped in the succeeding capture. However, when the amount of GDD_15d_ is very high, it could also lead to an apparent decrease in growth rates. In fact, the generally positive effect of temperature could shorten the time needed for larvae to develop into the adult form [36]. Therefore, multiple overlapping mosquito generations might have developed in 15 days. The increase in population abundance would then cause a reduction of growth rates due to the density-dependent effect, as the carrying capacity is rapidly saturated.

The effect of average overnight temperature appeared to be in contrast with what has been reported in the literature, namely that temperatures are positively linked to mosquito abundance [48, 51, 55]. Our observation suggested that night temperatures alone might not be sufficient to account for the variations observed in the trapped adult mosquitoes, and likely unaccounted interactions with other environmental/ecological factors could be responsible for the negative effect found. A definitive explanation for this relationship remains elusive, and further studies are needed to investigate this apparent discrepancy in the nature of temperature effects.

Contrasting effects were also detected for the De Martonne index, which described the effect of the arid environment. Overall, it appeared that a more humid environment is positively associated with increased growth rates, although the finding was significant only for the overall period model and for 2018. The negative impact of DMI_15d_ on mosquito population dynamics for 2013, although counterintuitive, could be partially explained when looking at the precipitation and temperature trend of that year. In fact, 2013 was characterized by a warmer and rainy spring followed by an exceptionally dry and arid summer. Heavy rainfall in spring might have contributed in creating suitable ecological niches for the development of larval stages, which in turn could have led to an increase in the abundance of adult mosquitoes at the beginning of the entomological surveillance season. This might have elicited a faster saturation of the population carrying capacity, with a consequential decrease of the population growth rates due to density-dependent effects. Another potential explanation could be related to the fact that drought conditions can cause massive evaporation, consequently determining the enrichment of the existing larval foci with organic material, hence creating environments more suitable for larval development [53, 56]. In addition, in periods of drought, artificial irrigation is more frequent especially for wheat and corn fields, which are particularly abundant in north-eastern Italy. The continuous availability of water provides suitable *Cx. pipiens* larval foci, and in turn higher growth rates and WNV diffusion [57].

The results for the measurements of biomass (NDVI_15d_) were consistent with other studies, where similar effects were observed [29, 51]. High estimates of NDVI in rural/agricultural areas had been put into the relationship with the presence of breeding and resting sites suitable for *Cx. pipiens* mosquitoes, also providing a source of organic matter and nutrients necessary for the developing of the immature stages [58].

Overall, the results of the models highlighted that population density and daylight were the predominant factors regulating mosquito population dynamics, as they appeared in all of the models with the highest coefficients and direct effects. Other drivers encompassing environmental and climatic variables also had an impact, although sometimes counterintuitive and not univocal. Analysing single-year datasets and comparing the results to overall period models, highlighted remarkable differences in coefficients magnitude, sign, and significance. In fact, it could be argued that mosquito populations may respond in different ways to fluctuations of environmental conditions, according to different variables combinations over the years. The inclusion of factors indicating environmental variability was useful to investigate association patterns between mosquito dynamics and climatic trends, as also seen in other countries [29], where population densities appeared to be positively correlated to increased variability in both temperature and rainfall. However, in our study this result occurred only for years in which climatic factors appeared to be extreme, and thus unfavorable for *Cx. pipiens* population growth. Therefore, when environmental conditions are more suitable for mosquito populations, a more homogeneous trend was positively associated with increased population growth rates.

The sets of models selected per year, with a cumulative wAIC_c_ of at least 0.95, were always very numerous, ranging from 70 models in 2015 to 152 for 2011. This output of the IT-AIC_c_ approach reflected the high level of uncertainty in defining single best models for each year of analysis. However, to limit the complexity of the initial models, and hence the potential uncertainty in results, interactions between variables were not taken into account in the models, as well as quadratic or non-linear effects for variables as temperature and temperature-derived factors, which could actually have a non-linear influence on mosquito population dynamics. Further study would be required to investigate how to incorporate more complex variables to model the population dynamics.

An acknowledged limitation of our study was related to the source of mosquito data, which derived from the regional WNV entomological surveillance plan. The surveillance activities had a precise start date, which likely did not catch the exact beginning of the mosquito season after the end of the winter diapause. This could hamper the statistical support of the models, as it was not possible to assess how the intrinsic and extrinsic drivers influenced the population dynamics in the earliest phases. The availability of mosquito collection data for the entire non-diapause period of *Cx. pipiens* could improve the robustness of the models, and soundness of inferences made upon them. Another aspect to be recognized is related to the different number of capture sites in the nine years of study and their variable position throughout the study period, in accordance with the needs and requirements of the regional WNV surveillance plan. In fact, inconsistencies in the trapping patterns might introduce artefacts and biases, indicating variations that may not necessarily reflect the actual fluctuations in local population dynamics during the study period [29]. To overcome this issue and limit as much as possible any loss in performance, we considered environmental and climatic factors measured at each capture site during the trappings and the two weeks before. This would help to capture the actual influence of the location on the mosquito population dynamics.

## Conclusions

This study provides an extensive insight into the *Cx. pipiens* population dynamics in north-eastern Italy, an area considered endemic for WNV. The availability of a large dataset with entomological, environmental, and climatic data over a nine-year period, allowed the estimation of the contribution of the most important drivers on the regulation of the population dynamics, highlighting interesting ecological differences among years. One of the most interesting results indicates that an overall model for the entire study period might be excessively generic, not allowing to assess the granularity of the effects of environment and climate on mosquito populations along the entire study period. In fact, differences were detected in environmental/climatic drivers in single-year models, allowing inference that different combinations of factors or different trends of factors might have distinctive, and sometimes divergent, influences on mosquito dynamics. Enhancing the understanding of the intrinsic and extrinsic mechanisms of mosquito population fluctuations in relation to continuous changes in environmental conditions is paramount to properly reinforce risk-based surveillance plans and control measures. The capacity to identify areas where an increased mosquito population density should be expected would allow triggering targeted countermeasures such as disinfestations, as well as suggesting where to capture mosquitoes to optimize resources for entomological WNV surveillance.

## Supplementary information

**Additional file 1: Table S1.** Classification of the mosquito trapping sites per year, based on the land cover classes (level 2 of the Corine Land Cover 2018) where the traps were located. **Figure S1.***Post-hoc* Tukey’s comparisons between years for average mosquito abundance, density-dependent variable and environmental variables. **Figure S2.** Annual trends of the level of aridity (DMI), normalized difference vegetation index (NDVI) and temperature (maximum, red line; minimum, blue line, LST) from 2010 to 2018. Gray lines: monthly averages for the whole study period. **Table S2.** Correlation matrix of intrinsic and extrinsic variables included in the analyses (absolute correlation values > 0.65 are reported in boldface).

**Additional file 2: Table S3.** Lists of models per year included in the subsets considered in the study; models are ordered by decreasing wAIC_c_ and likelihood-ratio-based pseudo-*R*^2^ ($R^{2}{_{\text{LR}} }$) is reported to indicate the goodness-of-fit of each model.

**Additional file 3: Table S4.** Coefficients estimates (Est.), 95% confidence intervals (CI), significance and importance (Imp.) of the overall period (2010–2018) and single-year models with averaged parameters.

## Data Availability

Data supporting the conclusions of this article are included within the article and its additional files. The datasets used and/or analyzed during the present study are available from the corresponding author upon reasonable request.

## Abbreviations

CI: confidence interval
DMI: De Martonne index
GDD: growing degree days
IT-AIC_c_: information theoretic methodologies based on the corrected Akaike information criterion
LME: linear mixed-effects model
LST: land surface temperatures
NDVI: normalized difference vegetation index
VBDs: vector borne diseases
wAIC_c_: Akaike weights
WNV: West Nile virus
