# Supplementary material for: Inter-annual variability of the effects of intrinsic and extrinsic drivers affecting West Nile virus vector Culex pipiens population dynamics in northeastern Italy
Source: Parasit Vectors. 2020 May 29;13:271. doi: 10.1186/s13071-020-04143-w (PMC7260749; doi:10.1186/s13071-020-04143-w)
Supplement: Supplementary file 1 — Additional file 1: Table S1. Classification of the mosquito trapping sites per year, based on the land cover classes (level 2 of the Corine Land Cover 2018) where the traps were located. Figure S1.Post-hoc Tukey’s comparisons between years for average mosquito abundance, density-dependent variable and environmental variables. Figure S2. Annual trends of the level of aridity (DMI), normalized difference vegetation index (NDVI) and temperature (maximum, red line; minimum, blue line, LST) from 2010 to 2018. Gray lines: monthly averages for the whole study period. Table S2. Correlation matrix of intrinsic and extrinsic variables included in the analyses (absolute correlation values > 0.65 are reported in boldface). [file 13071_2020_4143_MOESM1_ESM.docx]

**Additional file 1**

**Table S1.** Classification of the mosquito trapping sites per year, based on the land cover classes (level 2 of the Corine Land Cover 2018) where the traps were located

| **CLC Classes** | **2010** | **2011** | **2012** | **2013** | **2014** | **2015** | **2016** | **2017** | **2018** |
| --- | --- | --- | --- | --- | --- | --- | --- | --- | --- |
| Arable land | 22 | 25 | 16 | 28 | 23 | 36 | 38 | 33 | 37 |
| Artificial non-agricultural vegetated areas | 2 | 1 | - | - | - | - | - | - | - |
| Forests | 1 | 2 | 1 | 2 | - | - | - | - | - |
| Heterogeneous agricultural areas | 9 | 11 | 6 | 12 | 4 | 8 | 7 | 9 | 10 |
| Industrial commercial and transport units | 1 | 1 | 1 | 2 | 3 | 3 | 3 | 2 | 3 |
| Inland waters | 1 | 1 | - | - | - | - | - | - | - |
| Marine waters* | 1 | 1 | 1 | 1 | 1 | 1 | 1 | 1 | 1 |
| Pastures | - | 2 | - | - | - | - | - | - | - |
| Permanent crops | 1 | 2 | - | 2 | - | 1 | 1 | 1 | 1 |
| Scrub and or herbaceous vegetation associations | 1 | 2 | 2 | 1 | 1 | 1 | 1 | 1 | 1 |
| Urban fabric | 3 | 7 | 6 | 12 | 6 | 15 | 15 | 18 | 19 |
| Total | 42 | 55 | 33 | 60 | 38 | 65 | 66 | 65 | 72 |
| * This class include traps located in close proximity of lagoon areas | | | | | | | | | |

**Figure S1.** *Post-hoc* Tukey’s comparisons between years for average mosquitoes abundance, density-dependent variable and environmental variables

In each matrix the differences between the averages recorded for each year of analysis are displayed. The matrices must be read from the left to the right, the differences are to be considered as ‘diff. = row – column’. Only statistically significant pairwise comparisons (*P* ≤ 0.05) are showed; the remaining empty cells indicate that the difference is not significant. The colour intensity of the circles reflects the sign and the magnitude of the calculated difference (blue = negative; orange = positive, as reported in the scale bar); the size of the circles refers to the *P*-value of the differences (the lowest are the *P*-values, the larger are the circles).


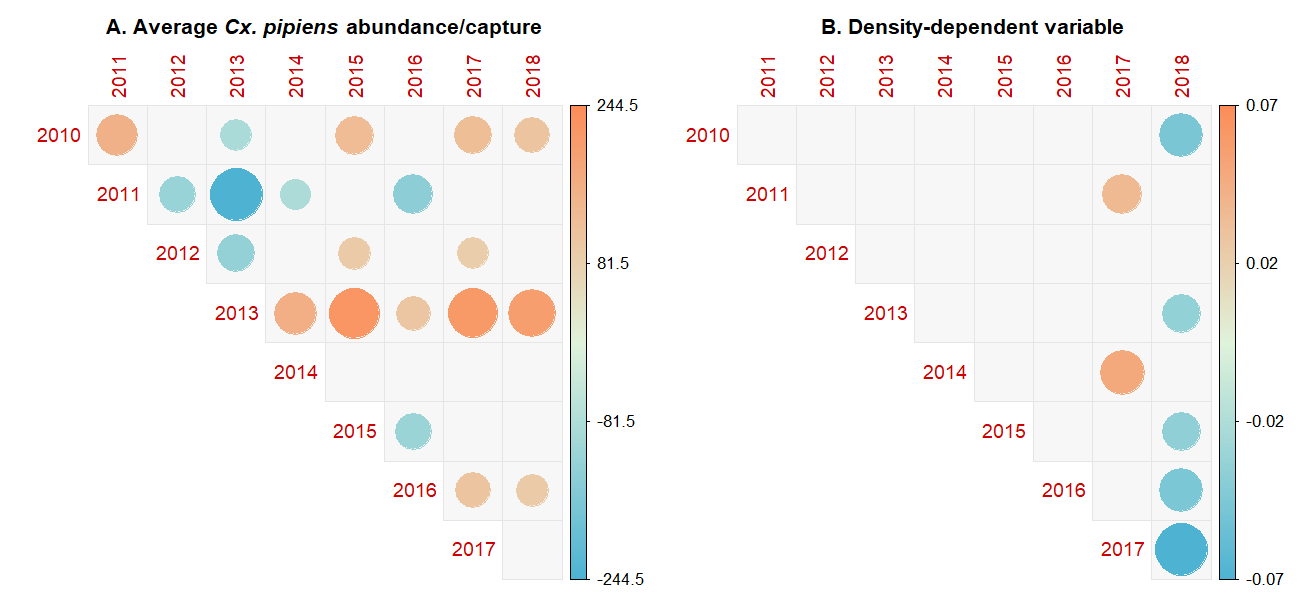


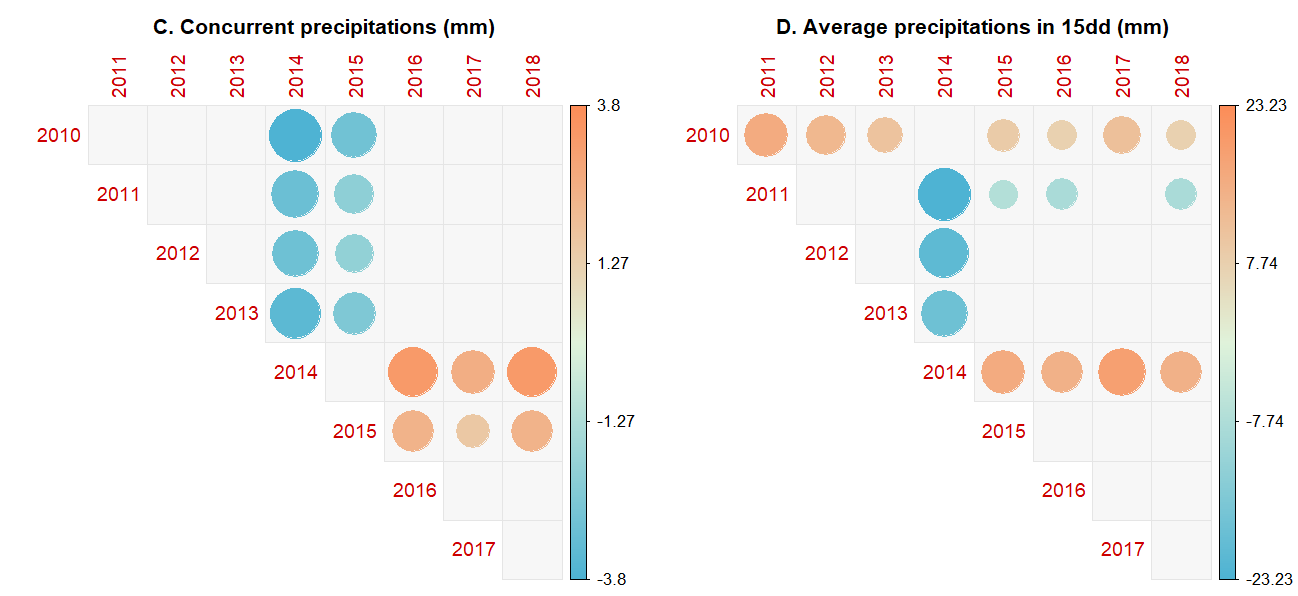


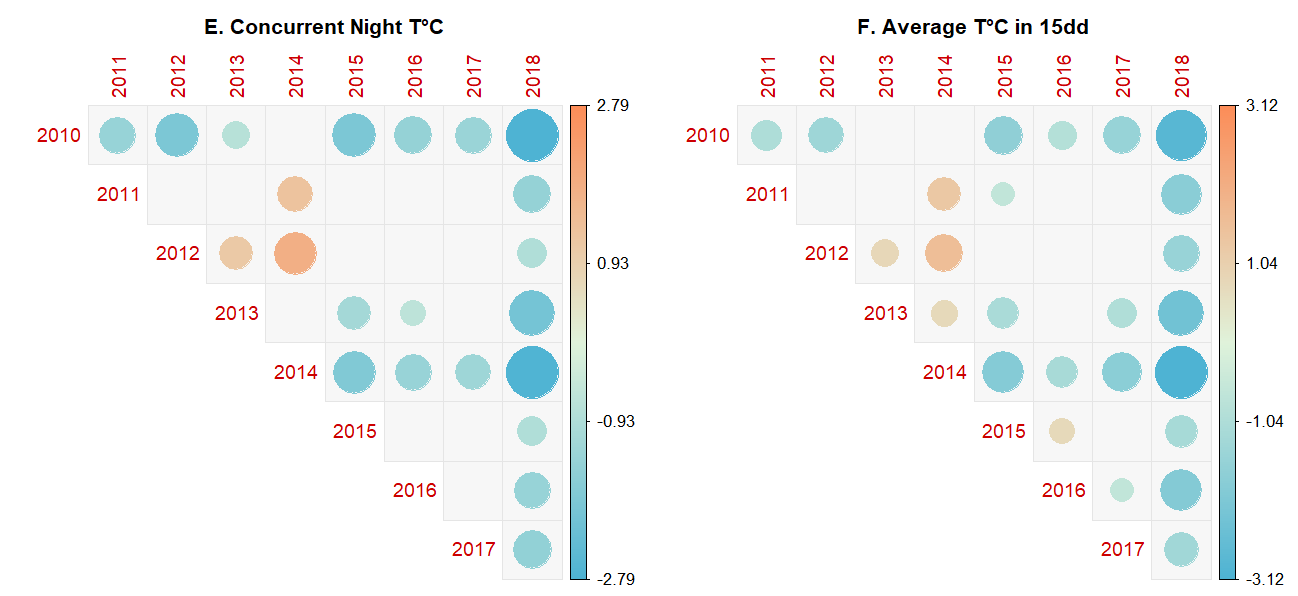

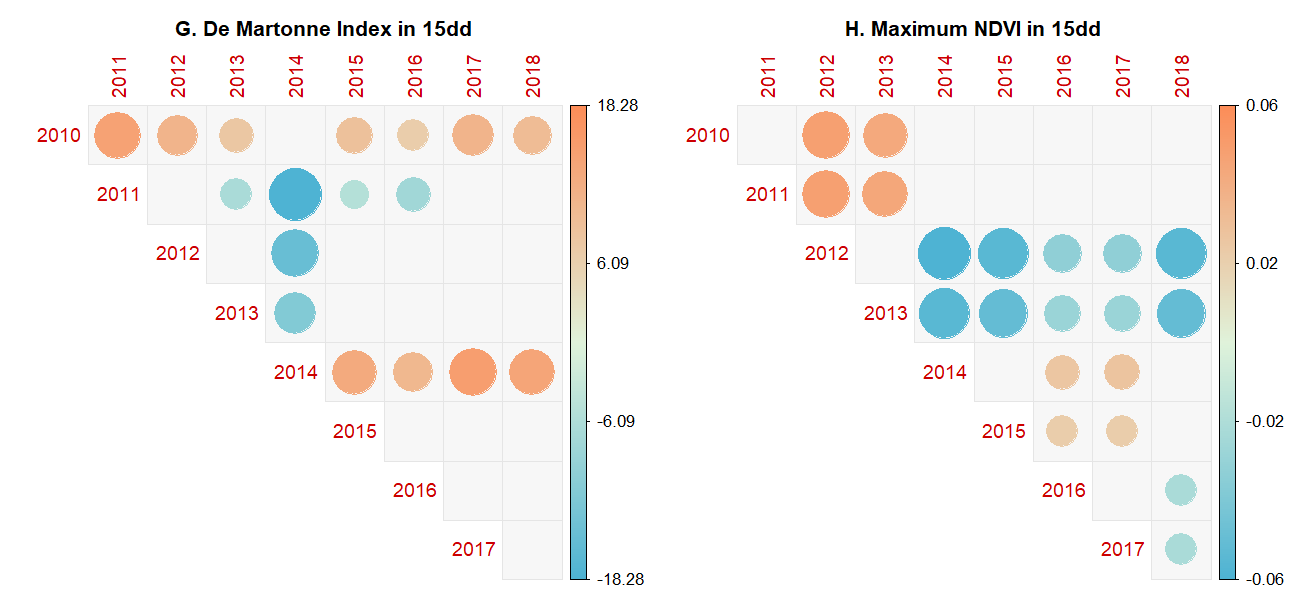


**Figure S2.** Annual trends of the level of aridity (DMI), normalized difference vegetation index (NDVI) and temperature (maximum, red line; minimum, blue line, LST) from 2010 to 2018. Gray lines: monthly averages for the whole study period


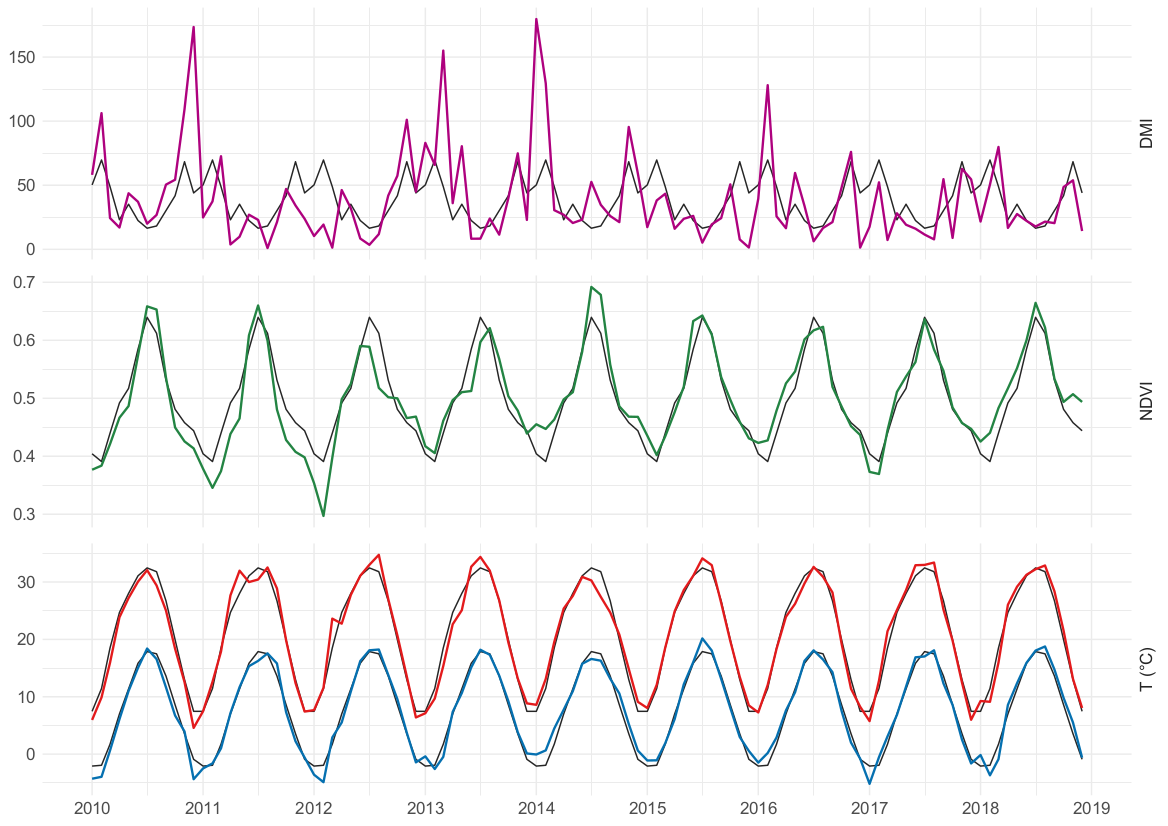


**Table S2.** Correlation matrix of intrinsic and extrinsic variables included in the analyses (absolute correlation values > 0.65 are reported in boldface)

|  | DMI_15d_ | PREC.sd_15d_ | PREC.k_15d_ | PREC.avg_15d_ | T.sd_15d_ | T.k_15d_ | T.avg_15d_ | PREC | GDD_15d_ | NDVI_15d_ | DT.h | GMP | T.night |
| --- | --- | --- | --- | --- | --- | --- | --- | --- | --- | --- | --- | --- | --- |
| DMI_15d_ | - | **0.89** | -0.32 | **0.99** | -0.15 | 0.17 | -0.44 | 0.28 | -0.44 | 0.06 | -0.12 | -0.20 | -0.32 |
| PREC.sd_15d_ | **0.89** | - | -0.04 | **0.90** | -0.09 | 0.13 | -0.30 | 0.31 | -0.30 | 0.09 | -0.11 | -0.12 | -0.24 |
| PREC.k_15d_ | -0.32 | -0.04 | - | -0.34 | 0.10 | -0.11 | 0.14 | -0.04 | 0.14 | -0.07 | -0.06 | 0.03 | 0.04 |
| PREC.avg_15d_ | **0.99** | **0.90** | -0.34 | - | -0.15 | 0.17 | -0.33 | 0.29 | -0.33 | 0.11 | -0.04 | -0.12 | -0.23 |
| T.sd_15d_ | -0.15 | -0.09 | 0.10 | -0.15 | - | -0.22 | 0.15 | -0.03 | 0.15 | -0.13 | 0.19 | 0.00 | 0.07 |
| T.k_15d_ | 0.17 | 0.13 | -0.11 | 0.17 | -0.22 | - | -0.17 | 0.02 | -0.17 | 0.04 | -0.09 | -0.09 | -0.07 |
| T.avg_15d_ | -0.44 | -0.30 | 0.14 | -0.33 | 0.15 | -0.17 | - | -0.07 | **1.00** | 0.33 | 0.62 | **0.66** | **0.82** |
| PREC | 0.28 | 0.31 | -0.04 | 0.29 | -0.03 | 0.02 | -0.07 | - | -0.07 | 0.03 | -0.06 | -0.02 | -0.13 |
| GDD_15d_ | -0.44 | -0.30 | 0.14 | -0.33 | 0.15 | -0.17 | **1.00** | -0.07 | - | 0.33 | 0.62 | **0.66** | **0.81** |
| NDVI_15d_ | 0.06 | 0.09 | -0.07 | 0.11 | -0.13 | 0.04 | 0.33 | 0.03 | 0.33 | - | 0.33 | 0.42 | 0.37 |
| DT.h | -0.12 | -0.11 | -0.06 | -0.04 | 0.19 | -0.09 | 0.62 | -0.06 | 0.62 | 0.33 | - | 0.52 | **0.67** |
| GMP | -0.20 | -0.12 | 0.03 | -0.12 | 0.00 | -0.09 | **0.66** | -0.02 | **0.66** | 0.42 | 0.52 | - | 0.61 |
| T.night | -0.32 | -0.24 | 0.04 | -0.23 | 0.07 | -0.07 | **0.82** | -0.13 | **0.81** | 0.37 | **0.67** | 0.61 | - |
