# Supplementary material for: Inter-annual variability of the effects of intrinsic and extrinsic drivers affecting West Nile virus vector Culex pipiens population dynamics in northeastern Italy
Source: Parasit Vectors. 2020 May 29;13:271. doi: 10.1186/s13071-020-04143-w (PMC7260749; doi:10.1186/s13071-020-04143-w)
Supplement: Supplementary file 2 — Additional file 2: Table S3. Lists of models per year included in the subsets considered in the study; models are ordered by decreasing wAICc and likelihood-ratio-based pseudo-R2 ($R^{2}{_{\text{LR}} }$R2LR) is reported to indicate the goodness-of-fit of each model. [file 13071_2020_4143_MOESM2_ESM.pdf]

Additional file 2: Table S3. Lists of models per year included in the subsets considered in the study; models are ordered by decreasing $wAIC_c$ and likelihood-ratio-based pseudo-R^2 (R^2_{LR}) is reported to indicate the goodness-of-fit of each model

2010-2018

Rank	Model	ΔAIC_c	$wAIC_c$	Cumulative $wAIC_c$	R_{LR}^2
1	Growth Rate ~ Intercept + DMI15d + GMP + DT.h + PREC.k15d + T.sd15d + GDD15d + NDVI15d	0.000	0.061	0.058	0.75
2	Growth Rate ~ Intercept + DMI15d + GMP + DT.h + PREC.k15d + T.sd15d + NDVI15d	0.572	0.046	0.101	0.75
3	Growth Rate ~ Intercept + DMI15d + GMP + DT.h + PREC.k15d + T.sd15d + T.night + GDD15d + NDVI15d	0.704	0.043	0.142	0.75
4	Growth Rate ~ Intercept + DMI15d + GMP + DT.h + T.sd15d + GDD15d + NDVI15d	0.721	0.042	0.182	0.75
5	Growth Rate ~ Intercept + DMI15d + GMP + DT.h + PREC.k15d + GDD15d + NDVI15d	0.774	0.041	0.221	0.75
6	Growth Rate ~ Intercept + DMI15d + GMP + DT.h + PREC.k15d + T.night + GDD15d + NDVI15d	0.842	0.040	0.259	0.75
7	Growth Rate ~ Intercept + DMI15d + GMP + DT.h + T.sd15d + T.night + GDD15d + NDVI15d	1.336	0.031	0.288	0.75
8	Growth Rate ~ Intercept + DMI15d + GMP + DT.h + PREC.k15d + T.k15d + T.sd15d + GDD15d + NDVI15d	1.424	0.030	0.317	0.75
9	Growth Rate ~ Intercept + DMI15d + GMP + DT.h + T.night + GDD15d + NDVI15d	1.467	0.029	0.344	0.75
10	Growth Rate ~ Intercept + DMI15d + GMP + DT.h + GDD15d + NDVI15d	1.508	0.029	0.371	0.75
11	Growth Rate ~ Intercept + DMI15d + GMP + DT.h + PREC.k15d + T.sd15d + T.night + NDVI15d	1.837	0.024	0.394	0.75
12	Growth Rate ~ Intercept + DMI15d + GMP + DT.h + PREC.k15d + T.k15d + T.sd15d + T.night + GDD15d + NDVI15d	1.867	0.024	0.417	0.75
13	Growth Rate ~ Intercept + DMI15d + GMP + DT.h + PREC + PREC.k15d + T.sd15d + GDD15d + NDVI15d	1.983	0.023	0.438	0.75
14	Growth Rate ~ Intercept + DMI15d + GMP + DT.h + PREC.k15d + NDVI15d	2.061	0.022	0.459	0.75
15	Growth Rate ~ Intercept + DMI15d + GMP + DT.h + PREC.k15d + T.k15d + T.sd15d + NDVI15d	2.108	0.021	0.479	0.75
16	Growth Rate ~ Intercept + DMI15d + GMP + DT.h + T.k15d + T.sd15d + GDD15d + NDVI15d	2.295	0.019	0.497	0.75
17	Growth Rate ~ Intercept + DMI15d + GMP + DT.h + T.sd15d + NDVI15d	2.439	0.018	0.514	0.75
18	Growth Rate ~ Intercept + DMI15d + GMP + DT.h + PREC.k15d + T.k15d + T.night + GDD15d + NDVI15d	2.458	0.018	0.531	0.75
19	Growth Rate ~ Intercept + DMI15d + GMP + DT.h + PREC + PREC.k15d + T.sd15d + T.night + GDD15d + NDVI15d	2.583	0.017	0.547	0.75
20	Growth Rate ~ Intercept + DMI15d + GMP + DT.h + PREC + PREC.k15d + T.sd15d + NDVI15d	2.584	0.017	0.563	0.75
21	Growth Rate ~ Intercept + DMI15d + GMP + DT.h + PREC.k15d + T.k15d + GDD15d + NDVI15d	2.623	0.016	0.579	0.75
22	Growth Rate ~ Intercept + DMI15d + GMP + DT.h + T.k15d + T.sd15d + T.night + GDD15d + NDVI15d	2.672	0.016	0.594	0.75
23	Growth Rate ~ Intercept + DMI15d + GMP + DT.h + PREC + PREC.k15d + T.night + GDD15d + NDVI15d	2.705	0.016	0.609	0.75
24	Growth Rate ~ Intercept + DMI15d + GMP + DT.h + PREC + T.sd15d + GDD15d + NDVI15d	2.714	0.016	0.623	0.75
25	Growth Rate ~ Intercept + DMI15d + GMP + DT.h + PREC + PREC.k15d + GDD15d + NDVI15d	2.764	0.015	0.638	0.75
26	Growth Rate ~ Intercept + DMI15d + GMP + DT.h + PREC.k15d + T.night + NDVI15d	2.827	0.015	0.652	0.75
27	Growth Rate ~ Intercept + DMI15d + GMP + DT.h + T.k15d + T.night + GDD15d + NDVI15d	3.196	0.012	0.664	0.75
28	Growth Rate ~ Intercept + DMI15d + GMP + DT.h + PREC.k15d + T.k15d + T.sd15d + T.night + NDVI15d	3.214	0.012	0.675	0.75
29	Growth Rate ~ Intercept + DMI15d + GMP + DT.h + PREC + T.sd15d + T.night + GDD15d + NDVI15d	3.233	0.012	0.687	0.75
30	Growth Rate ~ Intercept + DMI15d + GMP + DT.h + PREC + T.night + GDD15d + NDVI15d	3.350	0.011	0.697	0.75
31	Growth Rate ~ Intercept + DMI15d + GMP + DT.h + PREC + PREC.k15d + T.k15d + T.sd15d + GDD15d + NDVI15d	3.416	0.011	0.708	0.75
32	Growth Rate ~ Intercept + DMI15d + GMP + DT.h + T.k15d + GDD15d + NDVI15d	3.430	0.011	0.718	0.75
33	Growth Rate ~ Intercept + DMI15d + GMP + DT.h + PREC + GDD15d + NDVI15d	3.506	0.011	0.728	0.75
34	Growth Rate ~ Intercept + DMI15d + GMP + DT.h + T.sd15d + T.night + NDVI15d	3.724	0.009	0.737	0.75

2010-2018

Rank	Model	ΔAIC_c	$wAIC_c$	Cumulative $wAIC_c$	R_{LR}^2
35	Growth Rate ~ Intercept + DMI15d + GMP + DT.h + PREC + PREC.k15d + T.k15d + T.sd15d + T.night + GDD15d + NDVI15d	3.756	0.009	0.746	0.75
36	Growth Rate ~ Intercept + DMI15d + GMP + DT.h + PREC + PREC.k15d + T.sd15d + T.night + NDVI15d	3.839	0.009	0.754	0.75
37	Growth Rate ~ Intercept + DMI15d + GMP + DT.h + PREC.k15d + T.k15d + NDVI15d	3.999	0.008	0.762	0.75
38	Growth Rate ~ Intercept + DMI15d + GMP + DT.h + PREC + PREC.k15d + NDVI15d	4.067	0.008	0.770	0.75
39	Growth Rate ~ Intercept + DMI15d + GMP + DT.h + NDVI15d	4.110	0.008	0.777	0.75
40	Growth Rate ~ Intercept + DMI15d + GMP + DT.h + PREC + PREC.k15d + T.k15d + T.sd15d + NDVI15d	4.119	0.008	0.785	0.75
41	Growth Rate ~ Intercept + DMI15d + GMP + DT.h + T.k15d + T.sd15d + NDVI15d	4.152	0.008	0.792	0.75
42	Growth Rate ~ Intercept + DMI15d + GMP + DT.h + PREC + T.k15d + T.sd15d + GDD15d + NDVI15d	4.294	0.007	0.799	0.75
43	Growth Rate ~ Intercept + GMP + DT.h + T.sd15d + NDVI15d	4.323	0.007	0.805	0.75
44	Growth Rate ~ Intercept + DMI15d + GMP + DT.h + PREC + PREC.k15d + T.k15d + T.night + GDD15d + NDVI15d	4.328	0.007	0.812	0.75
45	Growth Rate ~ Intercept + DMI15d + GMP + DT.h + PREC + T.sd15d + NDVI15d	4.440	0.007	0.818	0.75
46	Growth Rate ~ Intercept + DMI15d + GMP + DT.h + PREC + T.k15d + T.sd15d + T.night + GDD15d + NDVI15d	4.578	0.006	0.824	0.75
47	Growth Rate ~ Intercept + DMI15d + GMP + DT.h + PREC + PREC.k15d + T.k15d + GDD15d + NDVI15d	4.618	0.006	0.830	0.75
48	Growth Rate ~ Intercept + DMI15d + GMP + DT.h + PREC.k15d + T.k15d + T.night + NDVI15d	4.648	0.006	0.835	0.75
49	Growth Rate ~ Intercept + DMI15d + GMP + DT.h + PREC + PREC.k15d + T.night + NDVI15d	4.831	0.005	0.840	0.75
50	Growth Rate ~ Intercept + DMI15d + GMP + DT.h + T.night + NDVI15d	4.887	0.005	0.845	0.75
51	Growth Rate ~ Intercept + GMP + DT.h + T.sd15d + T.night + NDVI15d	4.921	0.005	0.850	0.75
52	Growth Rate ~ Intercept + GMP + DT.h + PREC.k15d + T.sd15d + NDVI15d	4.971	0.005	0.855	0.75
53	Growth Rate ~ Intercept + DMI15d + GMP + DT.h + PREC + T.k15d + T.night + GDD15d + NDVI15d	5.083	0.005	0.860	0.75
54	Growth Rate ~ Intercept + DMI15d + GMP + DT.h + PREC + PREC.k15d + T.k15d + T.sd15d + T.night + NDVI15d	5.221	0.004	0.864	0.75
55	Growth Rate ~ Intercept + DMI15d + GMP + DT.h + T.k15d + T.sd15d + T.night + NDVI15d	5.313	0.004	0.868	0.75
56	Growth Rate ~ Intercept + GMP + DT.h + PREC.k15d + T.sd15d + T.night + NDVI15d	5.372	0.004	0.872	0.75
57	Growth Rate ~ Intercept + GMP + DT.h + NDVI15d	5.379	0.004	0.876	0.75
58	Growth Rate ~ Intercept + DMI15d + GMP + DT.h + PREC + T.k15d + GDD15d + NDVI15d	5.431	0.004	0.880	0.75
59	Growth Rate ~ Intercept + GMP + DT.h + T.night + NDVI15d	5.448	0.004	0.883	0.75
60	Growth Rate ~ Intercept + GMP + DT.h + T.night + GDD15d + NDVI15d	5.495	0.004	0.887	0.75
61	Growth Rate ~ Intercept + GMP + DT.h + T.sd15d + GDD15d + NDVI15d	5.617	0.004	0.891	0.75
62	Growth Rate ~ Intercept + GMP + DT.h + T.sd15d + T.night + GDD15d + NDVI15d	5.635	0.004	0.894	0.75
63	Growth Rate ~ Intercept + GMP + DT.h + PREC.k15d + T.night + NDVI15d	5.652	0.004	0.897	0.75
64	Growth Rate ~ Intercept + DMI15d + GMP + DT.h + PREC + T.sd15d + T.night + NDVI15d	5.736	0.003	0.901	0.75
65	Growth Rate ~ Intercept + GMP + DT.h + T.k15d + T.sd15d + NDVI15d	5.761	0.003	0.904	0.75
66	Growth Rate ~ Intercept + GMP + DT.h + PREC.k15d + NDVI15d	5.805	0.003	0.907	0.75
67	Growth Rate ~ Intercept + DMI15d + GMP + DT.h + PREC + PREC.k15d + T.k15d + NDVI15d	6.004	0.003	0.910	0.75
68	Growth Rate ~ Intercept + GMP + DT.h + PREC + T.sd15d + NDVI15d	6.065	0.003	0.913	0.75
69	Growth Rate ~ Intercept + DMI15d + GMP + DT.h + PREC + NDVI15d	6.096	0.003	0.916	0.75
70	Growth Rate ~ Intercept + DMI15d + GMP + DT.h + T.k15d + NDVI15d	6.107	0.003	0.918	0.75
71	Growth Rate ~ Intercept + DMI15d + GMP + DT.h + PREC + T.k15d + T.sd15d + NDVI15d	6.148	0.003	0.921	0.75
72	Growth Rate ~ Intercept + GMP + DT.h + T.k15d + T.sd15d + T.night + NDVI15d	6.168	0.003	0.924	0.75

2010-2018

Rank	Model	ΔAIC$_c$	wAIC$_c$	Cumulative wAIC$_c$	R_{LR}^2
73	Growth Rate ~ Intercept + GMP + DT.h + GDD15d + NDVI15d	6.191	0.003	0.926	0.75
74	Growth Rate ~ Intercept + GMP + DT.h + PREC.k15d + T.k15d + T.sd15d + NDVI15d	6.202	0.003	0.929	0.75
75	Growth Rate ~ Intercept + GMP + DT.h + PREC.k15d + T.k15d + T.sd15d + T.night + NDVI15d	6.343	0.003	0.931	0.75
76	Growth Rate ~ Intercept + GMP + DT.h + PREC.k15d + T.night + GDD15d + NDVI15d	6.574	0.002	0.933	0.75
77	Growth Rate ~ Intercept + GMP + DT.h + T.k15d + T.sd15d + T.night + GDD15d + NDVI15d	6.592	0.002	0.935	0.75
78	Growth Rate ~ Intercept + DMI15d + GMP + DT.h + PREC + PREC.k15d + T.k15d + T.night + NDVI15d	6.655	0.002	0.938	0.75
79	Growth Rate ~ Intercept + GMP + DT.h + PREC + PREC.k15d + T.sd15d + NDVI15d	6.670	0.002	0.940	0.75
80	Growth Rate ~ Intercept + GMP + DT.h + PREC.k15d + T.sd15d + GDD15d + NDVI15d	6.715	0.002	0.942	0.75
81	Growth Rate ~ Intercept + GMP + DT.h + PREC.k15d + T.sd15d + T.night + GDD15d + NDVI15d	6.736	0.002	0.944	0.75
82	Growth Rate ~ Intercept + DMI15d + GMP + DT.h + T.k15d + T.night + NDVI15d	6.819	0.002	0.945	0.75
83	Growth Rate ~ Intercept + GMP + DT.h + PREC + T.sd15d + T.night + NDVI15d	6.833	0.002	0.947	0.75
84	Growth Rate ~ Intercept + DMI15d + GMP + DT.h + PREC + T.night + NDVI15d	6.898	0.002	0.949	0.75

2010

Rank	Model	ΔAIC$_c$	wAIC$_c$	Cumulative wAIC$_c$	R_{LR}^2
1	Growth Rate ~ Intercept + DMI15d + GMP + DT.h + PREC + T.night	0.000	0.044	0.042	0.78
2	Growth Rate ~ Intercept + DMI15d + GMP + DT.h + PREC + T.sd15d + T.night	0.347	0.037	0.077	0.78
3	Growth Rate ~ Intercept + DMI15d + GMP + DT.h + T.night	0.431	0.036	0.111	0.78
4	Growth Rate ~ Intercept + GMP + DT.h + T.night	0.697	0.031	0.140	0.78
5	Growth Rate ~ Intercept + GMP + DT.h + PREC + T.night	0.756	0.030	0.169	0.78
6	Growth Rate ~ Intercept + DMI15d + GMP + DT.h + T.sd15d + T.night	1.158	0.025	0.192	0.78
7	Growth Rate ~ Intercept + DMI15d + GMP + DT.h + PREC.k15d + T.night	1.243	0.024	0.215	0.78
8	Growth Rate ~ Intercept + GMP + DT.h + PREC + T.night + NDVI15d	1.257	0.024	0.237	0.78
9	Growth Rate ~ Intercept + DMI15d + GMP + DT.h + PREC + T.night + NDVI15d	1.263	0.023	0.259	0.78
10	Growth Rate ~ Intercept + DMI15d + GMP + DT.h + PREC + PREC.k15d + T.night	1.437	0.021	0.280	0.78
11	Growth Rate ~ Intercept + GMP + DT.h + T.night + NDVI15d	1.442	0.021	0.300	0.78
12	Growth Rate ~ Intercept + DMI15d + GMP + DT.h + PREC + T.night + GDD15d	1.586	0.020	0.319	0.78
13	Growth Rate ~ Intercept + DMI15d + GMP + DT.h + PREC + T.sd15d + T.night + NDVI15d	1.632	0.019	0.337	0.78
14	Growth Rate ~ Intercept + DMI15d + GMP + DT.h + PREC + T.sd15d + T.night + GDD15d	1.754	0.018	0.355	0.78
15	Growth Rate ~ Intercept + DMI15d + GMP + DT.h + T.night + NDVI15d	1.828	0.018	0.371	0.78
16	Growth Rate ~ Intercept + DMI15d + GMP + DT.h + PREC + PREC.k15d + T.sd15d + T.night	1.889	0.017	0.388	0.78
17	Growth Rate ~ Intercept + DMI15d + GMP + DT.h + PREC.k15d + T.sd15d + T.night	2.042	0.016	0.403	0.78
18	Growth Rate ~ Intercept + DMI15d + GMP + DT.h + T.night + GDD15d	2.099	0.015	0.417	0.78
19	Growth Rate ~ Intercept + DMI15d + GMP + DT.h + PREC + T.k15d + T.night	2.110	0.015	0.432	0.78
20	Growth Rate ~ Intercept + GMP + DT.h + T.k15d + T.night	2.366	0.014	0.445	0.78
21	Growth Rate ~ Intercept + DMI15d + GMP + DT.h + T.k15d + T.night	2.471	0.013	0.457	0.78
22	Growth Rate ~ Intercept + DMI15d + GMP + DT.h + PREC + T.k15d + T.sd15d + T.night	2.479	0.013	0.469	0.78
23	Growth Rate ~ Intercept + DMI15d + GMP + DT.h + T.sd15d + T.night + NDVI15d	2.590	0.012	0.480	0.78
24	Growth Rate ~ Intercept + DMI15d + GMP + DT.h + PREC.k15d + T.night + NDVI15d	2.690	0.011	0.491	0.78
25	Growth Rate ~ Intercept + GMP + DT.h + T.night + GDD15d	2.707	0.011	0.502	0.78
26	Growth Rate ~ Intercept + DMI15d + GMP + DT.h + T.sd15d + T.night + GDD15d	2.713	0.011	0.513	0.78
27	Growth Rate ~ Intercept + GMP + DT.h + PREC + T.night + GDD15d	2.755	0.011	0.523	0.78
28	Growth Rate ~ Intercept + DMI15d + GMP + DT.h + PREC + PREC.k15d + T.night + NDVI15d	2.756	0.011	0.534	0.78
29	Growth Rate ~ Intercept + GMP + DT.h + PREC + T.k15d + T.night	2.784	0.011	0.544	0.78
30	Growth Rate ~ Intercept + GMP + DT.h + PREC.k15d + T.night	2.799	0.011	0.555	0.78
31	Growth Rate ~ Intercept + GMP + DT.h + T.sd15d + T.night	2.816	0.011	0.565	0.78
32	Growth Rate ~ Intercept + GMP + DT.h + PREC + PREC.k15d + T.night	2.844	0.011	0.575	0.78
33	Growth Rate ~ Intercept + DMI15d + GMP + DT.h + PREC + T.night + GDD15d + NDVI15d	2.866	0.011	0.585	0.78
34	Growth Rate ~ Intercept + GMP + DT.h + PREC + T.sd15d + T.night	2.885	0.010	0.595	0.78
35	Growth Rate ~ Intercept + DMI15d + GMP + DT.h + T.k15d + T.sd15d + T.night	2.943	0.010	0.604	0.78
36	Growth Rate ~ Intercept + DMI15d + GMP + DT.h + PREC.k15d + T.night + GDD15d	3.024	0.010	0.614	0.78

2010

Rank	Model	ΔAIC_c	$wAIC_c$	Cumulative $wAIC_c$	R_{LR}^2
37	Growth Rate ~ Intercept + DMI15d + GMP + DT.h + PREC + T.sd15d + T.night + GDD15d + NDVI15d	3.045	0.010	0.623	0.79
38	Growth Rate ~ Intercept + GMP + DT.h + T.k15d + T.night + NDVI15d	3.056	0.010	0.632	0.78
39	Growth Rate ~ Intercept + DMI15d + GMP + DT.h + PREC + PREC.k15d + T.night + GDD15d	3.128	0.009	0.640	0.78
40	Growth Rate ~ Intercept + DMI15d + GMP + DT.h + PREC + PREC.k15d + T.sd15d + T.night + NDVI15d	3.224	0.009	0.649	0.79
41	Growth Rate ~ Intercept + DMI15d + GMP + DT.h + PREC.k15d + T.k15d + T.night	3.257	0.009	0.657	0.78
42	Growth Rate ~ Intercept + GMP + DT.h + PREC + T.k15d + T.night + NDVI15d	3.278	0.009	0.665	0.78
43	Growth Rate ~ Intercept + GMP + DT.h + PREC + T.night + GDD15d + NDVI15d	3.348	0.008	0.673	0.78
44	Growth Rate ~ Intercept + GMP + DT.h + PREC + T.sd15d + T.night + NDVI15d	3.372	0.008	0.681	0.78
45	Growth Rate ~ Intercept + GMP + DT.h + PREC + PREC.k15d + T.night + NDVI15d	3.388	0.008	0.688	0.78
46	Growth Rate ~ Intercept + DMI15d + GMP + DT.h + PREC + T.k15d + T.night + NDVI15d	3.405	0.008	0.696	0.78
47	Growth Rate ~ Intercept + DMI15d + GMP + DT.h + PREC + PREC.k15d + T.sd15d + T.night + GDD15d	3.419	0.008	0.704	0.79
48	Growth Rate ~ Intercept + GMP + DT.h + PREC.k15d + T.night + NDVI15d	3.510	0.008	0.711	0.78
49	Growth Rate ~ Intercept + DMI15d + GMP + DT.h + PREC.k15d + T.sd15d + T.night + NDVI15d	3.518	0.008	0.718	0.78
50	Growth Rate ~ Intercept + DMI15d + GMP + DT.h + T.night + GDD15d + NDVI15d	3.520	0.008	0.725	0.78
51	Growth Rate ~ Intercept + GMP + DT.h + T.night + GDD15d + NDVI15d	3.530	0.008	0.732	0.78
52	Growth Rate ~ Intercept + GMP + DT.h + T.sd15d + T.night + NDVI15d	3.552	0.007	0.739	0.78
53	Growth Rate ~ Intercept + DMI15d + GMP + DT.h + PREC + PREC.k15d + T.k15d + T.night	3.580	0.007	0.746	0.78
54	Growth Rate ~ Intercept + DMI15d + GMP + DT.h + PREC + T.k15d + T.night + GDD15d	3.601	0.007	0.753	0.78
55	Growth Rate ~ Intercept + DMI15d + GMP + DT.h + PREC.k15d + T.sd15d + T.night + GDD15d	3.724	0.007	0.760	0.78
56	Growth Rate ~ Intercept + DMI15d + GMP + DT.h + PREC + T.k15d + T.sd15d + T.night + NDVI15d	3.747	0.007	0.766	0.78
57	Growth Rate ~ Intercept + DMI15d + GMP + DT.h + PREC.k15d + T.k15d + T.sd15d + T.night	3.775	0.007	0.773	0.78
58	Growth Rate ~ Intercept + DMI15d + GMP + DT.h + T.k15d + T.night + NDVI15d	3.820	0.007	0.779	0.78
59	Growth Rate ~ Intercept + DMI15d + GMP + DT.h + PREC + T.k15d + T.sd15d + T.night + GDD15d	3.910	0.006	0.785	0.78
60	Growth Rate ~ Intercept + DMI15d + GMP + DT.h + PREC + PREC.k15d + T.k15d + T.sd15d + T.night	4.003	0.006	0.790	0.78
61	Growth Rate ~ Intercept + DMI15d + GMP + DT.h + T.sd15d + T.night + GDD15d + NDVI15d	4.163	0.005	0.795	0.78
62	Growth Rate ~ Intercept + DMI15d + GMP + DT.h + T.k15d + T.night + GDD15d	4.215	0.005	0.801	0.78
63	Growth Rate ~ Intercept + DMI15d + GMP + DT.h + T.k15d + T.sd15d + T.night + NDVI15d	4.277	0.005	0.806	0.78
64	Growth Rate ~ Intercept + GMP + DT.h + T.k15d + T.night + GDD15d	4.375	0.005	0.810	0.78
65	Growth Rate ~ Intercept + GMP + DT.h + PREC.k15d + T.k15d + T.night	4.433	0.005	0.815	0.78
66	Growth Rate ~ Intercept + GMP + DT.h + T.k15d + T.sd15d + T.night	4.446	0.005	0.819	0.78
67	Growth Rate ~ Intercept + DMI15d + GMP + DT.h + PREC + PREC.k15d + T.night + GDD15d + NDVI15d	4.458	0.005	0.824	0.78
68	Growth Rate ~ Intercept + DMI15d + GMP + DT.h + PREC.k15d + T.night + GDD15d + NDVI15d	4.488	0.005	0.828	0.78
69	Growth Rate ~ Intercept + DMI15d + GMP + DT.h + PREC.k15d + T.k15d + T.night + NDVI15d	4.648	0.004	0.832	0.78
70	Growth Rate ~ Intercept + DMI15d + GMP + DT.h + T.k15d + T.sd15d + T.night + GDD15d	4.658	0.004	0.836	0.78
71	Growth Rate ~ Intercept + DMI15d + GMP + DT.h + PREC + PREC.k15d + T.sd15d + T.night + GDD15d + NDVI15d	4.752	0.004	0.840	0.79
72	Growth Rate ~ Intercept + GMP + DT.h + PREC.k15d + T.night + GDD15d	4.763	0.004	0.844	0.78

2010

Rank	Model	ΔAIC_c	$wAIC_c$	Cumulative $wAIC_c$	R_{LR}^2
73	Growth Rate ~ Intercept + GMP + DT.h + PREC + T.k15d + T.night + GDD15d	4.788	0.004	0.848	0.78
74	Growth Rate ~ Intercept + GMP + DT.h + T.sd15d + T.night + GDD15d	4.825	0.004	0.852	0.78
75	Growth Rate ~ Intercept + GMP + DT.h + PREC + T.sd15d + T.night + GDD15d	4.880	0.004	0.855	0.78
76	Growth Rate ~ Intercept + GMP + DT.h + PREC + PREC.k15d + T.night + GDD15d	4.889	0.004	0.859	0.78
77	Growth Rate ~ Intercept + GMP + DT.h + PREC + PREC.k15d + T.k15d + T.night	4.907	0.004	0.863	0.78
78	Growth Rate ~ Intercept + GMP + DT.h + PREC + T.k15d + T.sd15d + T.night	4.910	0.004	0.866	0.78
79	Growth Rate ~ Intercept + DMI15d + GMP + DT.h + PREC + PREC.k15d + T.k15d + T.night + NDVI15d	4.916	0.004	0.870	0.78
80	Growth Rate ~ Intercept + GMP + DT.h + PREC.k15d + T.sd15d + T.night	4.925	0.004	0.873	0.78
81	Growth Rate ~ Intercept + DMI15d + GMP + DT.h + PREC + T.k15d + T.night + GDD15d + NDVI15d	4.946	0.004	0.877	0.78
82	Growth Rate ~ Intercept + GMP + DT.h + PREC + PREC.k15d + T.sd15d + T.night	4.977	0.004	0.880	0.78
83	Growth Rate ~ Intercept + GMP + DT.h + T.k15d + T.sd15d + T.night + NDVI15d	5.041	0.004	0.884	0.78
84	Growth Rate ~ Intercept + GMP + DT.h + PREC.k15d + T.k15d + T.night + NDVI15d	5.051	0.004	0.887	0.78
85	Growth Rate ~ Intercept + DMI15d + GMP + DT.h + PREC.k15d + T.k15d + T.night + GDD15d	5.123	0.003	0.890	0.78
86	Growth Rate ~ Intercept + DMI15d + GMP + DT.h + PREC.k15d + T.k15d + T.sd15d + T.night + NDVI15d	5.149	0.003	0.893	0.78
87	Growth Rate ~ Intercept + GMP + DT.h + T.k15d + T.night + GDD15d + NDVI15d	5.149	0.003	0.897	0.78
88	Growth Rate ~ Intercept + DMI15d + GMP + DT.h + PREC.k15d + T.sd15d + T.night + GDD15d + NDVI15d	5.213	0.003	0.900	0.78
89	Growth Rate ~ Intercept + DMI15d + GMP + DT.h + PREC + T.sd15d	5.213	0.003	0.903	0.78
90	Growth Rate ~ Intercept + DMI15d + GMP + DT.h + PREC + T.k15d + T.sd15d + T.night + GDD15d + NDVI15d	5.215	0.003	0.906	0.79
91	Growth Rate ~ Intercept + DMI15d + GMP + DT.h + PREC + PREC.k15d + T.k15d + T.night + GDD15d	5.219	0.003	0.909	0.78
92	Growth Rate ~ Intercept + DMI15d + GMP + DT.h + PREC + PREC.k15d + T.k15d + T.sd15d + T.night + NDVI15d	5.310	0.003	0.912	0.79
93	Growth Rate ~ Intercept + GMP + DT.h + PREC + T.k15d + T.sd15d + T.night + NDVI15d	5.344	0.003	0.915	0.78
94	Growth Rate ~ Intercept + GMP + DT.h + PREC + T.k15d + T.night + GDD15d + NDVI15d	5.377	0.003	0.918	0.78
95	Growth Rate ~ Intercept + GMP + DT.h + PREC + PREC.k15d + T.k15d + T.night + NDVI15d	5.428	0.003	0.920	0.78
96	Growth Rate ~ Intercept + GMP + DT.h + PREC + T.sd15d + T.night + GDD15d + NDVI15d	5.444	0.003	0.923	0.78
97	Growth Rate ~ Intercept + DMI15d + GMP + DT.h + PREC + T.sd15d + GDD15d	5.458	0.003	0.926	0.78
98	Growth Rate ~ Intercept + GMP + DT.h + PREC + PREC.k15d + T.sd15d + T.night + NDVI15d	5.496	0.003	0.928	0.78
99	Growth Rate ~ Intercept + GMP + DT.h + PREC + PREC.k15d + T.night + GDD15d + NDVI15d	5.498	0.003	0.931	0.78
100	Growth Rate ~ Intercept + GMP + DT.h + PREC.k15d + T.night + GDD15d + NDVI15d	5.554	0.003	0.934	0.78
101	Growth Rate ~ Intercept + DMI15d + GMP + DT.h + PREC + PREC.k15d + T.k15d + T.sd15d + T.night + GDD15d	5.589	0.003	0.936	0.79
102	Growth Rate ~ Intercept + DMI15d + GMP + DT.h + T.k15d + T.night + GDD15d + NDVI15d	5.610	0.003	0.939	0.78
103	Growth Rate ~ Intercept + DMI15d + GMP + DT.h + PREC.k15d + T.k15d + T.sd15d + T.night + GDD15d	5.620	0.003	0.941	0.78
104	Growth Rate ~ Intercept + GMP + DT.h + T.sd15d + T.night + GDD15d + NDVI15d	5.627	0.003	0.944	0.78
105	Growth Rate ~ Intercept + GMP + DT.h + PREC.k15d + T.sd15d + T.night + NDVI15d	5.646	0.003	0.946	0.78
106	Growth Rate ~ Intercept + DMI15d + GMP + DT.h + T.k15d + T.sd15d + T.night + GDD15d + NDVI15d	6.031	0.002	0.948	0.78

2011

Rank	Model	ΔAIC_c	$wAIC_c$	Cumulative $wAIC_c$	R_{LR}^2
1	Growth Rate ~ Intercept + GMP + DT.h + GDD15d	0.000	0.039	0.037	0.70
2	Growth Rate ~ Intercept + GMP + DT.h + T.night + GDD15d	0.267	0.034	0.070	0.70
3	Growth Rate ~ Intercept + GMP + DT.h + T.k15d + GDD15d	0.293	0.034	0.102	0.70
4	Growth Rate ~ Intercept + GMP + DT.h + PREC.k15d + T.k15d + GDD15d	0.368	0.033	0.133	0.70
5	Growth Rate ~ Intercept + GMP + DT.h + T.k15d + T.night + GDD15d	0.739	0.027	0.159	0.70
6	Growth Rate ~ Intercept + GMP + DT.h + PREC.k15d + GDD15d	0.917	0.025	0.183	0.70
7	Growth Rate ~ Intercept + GMP + DT.h + PREC.k15d + T.k15d + T.night + GDD15d	1.342	0.020	0.202	0.70
8	Growth Rate ~ Intercept + DMI15d + GMP + DT.h + T.night + GDD15d	1.527	0.018	0.219	0.70
9	Growth Rate ~ Intercept + GMP + DT.h + PREC.k15d + T.night + GDD15d	1.581	0.018	0.236	0.70
10	Growth Rate ~ Intercept + GMP + DT.h + PREC + GDD15d	1.596	0.018	0.253	0.70
11	Growth Rate ~ Intercept + DMI15d + GMP + DT.h + PREC.k15d + GDD15d	1.625	0.017	0.269	0.70
12	Growth Rate ~ Intercept + DMI15d + GMP + DT.h + PREC.k15d + T.night + GDD15d	1.699	0.017	0.285	0.70
13	Growth Rate ~ Intercept + DMI15d + GMP + DT.h + GDD15d	1.741	0.016	0.301	0.70
14	Growth Rate ~ Intercept + DMI15d + GMP + DT.h + PREC.k15d + T.k15d + GDD15d	1.816	0.016	0.316	0.70
15	Growth Rate ~ Intercept + GMP + DT.h + PREC + T.k15d + GDD15d	1.838	0.016	0.331	0.70
16	Growth Rate ~ Intercept + GMP + DT.h + T.sd15d + GDD15d	1.870	0.015	0.345	0.70
17	Growth Rate ~ Intercept + GMP + DT.h + GDD15d + NDVI15d	2.083	0.014	0.358	0.70
18	Growth Rate ~ Intercept + GMP + DT.h + PREC.k15d + T.k15d + T.sd15d + GDD15d	2.280	0.013	0.370	0.70
19	Growth Rate ~ Intercept + GMP + DT.h + PREC + T.night + GDD15d	2.283	0.013	0.382	0.70
20	Growth Rate ~ Intercept + GMP + DT.h + PREC + PREC.k15d + T.k15d + GDD15d	2.306	0.012	0.394	0.70
21	Growth Rate ~ Intercept + GMP + DT.h + T.sd15d + T.night + GDD15d	2.328	0.012	0.406	0.70
22	Growth Rate ~ Intercept + DMI15d + GMP + DT.h + T.k15d + GDD15d	2.357	0.012	0.417	0.70
23	Growth Rate ~ Intercept + GMP + DT.h + T.night + GDD15d + NDVI15d	2.360	0.012	0.429	0.70
24	Growth Rate ~ Intercept + GMP + DT.h + PREC.k15d + T.sd15d + GDD15d	2.361	0.012	0.440	0.70
25	Growth Rate ~ Intercept + GMP + DT.h + T.k15d + GDD15d + NDVI15d	2.362	0.012	0.452	0.70
26	Growth Rate ~ Intercept + DMI15d + GMP + DT.h + PREC.k15d + T.k15d + T.night + GDD15d	2.363	0.012	0.463	0.71
27	Growth Rate ~ Intercept + GMP + DT.h + T.k15d + T.sd15d + GDD15d	2.382	0.012	0.474	0.70
28	Growth Rate ~ Intercept + GMP + DT.h + PREC.k15d + T.k15d + GDD15d + NDVI15d	2.414	0.012	0.485	0.70
29	Growth Rate ~ Intercept + DMI15d + GMP + DT.h + T.k15d + T.night + GDD15d	2.572	0.011	0.496	0.70
30	Growth Rate ~ Intercept + GMP + DT.h + PREC + T.k15d + T.night + GDD15d	2.724	0.010	0.505	0.70
31	Growth Rate ~ Intercept + GMP + DT.h + PREC + PREC.k15d + GDD15d	2.810	0.010	0.515	0.70
32	Growth Rate ~ Intercept + GMP + DT.h + T.k15d + T.sd15d + T.night + GDD15d	2.812	0.010	0.524	0.70
33	Growth Rate ~ Intercept + GMP + DT.h + T.k15d + T.night + GDD15d + NDVI15d	2.843	0.009	0.533	0.70
34	Growth Rate ~ Intercept + DMI15d + GMP + DT.h + PREC + GDD15d	2.914	0.009	0.541	0.70
35	Growth Rate ~ Intercept + GMP + DT.h + PREC.k15d + GDD15d + NDVI15d	3.000	0.009	0.550	0.70
36	Growth Rate ~ Intercept + DMI15d + GMP + DT.h + PREC.k15d + T.sd15d + GDD15d	3.018	0.009	0.558	0.70
37	Growth Rate ~ Intercept + DMI15d + GMP + DT.h + PREC + PREC.k15d + GDD15d	3.094	0.008	0.566	0.70

2011

Rank	Model	ΔAIC$_c$	wAIC$_c$	Cumulative wAIC$_c$	R_{LR}^2
38	Growth Rate ~ Intercept + GMP + DT.h + T.night	3.277	0.008	0.573	0.70
39	Growth Rate ~ Intercept + DMI15d + GMP + DT.h + PREC + T.night + GDD15d	3.299	0.008	0.580	0.70
40	Growth Rate ~ Intercept + GMP + DT.h + PREC.k15d + T.night	3.370	0.007	0.587	0.70
41	Growth Rate ~ Intercept + GMP + DT.h + PREC.k15d + T.k15d + T.sd15d + T.night + GDD15d	3.427	0.007	0.594	0.70
42	Growth Rate ~ Intercept + GMP + DT.h + PREC.k15d + T.sd15d + T.night + GDD15d	3.427	0.007	0.601	0.70
43	Growth Rate ~ Intercept + GMP + DT.h + PREC.k15d + T.k15d + T.night + GDD15d + NDVI15d	3.442	0.007	0.607	0.70
44	Growth Rate ~ Intercept + GMP + DT.h + PREC + PREC.k15d + T.k15d + T.night + GDD15d	3.446	0.007	0.614	0.70
45	Growth Rate ~ Intercept + DMI15d + GMP + DT.h + PREC + PREC.k15d + T.k15d + GDD15d	3.489	0.007	0.620	0.70
46	Growth Rate ~ Intercept + GMP + DT.h + PREC + T.sd15d + GDD15d	3.519	0.007	0.627	0.70
47	Growth Rate ~ Intercept + DMI15d + GMP + DT.h + PREC.k15d + T.sd15d + T.night + GDD15d	3.578	0.007	0.633	0.70
48	Growth Rate ~ Intercept + DMI15d + GMP + DT.h + PREC + PREC.k15d + T.night + GDD15d	3.616	0.006	0.639	0.70
49	Growth Rate ~ Intercept + DMI15d + GMP + DT.h + T.night + GDD15d + NDVI15d	3.623	0.006	0.645	0.70
50	Growth Rate ~ Intercept + DMI15d + GMP + DT.h + T.sd15d + T.night + GDD15d	3.630	0.006	0.651	0.70
51	Growth Rate ~ Intercept + DMI15d + GMP + DT.h + PREC.k15d + T.k15d + T.sd15d + GDD15d	3.660	0.006	0.657	0.70
52	Growth Rate ~ Intercept + DMI15d + GMP + DT.h + T.sd15d + GDD15d	3.668	0.006	0.663	0.70
53	Growth Rate ~ Intercept + GMP + DT.h + PREC + PREC.k15d + T.night + GDD15d	3.676	0.006	0.669	0.70
54	Growth Rate ~ Intercept + GMP + DT.h + PREC.k15d + T.night + GDD15d + NDVI15d	3.686	0.006	0.675	0.70
55	Growth Rate ~ Intercept + GMP + DT.h + PREC + GDD15d + NDVI15d	3.690	0.006	0.681	0.70
56	Growth Rate ~ Intercept + DMI15d + GMP + DT.h + PREC.k15d + GDD15d + NDVI15d	3.715	0.006	0.687	0.70
57	Growth Rate ~ Intercept + DMI15d + GMP + DT.h + PREC + T.k15d + GDD15d	3.721	0.006	0.693	0.70
58	Growth Rate ~ Intercept + DMI15d + GMP + DT.h + PREC.k15d + T.night + GDD15d + NDVI15d	3.810	0.006	0.698	0.70
59	Growth Rate ~ Intercept + DMI15d + GMP + DT.h + GDD15d + NDVI15d	3.834	0.006	0.704	0.70
60	Growth Rate ~ Intercept + DMI15d + GMP + DT.h + PREC.k15d + T.k15d + GDD15d + NDVI15d	3.875	0.006	0.709	0.70
61	Growth Rate ~ Intercept + GMP + DT.h + PREC + T.k15d + GDD15d + NDVI15d	3.922	0.006	0.714	0.70
62	Growth Rate ~ Intercept + GMP + DT.h + PREC + T.k15d + T.sd15d + GDD15d	3.943	0.005	0.720	0.70
63	Growth Rate ~ Intercept + GMP + DT.h + T.sd15d + GDD15d + NDVI15d	3.967	0.005	0.725	0.70
64	Growth Rate ~ Intercept + GMP + DT.h + T.sd15d + T.night	4.056	0.005	0.730	0.70
65	Growth Rate ~ Intercept + GMP + DT.h + PREC + PREC.k15d + T.k15d + T.sd15d + GDD15d	4.275	0.005	0.734	0.70
66	Growth Rate ~ Intercept + GMP + DT.h + PREC.k15d	4.304	0.005	0.738	0.70
67	Growth Rate ~ Intercept + GMP + DT.h + PREC + T.sd15d + T.night + GDD15d	4.353	0.004	0.743	0.70
68	Growth Rate ~ Intercept + DMI15d + GMP + DT.h + PREC + PREC.k15d + T.k15d + T.night + GDD15d	4.355	0.004	0.747	0.71
69	Growth Rate ~ Intercept + GMP + DT.h + PREC + PREC.k15d + T.sd15d + GDD15d	4.357	0.004	0.751	0.70
70	Growth Rate ~ Intercept + GMP + DT.h + PREC.k15d + T.k15d + T.sd15d + GDD15d + NDVI15d	4.362	0.004	0.755	0.70
71	Growth Rate ~ Intercept + GMP + DT.h + PREC + PREC.k15d + T.k15d + GDD15d + NDVI15d	4.368	0.004	0.759	0.70
72	Growth Rate ~ Intercept + GMP + DT.h + PREC + T.night + GDD15d + NDVI15d	4.385	0.004	0.764	0.70
73	Growth Rate ~ Intercept + DMI15d + GMP + DT.h + PREC + T.k15d + T.night + GDD15d	4.413	0.004	0.768	0.70
74	Growth Rate ~ Intercept + GMP + DT.h + T.sd15d + T.night + GDD15d + NDVI15d	4.425	0.004	0.772	0.70

2011

Rank	Model	ΔAIC_c	$wAIC_c$	Cumulative $wAIC_c$	R_{LR}^2
75	Growth Rate ~ Intercept + DMI15d + GMP + DT.h + T.k15d + GDD15d + NDVI15d	4.435	0.004	0.776	0.70
76	Growth Rate ~ Intercept + DMI15d + GMP + DT.h + PREC.k15d + T.k15d + T.sd15d + T.night + GDD15d	4.436	0.004	0.780	0.71
77	Growth Rate ~ Intercept + DMI15d + GMP + DT.h + T.k15d + T.sd15d + GDD15d	4.454	0.004	0.784	0.70
78	Growth Rate ~ Intercept + GMP + DT.h + T.k15d + T.sd15d + GDD15d + NDVI15d	4.463	0.004	0.788	0.70
79	Growth Rate ~ Intercept + GMP + DT.h + PREC.k15d + T.sd15d + GDD15d + NDVI15d	4.466	0.004	0.792	0.70
80	Growth Rate ~ Intercept + DMI15d + GMP + DT.h + PREC.k15d + T.k15d + T.night + GDD15d + NDVI15d	4.479	0.004	0.796	0.71
81	Growth Rate ~ Intercept + DMI15d + GMP + DT.h + T.k15d + T.sd15d + T.night + GDD15d	4.641	0.004	0.800	0.70
82	Growth Rate ~ Intercept + DMI15d + GMP + DT.h + PREC + PREC.k15d + T.sd15d + GDD15d	4.675	0.004	0.803	0.70
83	Growth Rate ~ Intercept + DMI15d + GMP + DT.h + T.k15d + T.night + GDD15d + NDVI15d	4.685	0.004	0.807	0.70
84	Growth Rate ~ Intercept + GMP + DT.h + PREC + T.k15d + T.sd15d + T.night + GDD15d	4.802	0.004	0.810	0.70
85	Growth Rate ~ Intercept + GMP + DT.h + PREC + T.k15d + T.night + GDD15d + NDVI15d	4.836	0.003	0.813	0.70
86	Growth Rate ~ Intercept + GMP + DT.h + PREC + PREC.k15d + GDD15d + NDVI15d	4.906	0.003	0.817	0.70
87	Growth Rate ~ Intercept + GMP + DT.h + PREC.k15d + T.k15d + T.night	4.907	0.003	0.820	0.70
88	Growth Rate ~ Intercept + GMP + DT.h + T.k15d + T.sd15d + T.night + GDD15d + NDVI15d	4.923	0.003	0.823	0.70
89	Growth Rate ~ Intercept + DMI15d + GMP + DT.h + PREC + T.sd15d + GDD15d	4.927	0.003	0.826	0.70
90	Growth Rate ~ Intercept + GMP + DT.h + T.night + NDVI15d	4.976	0.003	0.829	0.70
91	Growth Rate ~ Intercept + DMI15d + GMP + DT.h + PREC + GDD15d + NDVI15d	5.018	0.003	0.832	0.70
92	Growth Rate ~ Intercept + GMP + DT.h + PREC.k15d + T.sd15d + T.night	5.070	0.003	0.835	0.70
93	Growth Rate ~ Intercept + DMI15d + GMP + DT.h + PREC.k15d + T.sd15d + GDD15d + NDVI15d	5.131	0.003	0.838	0.70
94	Growth Rate ~ Intercept + DMI15d + GMP + DT.h + PREC + PREC.k15d + GDD15d + NDVI15d	5.199	0.003	0.841	0.70
95	Growth Rate ~ Intercept + DMI15d + GMP + DT.h + PREC.k15d + T.night	5.228	0.003	0.844	0.70
96	Growth Rate ~ Intercept + GMP + DT.h + PREC.k15d + T.night + NDVI15d	5.248	0.003	0.846	0.70
97	Growth Rate ~ Intercept + GMP + DT.h + PREC + T.night	5.286	0.003	0.849	0.70
98	Growth Rate ~ Intercept + GMP + DT.h + T.k15d + T.night	5.296	0.003	0.852	0.70
99	Growth Rate ~ Intercept + DMI15d + GMP + DT.h + T.night	5.312	0.003	0.854	0.70
100	Growth Rate ~ Intercept + GMP + DT.h + T.k15d + T.sd15d + T.night	5.394	0.003	0.857	0.70
101	Growth Rate ~ Intercept + DMI15d + GMP + DT.h + PREC + T.night + GDD15d + NDVI15d	5.402	0.003	0.859	0.70
102	Growth Rate ~ Intercept + DMI15d + GMP + DT.h + PREC + T.sd15d + T.night + GDD15d	5.412	0.003	0.862	0.70
103	Growth Rate ~ Intercept + DMI15d + GMP + DT.h + PREC + PREC.k15d + T.k15d + T.sd15d + GDD15d	5.424	0.003	0.864	0.70
104	Growth Rate ~ Intercept + GMP + DT.h + PREC + PREC.k15d + T.night	5.467	0.003	0.867	0.70
105	Growth Rate ~ Intercept + GMP + DT.h + PREC.k15d + T.sd15d + T.night + GDD15d + NDVI15d	5.535	0.002	0.869	0.70
106	Growth Rate ~ Intercept + GMP + DT.h + PREC + PREC.k15d + T.sd15d + T.night + GDD15d	5.537	0.002	0.871	0.70
107	Growth Rate ~ Intercept + GMP + DT.h + PREC.k15d + T.k15d + T.sd15d + T.night + GDD15d + NDVI15d	5.539	0.002	0.874	0.70
108	Growth Rate ~ Intercept + DMI15d + GMP + DT.h + PREC + PREC.k15d + T.sd15d + T.night + GDD15d	5.540	0.002	0.876	0.70
109	Growth Rate ~ Intercept + GMP + DT.h + PREC + PREC.k15d + T.k15d + T.sd15d + T.night + GDD15d	5.540	0.002	0.878	0.70
110	Growth Rate ~ Intercept + GMP + DT.h + PREC + PREC.k15d + T.k15d + T.night + GDD15d + NDVI15d	5.554	0.002	0.881	0.70
111	Growth Rate ~ Intercept + DMI15d + GMP + DT.h + PREC + PREC.k15d + T.k15d + GDD15d + NDVI15d	5.571	0.002	0.883	0.70

2011

Rank	Model	ΔAIC$_c$	wAIC$_c$	Cumulative wAIC$_c$	R_{LR}^2
112	Growth Rate ~ Intercept + GMP + DT.h + PREC + T.sd15d + GDD15d + NDVI15d	5.624	0.002	0.885	0.70
113	Growth Rate ~ Intercept + GMP + DT.h	5.682	0.002	0.887	0.70
114	Growth Rate ~ Intercept + DMI15d + GMP + DT.h + PREC.k15d + T.sd15d + T.night + GDD15d + NDVI15d	5.690	0.002	0.890	0.70
115	Growth Rate ~ Intercept + DMI15d + GMP + DT.h + T.sd15d + T.night + GDD15d + NDVI15d	5.732	0.002	0.892	0.70
116	Growth Rate ~ Intercept + DMI15d + GMP + DT.h + PREC + PREC.k15d + T.night + GDD15d + NDVI15d	5.735	0.002	0.894	0.70
117	Growth Rate ~ Intercept + DMI15d + GMP + DT.h + PREC.k15d + T.k15d + T.sd15d + GDD15d + NDVI15d	5.758	0.002	0.896	0.70
118	Growth Rate ~ Intercept + DMI15d + GMP + DT.h + T.sd15d + GDD15d + NDVI15d	5.773	0.002	0.898	0.70
119	Growth Rate ~ Intercept + GMP + DT.h + PREC + PREC.k15d + T.night + GDD15d + NDVI15d	5.789	0.002	0.900	0.70
120	Growth Rate ~ Intercept + DMI15d + GMP + DT.h + PREC + T.k15d + GDD15d + NDVI15d	5.819	0.002	0.902	0.70
121	Growth Rate ~ Intercept + DMI15d + GMP + DT.h + PREC + T.k15d + T.sd15d + GDD15d	5.834	0.002	0.904	0.70
122	Growth Rate ~ Intercept + GMP + DT.h + PREC.k15d + T.k15d	5.857	0.002	0.906	0.70
123	Growth Rate ~ Intercept + GMP + DT.h + T.sd15d + T.night + NDVI15d	6.024	0.002	0.908	0.70
124	Growth Rate ~ Intercept + GMP + DT.h + PREC + T.k15d + T.sd15d + GDD15d + NDVI15d	6.035	0.002	0.910	0.70
125	Growth Rate ~ Intercept + GMP + DT.h + PREC.k15d + T.k15d + T.sd15d + T.night	6.061	0.002	0.912	0.70
126	Growth Rate ~ Intercept + GMP + DT.h + PREC + T.sd15d + T.night	6.066	0.002	0.913	0.70
127	Growth Rate ~ Intercept + DMI15d + GMP + DT.h + T.sd15d + T.night	6.110	0.002	0.915	0.70
128	Growth Rate ~ Intercept + GMP + DT.h + PREC + PREC.k15d	6.134	0.002	0.917	0.70
129	Growth Rate ~ Intercept + GMP + DT.h + PREC.k15d + T.sd15d	6.196	0.002	0.919	0.70
130	Growth Rate ~ Intercept + GMP + DT.h + PREC.k15d + NDVI15d	6.273	0.002	0.920	0.70
131	Growth Rate ~ Intercept + GMP + DT.h + PREC + PREC.k15d + T.k15d + T.sd15d + GDD15d + NDVI15d	6.367	0.002	0.922	0.70
132	Growth Rate ~ Intercept + DMI15d + GMP + DT.h + PREC.k15d	6.394	0.002	0.923	0.70
133	Growth Rate ~ Intercept + DMI15d + GMP + DT.h + PREC + PREC.k15d + T.k15d + T.sd15d + T.night + GDD15d	6.448	0.002	0.925	0.71
134	Growth Rate ~ Intercept + GMP + DT.h + PREC + T.sd15d + T.night + GDD15d + NDVI15d	6.458	0.002	0.926	0.70
135	Growth Rate ~ Intercept + GMP + DT.h + PREC + PREC.k15d + T.sd15d + GDD15d + NDVI15d	6.470	0.002	0.928	0.70
136	Growth Rate ~ Intercept + GMP + DT.h + T.sd15d	6.479	0.002	0.929	0.70
137	Growth Rate ~ Intercept + DMI15d + GMP + DT.h + PREC + PREC.k15d + T.k15d + T.night + GDD15d + NDVI15d	6.479	0.002	0.931	0.71
138	Growth Rate ~ Intercept + DMI15d + GMP + DT.h + PREC + T.k15d + T.sd15d + T.night + GDD15d	6.479	0.002	0.932	0.70
139	Growth Rate ~ Intercept + DMI15d + GMP + DT.h + PREC + T.k15d + T.night + GDD15d + NDVI15d	6.534	0.001	0.933	0.70
140	Growth Rate ~ Intercept + DMI15d + GMP + DT.h + T.k15d + T.sd15d + GDD15d + NDVI15d	6.544	0.001	0.935	0.70
141	Growth Rate ~ Intercept + DMI15d + GMP + DT.h + PREC.k15d + T.k15d + T.sd15d + T.night + GDD15d + NDVI15d	6.563	0.001	0.936	0.71
142	Growth Rate ~ Intercept + DMI15d + GMP + DT.h + PREC.k15d + T.sd15d + T.night	6.751	0.001	0.938	0.70
143	Growth Rate ~ Intercept + DMI15d + GMP + DT.h + T.k15d + T.sd15d + T.night + GDD15d + NDVI15d	6.762	0.001	0.939	0.70
144	Growth Rate ~ Intercept + GMP + DT.h + PREC.k15d + T.k15d + T.night + NDVI15d	6.794	0.001	0.940	0.70
145	Growth Rate ~ Intercept + DMI15d + GMP + DT.h + PREC + PREC.k15d + T.sd15d + GDD15d + NDVI15d	6.797	0.001	0.941	0.70
146	Growth Rate ~ Intercept + GMP + DT.h + PREC	6.808	0.001	0.943	0.70
147	Growth Rate ~ Intercept + DMI15d + GMP + DT.h	6.885	0.001	0.944	0.70
148	Growth Rate ~ Intercept + GMP + DT.h + PREC + T.k15d + T.sd15d + T.night + GDD15d + NDVI15d	6.920	0.001	0.945	0.70

2011

Rank	Model	ΔAIC$_c$	wAIC$_c$	Cumulative wAIC$_c$	R_{LR}^2
149	Growth Rate ~ Intercept + DMI15d + GMP + DT.h + PREC.k15d + T.k15d + T.night	6.961	0.001	0.946	0.70
150	Growth Rate ~ Intercept + GMP + DT.h + T.k15d + T.night + NDVI15d	6.990	0.001	0.947	0.70
151	Growth Rate ~ Intercept + GMP + DT.h + PREC + T.night + NDVI15d	6.993	0.001	0.948	0.70
152	Growth Rate ~ Intercept + GMP + DT.h + PREC + PREC.k15d + T.k15d + T.night	7.012	0.001	0.949	0.70

2012

Rank	Model	ΔAIC$_c$	wAIC$_c$	Cumulative wAIC$_c$	R_{LR}^2
1	Growth Rate ~ Intercept + DMI15d + GMP + DT.h + T.night + GDD15d	0.000	0.063	0.060	0.81
2	Growth Rate ~ Intercept + DMI15d + GMP + DT.h + PREC.k15d + T.night + GDD15d	0.296	0.054	0.111	0.81
3	Growth Rate ~ Intercept + DMI15d + GMP + DT.h + T.night	0.746	0.043	0.152	0.81
4	Growth Rate ~ Intercept + DMI15d + GMP + DT.h + PREC + T.night + GDD15d	1.476	0.030	0.181	0.81
5	Growth Rate ~ Intercept + DMI15d + GMP + DT.h + PREC + PREC.k15d + T.night + GDD15d	1.499	0.030	0.209	0.82
6	Growth Rate ~ Intercept + GMP + DT.h + T.night	1.667	0.027	0.235	0.81
7	Growth Rate ~ Intercept + GMP + DT.h + PREC.k15d + T.night	1.897	0.024	0.258	0.81
8	Growth Rate ~ Intercept + DMI15d + GMP + DT.h + PREC.k15d + T.night	1.905	0.024	0.281	0.81
9	Growth Rate ~ Intercept + DMI15d + GMP + DT.h + T.sd15d + T.night + GDD15d	1.918	0.024	0.304	0.81
10	Growth Rate ~ Intercept + DMI15d + GMP + DT.h + T.night + GDD15d + NDVI15d	2.084	0.022	0.325	0.81
11	Growth Rate ~ Intercept + DMI15d + GMP + DT.h + T.k15d + T.night + GDD15d	2.163	0.021	0.345	0.81
12	Growth Rate ~ Intercept + GMP + DT.h + PREC.k15d + T.night + GDD15d	2.190	0.021	0.365	0.81
13	Growth Rate ~ Intercept + DMI15d + GMP + DT.h + PREC.k15d + T.night + GDD15d + NDVI15d	2.331	0.020	0.384	0.81
14	Growth Rate ~ Intercept + DMI15d + GMP + DT.h + PREC.k15d + T.sd15d + T.night + GDD15d	2.367	0.019	0.402	0.81
15	Growth Rate ~ Intercept + DMI15d + GMP + DT.h + PREC + T.night	2.370	0.019	0.420	0.81
16	Growth Rate ~ Intercept + DMI15d + GMP + DT.h + PREC.k15d + T.k15d + T.night + GDD15d	2.504	0.018	0.437	0.81
17	Growth Rate ~ Intercept + DMI15d + GMP + DT.h + T.sd15d + T.night	2.867	0.015	0.452	0.81
18	Growth Rate ~ Intercept + DMI15d + GMP + DT.h + T.k15d + T.night	2.882	0.015	0.466	0.81
19	Growth Rate ~ Intercept + GMP + DT.h + T.night + GDD15d	2.896	0.015	0.480	0.81
20	Growth Rate ~ Intercept + DMI15d + GMP + DT.h + T.night + NDVI15d	2.910	0.015	0.494	0.81
21	Growth Rate ~ Intercept + DMI15d + GMP + DT.h + PREC + PREC.k15d + T.night	3.373	0.012	0.505	0.81
22	Growth Rate ~ Intercept + DMI15d + GMP + DT.h + PREC + T.sd15d + T.night + GDD15d	3.451	0.011	0.515	0.81
23	Growth Rate ~ Intercept + DMI15d + GMP + DT.h + PREC + PREC.k15d + T.night + GDD15d + NDVI15d	3.533	0.011	0.526	0.82
24	Growth Rate ~ Intercept + DMI15d + GMP + DT.h + PREC + T.k15d + T.night + GDD15d	3.556	0.011	0.536	0.81
25	Growth Rate ~ Intercept + DMI15d + GMP + DT.h + PREC + T.night + GDD15d + NDVI15d	3.569	0.011	0.546	0.81
26	Growth Rate ~ Intercept + DMI15d + GMP + DT.h + PREC + PREC.k15d + T.sd15d + T.night + GDD15d	3.628	0.010	0.555	0.82
27	Growth Rate ~ Intercept + DMI15d + GMP + DT.h + PREC + PREC.k15d + T.k15d + T.night + GDD15d	3.674	0.010	0.565	0.82
28	Growth Rate ~ Intercept + GMP + DT.h + PREC + T.night	3.754	0.010	0.574	0.81
29	Growth Rate ~ Intercept + GMP + DT.h + T.k15d + T.night	3.764	0.010	0.583	0.81
30	Growth Rate ~ Intercept + GMP + DT.h + T.sd15d + T.night	3.790	0.009	0.592	0.81
31	Growth Rate ~ Intercept + GMP + DT.h + T.night + NDVI15d	3.829	0.009	0.601	0.81
32	Growth Rate ~ Intercept + DMI15d + GMP + DT.h	3.933	0.009	0.609	0.81
33	Growth Rate ~ Intercept + DMI15d + GMP + DT.h + T.sd15d + T.night + GDD15d + NDVI15d	3.970	0.009	0.617	0.81
34	Growth Rate ~ Intercept + GMP + DT.h + PREC.k15d + T.k15d + T.night	4.063	0.008	0.625	0.81
35	Growth Rate ~ Intercept + GMP + DT.h + PREC.k15d + T.night + NDVI15d	4.075	0.008	0.633	0.81
36	Growth Rate ~ Intercept + GMP + DT.h + PREC.k15d + T.sd15d + T.night	4.075	0.008	0.641	0.81
37	Growth Rate ~ Intercept + GMP + DT.h + PREC + PREC.k15d + T.night	4.076	0.008	0.649	0.81
38	Growth Rate ~ Intercept + DMI15d + GMP + DT.h + PREC.k15d + T.night + NDVI15d	4.088	0.008	0.656	0.81

2012

Rank	Model	ΔAIC$_c$	wAIC$_c$	Cumulative wAIC$_c$	R_{LR}^2
39	Growth Rate ~ Intercept + DMI15d + GMP + DT.h + PREC.k15d + T.k15d + T.night	4.088	0.008	0.664	0.81
40	Growth Rate ~ Intercept + DMI15d + GMP + DT.h + PREC.k15d + T.sd15d + T.night	4.088	0.008	0.672	0.81
41	Growth Rate ~ Intercept + DMI15d + GMP + DT.h + T.k15d + T.sd15d + T.night + GDD15d	4.090	0.008	0.680	0.81
42	Growth Rate ~ Intercept + DMI15d + GMP + DT.h + T.k15d + T.night + GDD15d + NDVI15d	4.266	0.007	0.687	0.81
43	Growth Rate ~ Intercept + GMP + DT.h + PREC.k15d + T.night + GDD15d + NDVI15d	4.278	0.007	0.694	0.81
44	Growth Rate ~ Intercept + GMP + DT.h + PREC.k15d + T.sd15d + T.night + GDD15d	4.353	0.007	0.700	0.81
45	Growth Rate ~ Intercept + GMP + DT.h + PREC + PREC.k15d + T.night + GDD15d	4.365	0.007	0.707	0.81
46	Growth Rate ~ Intercept + DMI15d + GMP + DT.h + PREC.k15d + T.sd15d + T.night + GDD15d + NDVI15d	4.379	0.007	0.714	0.81
47	Growth Rate ~ Intercept + GMP + DT.h + PREC.k15d + T.k15d + T.night + GDD15d	4.381	0.007	0.720	0.81
48	Growth Rate ~ Intercept + DMI15d + GMP + DT.h + PREC + T.k15d + T.night	4.422	0.007	0.727	0.81
49	Growth Rate ~ Intercept + DMI15d + GMP + DT.h + PREC + T.sd15d + T.night	4.526	0.007	0.733	0.81
50	Growth Rate ~ Intercept + DMI15d + GMP + DT.h + PREC + T.night + NDVI15d	4.549	0.006	0.739	0.81
51	Growth Rate ~ Intercept + DMI15d + GMP + DT.h + PREC.k15d + T.k15d + T.night + GDD15d + NDVI15d	4.555	0.006	0.745	0.81
52	Growth Rate ~ Intercept + DMI15d + GMP + DT.h + PREC.k15d + T.k15d + T.sd15d + T.night + GDD15d	4.590	0.006	0.751	0.81
53	Growth Rate ~ Intercept + GMP + DT.h + PREC + T.night + GDD15d	4.888	0.005	0.757	0.81
54	Growth Rate ~ Intercept + GMP + DT.h + T.sd15d + T.night + GDD15d	4.956	0.005	0.762	0.81
55	Growth Rate ~ Intercept + DMI15d + GMP + DT.h + T.k15d + T.sd15d + T.night	5.014	0.005	0.767	0.81
56	Growth Rate ~ Intercept + GMP + DT.h + T.k15d + T.night + GDD15d	5.016	0.005	0.771	0.81
57	Growth Rate ~ Intercept + GMP + DT.h + T.night + GDD15d + NDVI15d	5.036	0.005	0.776	0.81
58	Growth Rate ~ Intercept + DMI15d + GMP + DT.h + T.sd15d + T.night + NDVI15d	5.050	0.005	0.781	0.81
59	Growth Rate ~ Intercept + DMI15d + GMP + DT.h + T.k15d + T.night + NDVI15d	5.060	0.005	0.786	0.81
60	Growth Rate ~ Intercept + DMI15d + GMP + DT.h + PREC.k15d	5.378	0.004	0.790	0.81
61	Growth Rate ~ Intercept + DMI15d + GMP + DT.h + PREC + PREC.k15d + T.k15d + T.night	5.498	0.004	0.794	0.81
62	Growth Rate ~ Intercept + DMI15d + GMP + DT.h + PREC + T.sd15d + T.night + GDD15d + NDVI15d	5.514	0.004	0.797	0.81
63	Growth Rate ~ Intercept + DMI15d + GMP + DT.h + PREC + T.k15d + T.sd15d + T.night + GDD15d	5.537	0.004	0.801	0.81
64	Growth Rate ~ Intercept + DMI15d + GMP + DT.h + GDD15d	5.549	0.004	0.805	0.81
65	Growth Rate ~ Intercept + DMI15d + GMP + DT.h + PREC + PREC.k15d + T.night + NDVI15d	5.571	0.004	0.809	0.81
66	Growth Rate ~ Intercept + DMI15d + GMP + DT.h + PREC + PREC.k15d + T.sd15d + T.night	5.580	0.004	0.812	0.81
67	Growth Rate ~ Intercept + DMI15d + GMP + DT.h + PREC + PREC.k15d + T.sd15d + T.night + GDD15d + NDVI15d	5.643	0.004	0.816	0.82
68	Growth Rate ~ Intercept + DMI15d + GMP + DT.h + PREC + T.k15d + T.night + GDD15d + NDVI15d	5.671	0.004	0.819	0.81
69	Growth Rate ~ Intercept + DMI15d + GMP + DT.h + PREC + PREC.k15d + T.k15d + T.night + GDD15d + NDVI15d	5.729	0.004	0.823	0.82
70	Growth Rate ~ Intercept + DMI15d + GMP + DT.h + T.k15d	5.749	0.004	0.826	0.81
71	Growth Rate ~ Intercept + DMI15d + GMP + DT.h + PREC	5.787	0.003	0.829	0.81
72	Growth Rate ~ Intercept + DMI15d + GMP + DT.h + PREC + PREC.k15d + T.k15d + T.sd15d + T.night + GDD15d	5.812	0.003	0.833	0.82
73	Growth Rate ~ Intercept + GMP + DT.h + PREC + T.sd15d + T.night	5.884	0.003	0.836	0.81
74	Growth Rate ~ Intercept + GMP + DT.h + PREC + T.k15d + T.night	5.889	0.003	0.839	0.81
75	Growth Rate ~ Intercept + GMP + DT.h + T.k15d + T.sd15d + T.night	5.898	0.003	0.842	0.81
76	Growth Rate ~ Intercept + GMP + DT.h + PREC + T.night + NDVI15d	5.930	0.003	0.845	0.81

2012

Rank	Model	ΔAIC_c	$wAIC_c$	Cumulative $wAIC_c$	R_{LR}^2
77	Growth Rate ~ Intercept + GMP + DT.h + T.k15d + T.night + NDVI15d	5.940	0.003	0.848	0.81
78	Growth Rate ~ Intercept + GMP + DT.h + T.sd15d + T.night + NDVI15d	5.968	0.003	0.851	0.81
79	Growth Rate ~ Intercept + DMI15d + GMP + DT.h + T.sd15d	6.087	0.003	0.854	0.81
80	Growth Rate ~ Intercept + DMI15d + GMP + DT.h + NDVI15d	6.097	0.003	0.857	0.81
81	Growth Rate ~ Intercept + DMI15d + GMP + DT.h + T.k15d + T.sd15d + T.night + GDD15d + NDVI15d	6.161	0.003	0.860	0.81
82	Growth Rate ~ Intercept + GMP + DT.h	6.177	0.003	0.862	0.81
83	Growth Rate ~ Intercept + GMP + DT.h + PREC.k15d + T.k15d + T.night + NDVI15d	6.255	0.003	0.865	0.81
84	Growth Rate ~ Intercept + GMP + DT.h + PREC + PREC.k15d + T.k15d + T.night	6.256	0.003	0.868	0.81
85	Growth Rate ~ Intercept + GMP + DT.h + PREC.k15d + T.k15d + T.sd15d + T.night	6.256	0.003	0.870	0.81
86	Growth Rate ~ Intercept + GMP + DT.h + PREC.k15d + T.sd15d + T.night + NDVI15d	6.268	0.003	0.873	0.81
87	Growth Rate ~ Intercept + GMP + DT.h + PREC + PREC.k15d + T.night + NDVI15d	6.268	0.003	0.875	0.81
88	Growth Rate ~ Intercept + GMP + DT.h + PREC + PREC.k15d + T.sd15d + T.night	6.269	0.003	0.878	0.81
89	Growth Rate ~ Intercept + DMI15d + GMP + DT.h + PREC.k15d + T.k15d + T.sd15d + T.night	6.285	0.003	0.881	0.81
90	Growth Rate ~ Intercept + DMI15d + GMP + DT.h + PREC.k15d + T.k15d + T.night + NDVI15d	6.286	0.003	0.883	0.81
91	Growth Rate ~ Intercept + DMI15d + GMP + DT.h + PREC.k15d + T.sd15d + T.night + NDVI15d	6.288	0.003	0.886	0.81
92	Growth Rate ~ Intercept + GMP + DT.h + PREC.k15d + T.sd15d + T.night + GDD15d + NDVI15d	6.442	0.003	0.888	0.81
93	Growth Rate ~ Intercept + GMP + DT.h + PREC + PREC.k15d + T.night + GDD15d + NDVI15d	6.468	0.002	0.890	0.81
94	Growth Rate ~ Intercept + GMP + DT.h + PREC.k15d + T.k15d + T.night + GDD15d + NDVI15d	6.485	0.002	0.893	0.81
95	Growth Rate ~ Intercept + GMP + DT.h + PREC + PREC.k15d + T.sd15d + T.night + GDD15d	6.538	0.002	0.895	0.81
96	Growth Rate ~ Intercept + GMP + DT.h + PREC.k15d + T.k15d + T.sd15d + T.night + GDD15d	6.559	0.002	0.897	0.81
97	Growth Rate ~ Intercept + GMP + DT.h + PREC + PREC.k15d + T.k15d + T.night + GDD15d	6.574	0.002	0.899	0.81
98	Growth Rate ~ Intercept + DMI15d + GMP + DT.h + PREC + T.k15d + T.sd15d + T.night	6.589	0.002	0.902	0.81
99	Growth Rate ~ Intercept + GMP + DT.h + PREC.k15d	6.591	0.002	0.904	0.81
100	Growth Rate ~ Intercept + DMI15d + GMP + DT.h + PREC + T.k15d + T.night + NDVI15d	6.614	0.002	0.906	0.81
101	Growth Rate ~ Intercept + DMI15d + GMP + DT.h + PREC.k15d + T.k15d + T.sd15d + T.night + GDD15d + NDVI15d	6.618	0.002	0.908	0.81
102	Growth Rate ~ Intercept + DMI15d + GMP + DT.h + PREC + T.sd15d + T.night + NDVI15d	6.723	0.002	0.910	0.81
103	Growth Rate ~ Intercept + DMI15d + GMP + DT.h + PREC.k15d + GDD15d	6.765	0.002	0.912	0.81
104	Growth Rate ~ Intercept + GMP + DT.h + PREC + T.sd15d + T.night + GDD15d	6.937	0.002	0.914	0.81
105	Growth Rate ~ Intercept + GMP + DT.h + PREC + T.night + GDD15d + NDVI15d	7.039	0.002	0.916	0.81
106	Growth Rate ~ Intercept + GMP + DT.h + PREC + T.k15d + T.night + GDD15d	7.055	0.002	0.918	0.81
107	Growth Rate ~ Intercept + DMI15d + GMP + DT.h + PREC.k15d + T.k15d	7.066	0.002	0.920	0.81
108	Growth Rate ~ Intercept + GMP + DT.h + T.k15d + T.sd15d + T.night + GDD15d	7.084	0.002	0.921	0.81
109	Growth Rate ~ Intercept + GMP + DT.h + T.sd15d + T.night + GDD15d + NDVI15d	7.092	0.002	0.923	0.81
110	Growth Rate ~ Intercept + DMI15d + GMP + DT.h + PREC + PREC.k15d	7.160	0.002	0.925	0.81
111	Growth Rate ~ Intercept + GMP + DT.h + T.k15d + T.night + GDD15d + NDVI15d	7.172	0.002	0.926	0.81
112	Growth Rate ~ Intercept + DMI15d + GMP + DT.h + T.k15d + T.sd15d + T.night + NDVI15d	7.211	0.002	0.928	0.81
113	Growth Rate ~ Intercept + DMI15d + GMP + DT.h + T.k15d + GDD15d	7.227	0.002	0.929	0.81
114	Growth Rate ~ Intercept + DMI15d + GMP + DT.h + PREC + GDD15d	7.394	0.002	0.931	0.81

2012

Rank	Model	ΔAIC$_c$	wAIC$_c$	Cumulative wAIC$_c$	R_{LR}^2
115	Growth Rate ~ Intercept + DMI15d + GMP + DT.h + PREC.k15d + T.sd15d	7.557	0.001	0.932	0.81
116	Growth Rate ~ Intercept + DMI15d + GMP + DT.h + PREC.k15d + NDVI15d	7.557	0.001	0.934	0.81
117	Growth Rate ~ Intercept + DMI15d + GMP + DT.h + PREC + T.k15d + T.sd15d + T.night + GDD15d + NDVI15d	7.623	0.001	0.935	0.81
118	Growth Rate ~ Intercept + DMI15d + GMP + DT.h + T.sd15d + GDD15d	7.690	0.001	0.936	0.81
119	Growth Rate ~ Intercept + DMI15d + GMP + DT.h + GDD15d + NDVI15d	7.704	0.001	0.938	0.81
120	Growth Rate ~ Intercept + DMI15d + GMP + DT.h + PREC + PREC.k15d + T.k15d + T.night + NDVI15d	7.711	0.001	0.939	0.81
121	Growth Rate ~ Intercept + DMI15d + GMP + DT.h + PREC + T.k15d	7.718	0.001	0.940	0.81
122	Growth Rate ~ Intercept + DMI15d + GMP + DT.h + PREC + PREC.k15d + T.k15d + T.sd15d + T.night	7.720	0.001	0.941	0.81
123	Growth Rate ~ Intercept + DMI15d + GMP + DT.h + PREC + PREC.k15d + T.sd15d + T.night + NDVI15d	7.795	0.001	0.943	0.81
124	Growth Rate ~ Intercept + DMI15d + GMP + DT.h + PREC + PREC.k15d + T.k15d + T.sd15d + T.night + GDD15d + NDVI15d	7.849	0.001	0.944	0.82
125	Growth Rate ~ Intercept + DMI15d + GMP + DT.h + T.k15d + T.sd15d	7.919	0.001	0.945	0.81
126	Growth Rate ~ Intercept + GMP + DT.h + T.k15d	7.926	0.001	0.946	0.81
127	Growth Rate ~ Intercept + DMI15d + GMP + DT.h + T.k15d + NDVI15d	7.927	0.001	0.947	0.81
128	Growth Rate ~ Intercept + GMP + DT.h + PREC	7.932	0.001	0.948	0.81
129	Growth Rate ~ Intercept + DMI15d + GMP + DT.h + PREC + T.sd15d	7.962	0.001	0.949	0.81

2013

Rank	Model	ΔAIC_c	$wAIC_c$	Cumulative $wAIC_c$	R_{LR}^2
1	Growth Rate ~ Intercept + DMI15d + GMP + DT.h + PREC.k15d + T.k15d + T.sd15d + NDVI15d	0.000	0.050	0.048	0.71
2	Growth Rate ~ Intercept + DMI15d + GMP + DT.h + T.k15d + T.sd15d + NDVI15d	0.083	0.048	0.093	0.70
3	Growth Rate ~ Intercept + DMI15d + GMP + DT.h + T.k15d + NDVI15d	0.142	0.047	0.137	0.70
4	Growth Rate ~ Intercept + DMI15d + GMP + DT.h + PREC.k15d + T.k15d + NDVI15d	0.249	0.044	0.179	0.70
5	Growth Rate ~ Intercept + DMI15d + GMP + DT.h + PREC.k15d + NDVI15d	0.316	0.043	0.220	0.70
6	Growth Rate ~ Intercept + DMI15d + GMP + DT.h + NDVI15d	0.351	0.042	0.260	0.70
7	Growth Rate ~ Intercept + DMI15d + GMP + DT.h + PREC + PREC.k15d + T.k15d + NDVI15d	0.997	0.030	0.289	0.70
8	Growth Rate ~ Intercept + DMI15d + GMP + DT.h + PREC + PREC.k15d + NDVI15d	1.033	0.030	0.317	0.70
9	Growth Rate ~ Intercept + DMI15d + GMP + DT.h + PREC + PREC.k15d + T.k15d + T.sd15d + NDVI15d	1.134	0.028	0.344	0.71
10	Growth Rate ~ Intercept + DMI15d + GMP + DT.h + PREC.k15d + T.sd15d + NDVI15d	1.143	0.028	0.371	0.70
11	Growth Rate ~ Intercept + DMI15d + GMP + DT.h + PREC + T.k15d + NDVI15d	1.295	0.026	0.396	0.70
12	Growth Rate ~ Intercept + DMI15d + GMP + DT.h + T.sd15d + NDVI15d	1.338	0.026	0.420	0.70
13	Growth Rate ~ Intercept + DMI15d + GMP + DT.h + PREC + NDVI15d	1.484	0.024	0.443	0.70
14	Growth Rate ~ Intercept + DMI15d + GMP + DT.h + PREC + T.k15d + T.sd15d + NDVI15d	1.547	0.023	0.465	0.70
15	Growth Rate ~ Intercept + DMI15d + GMP + DT.h + PREC.k15d + T.k15d + T.sd15d + GDD15d + NDVI15d	1.853	0.020	0.483	0.71
16	Growth Rate ~ Intercept + DMI15d + GMP + DT.h + PREC.k15d + T.k15d + T.sd15d + T.night + NDVI15d	1.986	0.019	0.501	0.71
17	Growth Rate ~ Intercept + DMI15d + GMP + DT.h + T.k15d + T.night + NDVI15d	2.106	0.017	0.518	0.70
18	Growth Rate ~ Intercept + DMI15d + GMP + DT.h + T.k15d + GDD15d + NDVI15d	2.126	0.017	0.534	0.70
19	Growth Rate ~ Intercept + DMI15d + GMP + DT.h + PREC + PREC.k15d + T.sd15d + NDVI15d	2.141	0.017	0.550	0.70
20	Growth Rate ~ Intercept + DMI15d + GMP + DT.h + T.k15d + T.sd15d + T.night + NDVI15d	2.167	0.017	0.566	0.70
21	Growth Rate ~ Intercept + DMI15d + GMP + DT.h + T.k15d + T.sd15d + GDD15d + NDVI15d	2.167	0.017	0.583	0.70
22	Growth Rate ~ Intercept + DMI15d + GMP + DT.h + PREC.k15d + GDD15d + NDVI15d	2.289	0.016	0.598	0.70
23	Growth Rate ~ Intercept + DMI15d + GMP + DT.h + PREC.k15d + T.k15d + GDD15d + NDVI15d	2.315	0.016	0.613	0.70
24	Growth Rate ~ Intercept + DMI15d + GMP + DT.h + PREC.k15d + T.k15d + T.night + NDVI15d	2.332	0.016	0.627	0.70
25	Growth Rate ~ Intercept + DMI15d + GMP + DT.h + T.night + NDVI15d	2.351	0.015	0.642	0.70
26	Growth Rate ~ Intercept + DMI15d + GMP + DT.h + PREC.k15d + T.night + NDVI15d	2.393	0.015	0.656	0.70
27	Growth Rate ~ Intercept + DMI15d + GMP + DT.h + GDD15d + NDVI15d	2.406	0.015	0.671	0.70
28	Growth Rate ~ Intercept + DMI15d + GMP + DT.h + PREC + T.sd15d + NDVI15d	2.694	0.013	0.683	0.70
29	Growth Rate ~ Intercept + DMI15d + GMP + DT.h + PREC.k15d + T.sd15d + GDD15d + NDVI15d	2.848	0.012	0.695	0.70
30	Growth Rate ~ Intercept + DMI15d + GMP + DT.h + PREC + PREC.k15d + GDD15d + NDVI15d	3.022	0.011	0.705	0.70
31	Growth Rate ~ Intercept + DMI15d + GMP + DT.h + PREC + PREC.k15d + T.k15d + T.sd15d + GDD15d + NDVI15d	3.029	0.011	0.715	0.71
32	Growth Rate ~ Intercept + DMI15d + GMP + DT.h + PREC + PREC.k15d + T.k15d + T.night + NDVI15d	3.072	0.011	0.726	0.70
33	Growth Rate ~ Intercept + DMI15d + GMP + DT.h + PREC + PREC.k15d + T.k15d + GDD15d + NDVI15d	3.073	0.011	0.736	0.70
34	Growth Rate ~ Intercept + DMI15d + GMP + DT.h + PREC + PREC.k15d + T.night + NDVI15d	3.115	0.011	0.746	0.70
35	Growth Rate ~ Intercept + DMI15d + GMP + DT.h + PREC.k15d + T.sd15d + T.night + NDVI15d	3.129	0.010	0.756	0.70
36	Growth Rate ~ Intercept + DMI15d + GMP + DT.h + PREC + PREC.k15d + T.k15d + T.sd15d + T.night + NDVI15d	3.183	0.010	0.766	0.71
37	Growth Rate ~ Intercept + DMI15d + GMP + DT.h + PREC + T.k15d + T.night + NDVI15d	3.186	0.010	0.775	0.70
38	Growth Rate ~ Intercept + DMI15d + GMP + DT.h + PREC + T.k15d + GDD15d + NDVI15d	3.247	0.010	0.785	0.70

2013

Rank	Model	ΔAIC$_c$	wAIC$_c$	Cumulative wAIC$_c$	R_{LR}^2
39	Growth Rate ~ Intercept + DMI15d + GMP + DT.h + T.sd15d + GDD15d + NDVI15d	3.408	0.009	0.793	0.70
40	Growth Rate ~ Intercept + DMI15d + GMP + DT.h + T.sd15d + T.night + NDVI15d	3.415	0.009	0.802	0.70
41	Growth Rate ~ Intercept + DMI15d + GMP + DT.h + PREC + T.night + NDVI15d	3.425	0.009	0.810	0.70
42	Growth Rate ~ Intercept + DMI15d + GMP + DT.h + PREC + GDD15d + NDVI15d	3.526	0.009	0.819	0.70
43	Growth Rate ~ Intercept + DMI15d + GMP + DT.h + PREC + T.k15d + T.sd15d + T.night + NDVI15d	3.620	0.008	0.826	0.70
44	Growth Rate ~ Intercept + DMI15d + GMP + DT.h + PREC + T.k15d + T.sd15d + GDD15d + NDVI15d	3.630	0.008	0.834	0.70
45	Growth Rate ~ Intercept + DMI15d + GMP + DT.h + PREC.k15d + T.k15d + T.sd15d + T.night + GDD15d + NDVI15d	3.753	0.008	0.841	0.71
46	Growth Rate ~ Intercept + DMI15d + GMP + DT.h + PREC + PREC.k15d + T.sd15d + GDD15d + NDVI15d	3.903	0.007	0.848	0.70
47	Growth Rate ~ Intercept + DMI15d + GMP + DT.h + T.k15d + T.night + GDD15d + NDVI15d	4.077	0.007	0.854	0.70
48	Growth Rate ~ Intercept + DMI15d + GMP + DT.h + PREC + PREC.k15d + T.sd15d + T.night + NDVI15d	4.191	0.006	0.860	0.70
49	Growth Rate ~ Intercept + DMI15d + GMP + DT.h + T.k15d + T.sd15d + T.night + GDD15d + NDVI15d	4.256	0.006	0.866	0.70
50	Growth Rate ~ Intercept + DMI15d + GMP + DT.h + PREC.k15d + T.night + GDD15d + NDVI15d	4.365	0.006	0.871	0.70
51	Growth Rate ~ Intercept + DMI15d + GMP + DT.h + PREC.k15d + T.k15d + T.night + GDD15d + NDVI15d	4.405	0.006	0.876	0.70
52	Growth Rate ~ Intercept + DMI15d + GMP + DT.h + T.night + GDD15d + NDVI15d	4.405	0.006	0.882	0.70
53	Growth Rate ~ Intercept + DMI15d + GMP + DT.h + PREC.k15d + T.sd15d + T.night + GDD15d + NDVI15d	4.721	0.005	0.886	0.70
54	Growth Rate ~ Intercept + DMI15d + GMP + DT.h + PREC + T.sd15d + T.night + NDVI15d	4.754	0.005	0.891	0.70
55	Growth Rate ~ Intercept + DMI15d + GMP + DT.h + PREC + T.sd15d + GDD15d + NDVI15d	4.778	0.005	0.895	0.70
56	Growth Rate ~ Intercept + DMI15d + GMP + DT.h + PREC + PREC.k15d + T.k15d + T.sd15d + T.night + GDD15d + NDVI15d	5.023	0.004	0.899	0.71
57	Growth Rate ~ Intercept + DMI15d + GMP + DT.h + PREC + PREC.k15d + T.night + GDD15d + NDVI15d	5.113	0.004	0.902	0.70
58	Growth Rate ~ Intercept + DMI15d + GMP + DT.h + PREC + T.k15d + T.night + GDD15d + NDVI15d	5.114	0.004	0.906	0.70
59	Growth Rate ~ Intercept + DMI15d + GMP + DT.h + PREC + PREC.k15d + T.k15d + T.night + GDD15d + NDVI15d	5.158	0.004	0.910	0.70
60	Growth Rate ~ Intercept + DMI15d + GMP + DT.h + PREC + T.night + GDD15d + NDVI15d	5.461	0.003	0.913	0.70
61	Growth Rate ~ Intercept + DMI15d + GMP + DT.h + T.k15d + T.sd15d	5.487	0.003	0.916	0.70
62	Growth Rate ~ Intercept + DMI15d + GMP + DT.h + T.sd15d + T.night + GDD15d + NDVI15d	5.492	0.003	0.919	0.70
63	Growth Rate ~ Intercept + DMI15d + GMP + DT.h + PREC.k15d + T.k15d + T.sd15d	5.516	0.003	0.922	0.70
64	Growth Rate ~ Intercept + DMI15d + GMP + DT.h + PREC + T.k15d + T.sd15d + T.night + GDD15d + NDVI15d	5.705	0.003	0.925	0.70
65	Growth Rate ~ Intercept + DMI15d + GMP + DT.h + PREC + PREC.k15d + T.sd15d + T.night + GDD15d + NDVI15d	5.881	0.003	0.927	0.70
66	Growth Rate ~ Intercept + GMP + DT.h + PREC.k15d + T.sd15d + GDD15d + NDVI15d	6.133	0.002	0.929	0.70
67	Growth Rate ~ Intercept + GMP + DT.h + PREC.k15d + T.k15d + T.sd15d + GDD15d + NDVI15d	6.357	0.002	0.931	0.70
68	Growth Rate ~ Intercept + DMI15d + GMP + DT.h + PREC + PREC.k15d + T.k15d + T.sd15d	6.462	0.002	0.933	0.70
69	Growth Rate ~ Intercept + DMI15d + GMP + DT.h + PREC.k15d + T.k15d + T.sd15d + GDD15d	6.622	0.002	0.935	0.70
70	Growth Rate ~ Intercept + DMI15d + GMP + DT.h + PREC.k15d + T.sd15d	6.622	0.002	0.937	0.70
71	Growth Rate ~ Intercept + DMI15d + GMP + DT.h + T.sd15d	6.683	0.002	0.938	0.70
72	Growth Rate ~ Intercept + DMI15d + GMP + DT.h + PREC + T.k15d + T.sd15d	6.792	0.002	0.940	0.70
73	Growth Rate ~ Intercept + DMI15d + GMP + DT.h + PREC + T.sd15d + T.night + GDD15d + NDVI15d	6.844	0.002	0.942	0.70
74	Growth Rate ~ Intercept + GMP + DT.h + T.sd15d + GDD15d + NDVI15d	7.140	0.001	0.943	0.70
75	Growth Rate ~ Intercept + GMP + DT.h + T.k15d + T.sd15d + GDD15d + NDVI15d	7.194	0.001	0.944	0.70
76	Growth Rate ~ Intercept + DMI15d + GMP + DT.h + T.k15d + T.sd15d + GDD15d	7.346	0.001	0.945	0.70

2013

Rank	Model	ΔAIC_c	$wAIC_c$	Cumulative $wAIC_c$	R_{LR}^2
77	Growth Rate ~ Intercept + GMP + DT.h + PREC + PREC.k15d + T.sd15d + GDD15d + NDVI15d	7.373	0.001	0.947	0.70
78	Growth Rate ~ Intercept + DMI15d + GMP + DT.h	7.407	0.001	0.948	0.70
79	Growth Rate ~ Intercept + DMI15d + GMP + DT.h + PREC + PREC.k15d + T.sd15d	7.423	0.001	0.949	0.70

2014

Rank	Model	ΔAIC$_c$	wAIC$_c$	Cumulative wAIC$_c$	R_{LR}^2
1	Growth Rate ~ Intercept + GMP + DT.h + T.sd15d + GDD15d + NDVI15d	0.000	0.076	0.072	0.81
2	Growth Rate ~ Intercept + GMP + DT.h + T.k15d + T.sd15d + GDD15d + NDVI15d	0.005	0.076	0.144	0.81
3	Growth Rate ~ Intercept + GMP + DT.h + T.sd15d + T.night + GDD15d + NDVI15d	0.818	0.050	0.191	0.81
4	Growth Rate ~ Intercept + DMI15d + GMP + DT.h + T.sd15d + GDD15d + NDVI15d	0.909	0.048	0.237	0.81
5	Growth Rate ~ Intercept + DMI15d + GMP + DT.h + T.k15d + T.sd15d + GDD15d + NDVI15d	0.919	0.048	0.282	0.81
6	Growth Rate ~ Intercept + GMP + DT.h + T.k15d + T.sd15d + T.night + GDD15d + NDVI15d	1.084	0.044	0.324	0.81
7	Growth Rate ~ Intercept + DMI15d + GMP + DT.h + T.sd15d + T.night + GDD15d + NDVI15d	1.585	0.034	0.357	0.81
8	Growth Rate ~ Intercept + GMP + DT.h + PREC.k15d + T.sd15d + GDD15d + NDVI15d	1.698	0.032	0.388	0.81
9	Growth Rate ~ Intercept + GMP + DT.h + PREC.k15d + T.k15d + T.sd15d + GDD15d + NDVI15d	1.740	0.032	0.418	0.81
10	Growth Rate ~ Intercept + DMI15d + GMP + DT.h + T.k15d + T.sd15d + T.night + GDD15d + NDVI15d	1.868	0.030	0.446	0.81
11	Growth Rate ~ Intercept + DMI15d + GMP + DT.h + PREC.k15d + T.sd15d + GDD15d + NDVI15d	2.090	0.027	0.471	0.81
12	Growth Rate ~ Intercept + DMI15d + GMP + DT.h + PREC.k15d + T.k15d + T.sd15d + GDD15d + NDVI15d	2.144	0.026	0.496	0.81
13	Growth Rate ~ Intercept + GMP + DT.h + PREC + T.k15d + T.sd15d + GDD15d + NDVI15d	2.145	0.026	0.520	0.81
14	Growth Rate ~ Intercept + GMP + DT.h + PREC + T.sd15d + GDD15d + NDVI15d	2.149	0.026	0.545	0.81
15	Growth Rate ~ Intercept + GMP + DT.h + PREC.k15d + T.sd15d + T.night + GDD15d + NDVI15d	2.717	0.019	0.563	0.81
16	Growth Rate ~ Intercept + GMP + DT.h + PREC + T.sd15d + T.night + GDD15d + NDVI15d	2.944	0.017	0.580	0.81
17	Growth Rate ~ Intercept + DMI15d + GMP + DT.h + PREC + T.sd15d + GDD15d + NDVI15d	2.964	0.017	0.596	0.81
18	Growth Rate ~ Intercept + GMP + DT.h + PREC.k15d + T.k15d + T.sd15d + T.night + GDD15d + NDVI15d	2.999	0.017	0.612	0.81
19	Growth Rate ~ Intercept + DMI15d + GMP + DT.h + PREC + T.k15d + T.sd15d + GDD15d + NDVI15d	3.052	0.016	0.628	0.81
20	Growth Rate ~ Intercept + DMI15d + GMP + DT.h + PREC.k15d + T.sd15d + T.night + GDD15d + NDVI15d	3.062	0.016	0.644	0.81
21	Growth Rate ~ Intercept + GMP + DT.h + PREC + T.k15d + T.sd15d + T.night + GDD15d + NDVI15d	3.174	0.015	0.658	0.81
22	Growth Rate ~ Intercept + DMI15d + GMP + DT.h + PREC.k15d + T.k15d + T.sd15d + T.night + GDD15d + NDVI15d	3.361	0.014	0.672	0.81
23	Growth Rate ~ Intercept + GMP + DT.h + GDD15d + NDVI15d	3.451	0.013	0.684	0.81
24	Growth Rate ~ Intercept + DMI15d + GMP + DT.h + PREC + T.sd15d + T.night + GDD15d + NDVI15d	3.730	0.012	0.696	0.81
25	Growth Rate ~ Intercept + GMP + DT.h + PREC + PREC.k15d + T.sd15d + GDD15d + NDVI15d	3.838	0.011	0.706	0.81
26	Growth Rate ~ Intercept + GMP + DT.h + PREC + PREC.k15d + T.k15d + T.sd15d + GDD15d + NDVI15d	3.842	0.011	0.717	0.81
27	Growth Rate ~ Intercept + DMI15d + GMP + DT.h + PREC + T.k15d + T.sd15d + T.night + GDD15d + NDVI15d	4.048	0.010	0.726	0.81
28	Growth Rate ~ Intercept + DMI15d + GMP + DT.h + PREC + PREC.k15d + T.sd15d + GDD15d + NDVI15d	4.218	0.009	0.735	0.81
29	Growth Rate ~ Intercept + DMI15d + GMP + DT.h + PREC + PREC.k15d + T.k15d + T.sd15d + GDD15d + NDVI15d	4.320	0.009	0.743	0.81
30	Growth Rate ~ Intercept + GMP + DT.h + T.night + GDD15d + NDVI15d	4.645	0.007	0.750	0.81
31	Growth Rate ~ Intercept + GMP + DT.h + PREC.k15d + GDD15d + NDVI15d	4.659	0.007	0.757	0.81
32	Growth Rate ~ Intercept + DMI15d + GMP + DT.h + T.k15d + T.sd15d + GDD15d	4.699	0.007	0.764	0.81
33	Growth Rate ~ Intercept + GMP + DT.h + T.k15d + GDD15d + NDVI15d	4.750	0.007	0.771	0.81
34	Growth Rate ~ Intercept + GMP + DT.h + PREC + PREC.k15d + T.sd15d + T.night + GDD15d + NDVI15d	4.814	0.007	0.777	0.81
35	Growth Rate ~ Intercept + GMP + DT.h + T.k15d + T.sd15d + GDD15d	5.036	0.006	0.783	0.81
36	Growth Rate ~ Intercept + GMP + DT.h + PREC + PREC.k15d + T.k15d + T.sd15d + T.night + GDD15d + NDVI15d	5.043	0.006	0.789	0.81
37	Growth Rate ~ Intercept + GMP + DT.h + T.sd15d + NDVI15d	5.206	0.006	0.794	0.81
38	Growth Rate ~ Intercept + DMI15d + GMP + DT.h + PREC + PREC.k15d + T.sd15d + T.night + GDD15d + NDVI15d	5.240	0.006	0.799	0.81

2014

Rank	Model	ΔAIC$_c$	wAIC$_c$	Cumulative wAIC$_c$	R_{LR}^2
39	Growth Rate ~ Intercept + GMP + DT.h + PREC + GDD15d + NDVI15d	5.302	0.005	0.804	0.81
40	Growth Rate ~ Intercept + DMI15d + GMP + DT.h + T.sd15d + GDD15d	5.329	0.005	0.809	0.81
41	Growth Rate ~ Intercept + GMP + DT.h + T.k15d + T.sd15d + NDVI15d	5.545	0.005	0.814	0.81
42	Growth Rate ~ Intercept + DMI15d + GMP + DT.h + GDD15d + NDVI15d	5.551	0.005	0.818	0.81
43	Growth Rate ~ Intercept + DMI15d + GMP + DT.h + PREC + PREC.k15d + T.k15d + T.sd15d + T.night + GDD15d + NDVI15d	5.556	0.005	0.823	0.81
44	Growth Rate ~ Intercept + GMP + DT.h + T.sd15d + GDD15d	5.741	0.004	0.827	0.81
45	Growth Rate ~ Intercept + GMP + DT.h + GDD15d	5.871	0.004	0.831	0.80
46	Growth Rate ~ Intercept + DMI15d + GMP + DT.h + T.sd15d + NDVI15d	5.875	0.004	0.835	0.81
47	Growth Rate ~ Intercept + GMP + DT.h + PREC.k15d + T.k15d + GDD15d + NDVI15d	5.949	0.004	0.838	0.81
48	Growth Rate ~ Intercept + GMP + DT.h + PREC.k15d + T.night + GDD15d + NDVI15d	6.082	0.004	0.842	0.81
49	Growth Rate ~ Intercept + GMP + DT.h + T.k15d + T.night + GDD15d + NDVI15d	6.110	0.004	0.845	0.81
50	Growth Rate ~ Intercept + DMI15d + GMP + DT.h + T.k15d + T.sd15d + T.night + GDD15d	6.129	0.004	0.848	0.81
51	Growth Rate ~ Intercept + GMP + DT.h + NDVI15d	6.140	0.004	0.852	0.80
52	Growth Rate ~ Intercept + DMI15d + GMP + DT.h + T.k15d + T.sd15d + NDVI15d	6.201	0.003	0.855	0.81
53	Growth Rate ~ Intercept + GMP + DT.h + T.k15d + GDD15d	6.444	0.003	0.858	0.81
54	Growth Rate ~ Intercept + DMI15d + GMP + DT.h + PREC.k15d + T.k15d + T.sd15d + GDD15d	6.501	0.003	0.861	0.81
55	Growth Rate ~ Intercept + DMI15d + GMP + DT.h + T.sd15d + T.night + GDD15d	6.530	0.003	0.863	0.81
56	Growth Rate ~ Intercept + DMI15d + GMP + DT.h + PREC.k15d + GDD15d + NDVI15d	6.571	0.003	0.866	0.81
57	Growth Rate ~ Intercept + DMI15d + GMP + DT.h + PREC + T.k15d + T.sd15d + GDD15d	6.596	0.003	0.869	0.81
58	Growth Rate ~ Intercept + GMP + DT.h + T.k15d + T.sd15d + T.night + GDD15d	6.606	0.003	0.871	0.81
59	Growth Rate ~ Intercept + GMP + DT.h + PREC + T.night + GDD15d + NDVI15d	6.614	0.003	0.874	0.81
60	Growth Rate ~ Intercept + GMP + DT.h + PREC + T.k15d + GDD15d + NDVI15d	6.640	0.003	0.877	0.81
61	Growth Rate ~ Intercept + GMP + DT.h + PREC + PREC.k15d + GDD15d + NDVI15d	6.687	0.003	0.879	0.81
62	Growth Rate ~ Intercept + DMI15d + GMP + DT.h + T.night + GDD15d + NDVI15d	6.749	0.003	0.882	0.81
63	Growth Rate ~ Intercept + GMP + DT.h + PREC + T.sd15d + NDVI15d	6.870	0.002	0.884	0.81
64	Growth Rate ~ Intercept + DMI15d + GMP + DT.h + T.k15d + GDD15d + NDVI15d	6.882	0.002	0.886	0.81
65	Growth Rate ~ Intercept + DMI15d + GMP + DT.h + PREC + T.sd15d + NDVI15d	6.896	0.002	0.889	0.81
66	Growth Rate ~ Intercept + DMI15d + GMP + DT.h + PREC + T.sd15d + GDD15d	7.030	0.002	0.891	0.81
67	Growth Rate ~ Intercept + GMP + DT.h + T.sd15d + T.night + GDD15d	7.096	0.002	0.893	0.81
68	Growth Rate ~ Intercept + DMI15d + GMP + DT.h + PREC.k15d + T.sd15d + GDD15d	7.129	0.002	0.895	0.81
69	Growth Rate ~ Intercept + GMP + DT.h + PREC.k15d + T.k15d + T.sd15d + GDD15d	7.170	0.002	0.897	0.81
70	Growth Rate ~ Intercept + GMP + DT.h + PREC + T.k15d + T.sd15d + GDD15d	7.183	0.002	0.899	0.81
71	Growth Rate ~ Intercept + GMP + DT.h + T.sd15d + T.night + NDVI15d	7.233	0.002	0.901	0.81
72	Growth Rate ~ Intercept + DMI15d + GMP + DT.h + PREC + GDD15d + NDVI15d	7.282	0.002	0.903	0.81
73	Growth Rate ~ Intercept + GMP + DT.h + PREC.k15d + T.sd15d + NDVI15d	7.289	0.002	0.904	0.81
74	Growth Rate ~ Intercept + GMP + DT.h + PREC + NDVI15d	7.296	0.002	0.906	0.80
75	Growth Rate ~ Intercept + DMI15d + GMP + DT.h + GDD15d	7.317	0.002	0.908	0.80
76	Growth Rate ~ Intercept + GMP + DT.h + T.night + GDD15d	7.338	0.002	0.910	0.80

2014

Rank	Model	ΔAIC$_c$	wAIC$_c$	Cumulative wAIC$_c$	R_{LR}^2
77	Growth Rate ~ Intercept + GMP + DT.h + PREC + T.k15d + T.sd15d + NDVI15d	7.346	0.002	0.912	0.81
78	Growth Rate ~ Intercept + GMP + DT.h + T.k15d + NDVI15d	7.402	0.002	0.914	0.80
79	Growth Rate ~ Intercept + DMI15d + GMP + DT.h + PREC + T.k15d + T.sd15d + NDVI15d	7.431	0.002	0.915	0.81
80	Growth Rate ~ Intercept + GMP + DT.h + PREC.k15d + T.k15d + T.night + GDD15d + NDVI15d	7.525	0.002	0.917	0.81
81	Growth Rate ~ Intercept + GMP + DT.h + T.k15d + T.sd15d + T.night + NDVI15d	7.645	0.002	0.919	0.81
82	Growth Rate ~ Intercept + GMP + DT.h + PREC.k15d + T.k15d + T.sd15d + NDVI15d	7.653	0.002	0.920	0.81
83	Growth Rate ~ Intercept + DMI15d + GMP + DT.h + PREC.k15d + T.sd15d + NDVI15d	7.711	0.002	0.922	0.81
84	Growth Rate ~ Intercept + GMP + DT.h + PREC + GDD15d	7.720	0.002	0.923	0.80
85	Growth Rate ~ Intercept + GMP + DT.h + PREC.k15d + GDD15d	7.828	0.002	0.925	0.80
86	Growth Rate ~ Intercept + GMP + DT.h + PREC + T.sd15d + GDD15d	7.836	0.002	0.926	0.81
87	Growth Rate ~ Intercept + DMI15d + GMP + DT.h + T.sd15d + T.night + NDVI15d	7.857	0.001	0.928	0.81
88	Growth Rate ~ Intercept + GMP + DT.h + PREC.k15d + T.sd15d + GDD15d	7.865	0.001	0.929	0.81
89	Growth Rate ~ Intercept + DMI15d + GMP + DT.h + PREC.k15d + T.k15d + GDD15d + NDVI15d	7.929	0.001	0.930	0.81
90	Growth Rate ~ Intercept + GMP + DT.h + PREC.k15d + NDVI15d	7.991	0.001	0.932	0.80
91	Growth Rate ~ Intercept + GMP + DT.h + PREC + PREC.k15d + T.k15d + GDD15d + NDVI15d	8.007	0.001	0.933	0.81
92	Growth Rate ~ Intercept + DMI15d + GMP + DT.h + PREC.k15d + T.night + GDD15d + NDVI15d	8.022	0.001	0.934	0.81
93	Growth Rate ~ Intercept + DMI15d + GMP + DT.h + NDVI15d	8.046	0.001	0.936	0.80
94	Growth Rate ~ Intercept + DMI15d + GMP + DT.h + PREC.k15d + T.k15d + T.sd15d + T.night + GDD15d	8.074	0.001	0.937	0.81
95	Growth Rate ~ Intercept + DMI15d + GMP + DT.h + PREC.k15d + T.k15d + T.sd15d + NDVI15d	8.077	0.001	0.938	0.81
96	Growth Rate ~ Intercept + GMP + DT.h + T.k15d + T.night + GDD15d	8.077	0.001	0.939	0.81
97	Growth Rate ~ Intercept + GMP + DT.h + PREC + T.k15d + T.night + GDD15d + NDVI15d	8.101	0.001	0.941	0.81
98	Growth Rate ~ Intercept + DMI15d + GMP + DT.h + T.k15d + GDD15d	8.109	0.001	0.942	0.81
99	Growth Rate ~ Intercept + DMI15d + GMP + DT.h + PREC + T.k15d + T.sd15d + T.night + GDD15d	8.119	0.001	0.943	0.81
100	Growth Rate ~ Intercept + GMP + DT.h + T.night + NDVI15d	8.156	0.001	0.944	0.80
101	Growth Rate ~ Intercept + GMP + DT.h + PREC + PREC.k15d + T.night + GDD15d + NDVI15d	8.171	0.001	0.945	0.81
102	Growth Rate ~ Intercept + DMI15d + GMP + DT.h + T.k15d + T.night + GDD15d + NDVI15d	8.247	0.001	0.947	0.81
103	Growth Rate ~ Intercept + DMI15d + GMP + DT.h + T.k15d + T.sd15d + T.night + NDVI15d	8.271	0.001	0.948	0.81
104	Growth Rate ~ Intercept + GMP + DT.h + PREC + T.k15d + GDD15d	8.340	0.001	0.949	0.81

2015

Rank	Model	ΔAIC_c	$wAIC_c$	Cumulative $wAIC_c$	R_{LR}^2
1	Growth Rate ~ Intercept + GMP + DT.h + PREC.k15d + T.k15d + NDVI15d	0.000	0.064	0.061	0.77
2	Growth Rate ~ Intercept + DMI15d + GMP + DT.h + PREC.k15d + T.sd15d + NDVI15d	0.522	0.049	0.107	0.77
3	Growth Rate ~ Intercept + GMP + DT.h + PREC.k15d + T.sd15d + NDVI15d	0.637	0.046	0.151	0.77
4	Growth Rate ~ Intercept + DMI15d + GMP + DT.h + PREC.k15d + T.k15d + NDVI15d	1.028	0.038	0.187	0.77
5	Growth Rate ~ Intercept + GMP + DT.h + PREC.k15d + NDVI15d	1.229	0.034	0.220	0.76
6	Growth Rate ~ Intercept + DMI15d + GMP + DT.h + PREC.k15d + T.night + NDVI15d	1.244	0.034	0.253	0.77
7	Growth Rate ~ Intercept + GMP + DT.h + PREC.k15d + T.k15d + T.sd15d + NDVI15d	1.292	0.033	0.284	0.77
8	Growth Rate ~ Intercept + GMP + DT.h + PREC.k15d + T.k15d + T.night + NDVI15d	1.366	0.032	0.315	0.77
9	Growth Rate ~ Intercept + DMI15d + GMP + DT.h + PREC.k15d + NDVI15d	1.426	0.031	0.345	0.76
10	Growth Rate ~ Intercept + GMP + DT.h + PREC.k15d + T.night + NDVI15d	1.671	0.028	0.371	0.76
11	Growth Rate ~ Intercept + DMI15d + GMP + DT.h + PREC.k15d + T.sd15d + T.night + NDVI15d	1.697	0.027	0.397	0.77
12	Growth Rate ~ Intercept + DMI15d + GMP + DT.h + PREC.k15d + T.k15d + T.sd15d + NDVI15d	1.906	0.025	0.420	0.77
13	Growth Rate ~ Intercept + DMI15d + GMP + DT.h + PREC.k15d + T.k15d + T.night + NDVI15d	1.921	0.024	0.443	0.77
14	Growth Rate ~ Intercept + GMP + DT.h + PREC + PREC.k15d + T.k15d + NDVI15d	2.061	0.023	0.465	0.77
15	Growth Rate ~ Intercept + GMP + DT.h + PREC.k15d + T.k15d + GDD15d + NDVI15d	2.095	0.022	0.486	0.77
16	Growth Rate ~ Intercept + GMP + DT.h + PREC.k15d + T.sd15d + T.night + NDVI15d	2.166	0.022	0.506	0.77
17	Growth Rate ~ Intercept + GMP + DT.h + PREC + PREC.k15d + T.sd15d + NDVI15d	2.405	0.019	0.525	0.77
18	Growth Rate ~ Intercept + GMP + DT.h + PREC.k15d + T.sd15d + GDD15d + NDVI15d	2.511	0.018	0.542	0.77
19	Growth Rate ~ Intercept + DMI15d + GMP + DT.h + PREC + PREC.k15d + T.sd15d + NDVI15d	2.561	0.018	0.559	0.77
20	Growth Rate ~ Intercept + DMI15d + GMP + DT.h + PREC.k15d + T.sd15d + GDD15d + NDVI15d	2.624	0.017	0.575	0.77
21	Growth Rate ~ Intercept + GMP + DT.h + PREC.k15d + GDD15d + NDVI15d	2.786	0.016	0.590	0.76
22	Growth Rate ~ Intercept + DMI15d + GMP + DT.h + PREC + PREC.k15d + T.night + NDVI15d	2.911	0.015	0.604	0.77
23	Growth Rate ~ Intercept + DMI15d + GMP + DT.h + PREC + PREC.k15d + T.k15d + NDVI15d	2.920	0.015	0.618	0.77
24	Growth Rate ~ Intercept + DMI15d + GMP + DT.h + PREC + PREC.k15d + NDVI15d	2.958	0.015	0.632	0.76
25	Growth Rate ~ Intercept + GMP + DT.h + PREC.k15d + T.k15d + T.sd15d + T.night + NDVI15d	3.001	0.014	0.646	0.77
26	Growth Rate ~ Intercept + DMI15d + GMP + DT.h + PREC.k15d + T.k15d + GDD15d + NDVI15d	3.059	0.014	0.659	0.77
27	Growth Rate ~ Intercept + GMP + DT.h + PREC + PREC.k15d + T.k15d + T.sd15d + NDVI15d	3.172	0.013	0.671	0.77
28	Growth Rate ~ Intercept + GMP + DT.h + PREC.k15d + T.night + GDD15d + NDVI15d	3.233	0.013	0.683	0.76
29	Growth Rate ~ Intercept + DMI15d + GMP + DT.h + PREC.k15d + T.night + GDD15d + NDVI15d	3.279	0.012	0.695	0.77
30	Growth Rate ~ Intercept + DMI15d + GMP + DT.h + PREC.k15d + T.k15d + T.sd15d + T.night + NDVI15d	3.299	0.012	0.706	0.77
31	Growth Rate ~ Intercept + GMP + DT.h + PREC + PREC.k15d + NDVI15d	3.310	0.012	0.718	0.76
32	Growth Rate ~ Intercept + GMP + DT.h + PREC + PREC.k15d + T.k15d + T.night + NDVI15d	3.383	0.012	0.729	0.77
33	Growth Rate ~ Intercept + GMP + DT.h + PREC.k15d + T.k15d + T.sd15d + GDD15d + NDVI15d	3.394	0.012	0.740	0.77
34	Growth Rate ~ Intercept + DMI15d + GMP + DT.h + PREC.k15d + GDD15d + NDVI15d	3.410	0.012	0.751	0.76
35	Growth Rate ~ Intercept + GMP + DT.h + PREC.k15d + T.k15d + T.night + GDD15d + NDVI15d	3.459	0.011	0.762	0.77
36	Growth Rate ~ Intercept + GMP + DT.h + PREC + PREC.k15d + T.night + NDVI15d	3.702	0.010	0.771	0.76
37	Growth Rate ~ Intercept + DMI15d + GMP + DT.h + PREC + PREC.k15d + T.sd15d + T.night + NDVI15d	3.718	0.010	0.781	0.77

2015

Rank	Model	ΔAIC_c	$wAIC_c$	Cumulative $wAIC_c$	R_{LR}^2
38	Growth Rate ~ Intercept + DMI15d + GMP + DT.h + PREC.k15d + T.sd15d + T.night + GDD15d + NDVI15d	3.805	0.010	0.790	0.77
39	Growth Rate ~ Intercept + DMI15d + GMP + DT.h + PREC + PREC.k15d + T.k15d + T.night + NDVI15d	3.827	0.009	0.799	0.77
40	Growth Rate ~ Intercept + GMP + DT.h + PREC + PREC.k15d + T.sd15d + T.night + NDVI15d	3.912	0.009	0.807	0.77
41	Growth Rate ~ Intercept + DMI15d + GMP + DT.h + PREC.k15d + T.k15d + T.sd15d + GDD15d + NDVI15d	3.937	0.009	0.816	0.77
42	Growth Rate ~ Intercept + DMI15d + GMP + DT.h + PREC + PREC.k15d + T.k15d + T.sd15d + NDVI15d	3.975	0.009	0.824	0.77
43	Growth Rate ~ Intercept + DMI15d + GMP + DT.h + PREC.k15d + T.k15d + T.night + GDD15d + NDVI15d	3.992	0.009	0.832	0.77
44	Growth Rate ~ Intercept + GMP + DT.h + PREC.k15d + T.sd15d + T.night + GDD15d + NDVI15d	4.000	0.009	0.841	0.77
45	Growth Rate ~ Intercept + GMP + DT.h + PREC + PREC.k15d + T.k15d + GDD15d + NDVI15d	4.163	0.008	0.848	0.77
46	Growth Rate ~ Intercept + GMP + DT.h + PREC + PREC.k15d + T.sd15d + GDD15d + NDVI15d	4.288	0.007	0.855	0.77
47	Growth Rate ~ Intercept + DMI15d + GMP + DT.h + PREC + PREC.k15d + T.sd15d + GDD15d + NDVI15d	4.668	0.006	0.861	0.77
48	Growth Rate ~ Intercept + GMP + DT.h + PREC.k15d + T.k15d	4.685	0.006	0.867	0.76
49	Growth Rate ~ Intercept + GMP + DT.h + PREC + PREC.k15d + T.k15d + T.sd15d + T.night + NDVI15d	4.863	0.006	0.872	0.77
50	Growth Rate ~ Intercept + GMP + DT.h + PREC + PREC.k15d + GDD15d + NDVI15d	4.869	0.006	0.878	0.76
51	Growth Rate ~ Intercept + DMI15d + GMP + DT.h + PREC + PREC.k15d + T.k15d + GDD15d + NDVI15d	4.901	0.005	0.883	0.77
52	Growth Rate ~ Intercept + DMI15d + GMP + DT.h + PREC + PREC.k15d + T.night + GDD15d + NDVI15d	5.009	0.005	0.888	0.77
53	Growth Rate ~ Intercept + DMI15d + GMP + DT.h + PREC + PREC.k15d + GDD15d + NDVI15d	5.035	0.005	0.893	0.76
54	Growth Rate ~ Intercept + GMP + DT.h + PREC.k15d + T.k15d + T.sd15d + T.night + GDD15d + NDVI15d	5.101	0.005	0.897	0.77
55	Growth Rate ~ Intercept + GMP + DT.h + PREC + PREC.k15d + T.night + GDD15d + NDVI15d	5.257	0.005	0.902	0.76
56	Growth Rate ~ Intercept + GMP + DT.h + PREC + PREC.k15d + T.k15d + T.sd15d + GDD15d + NDVI15d	5.280	0.005	0.906	0.77
57	Growth Rate ~ Intercept + DMI15d + GMP + DT.h + PREC + PREC.k15d + T.k15d + T.sd15d + T.night + NDVI15d	5.353	0.004	0.910	0.77
58	Growth Rate ~ Intercept + DMI15d + GMP + DT.h + PREC.k15d + T.k15d + T.sd15d + T.night + GDD15d + NDVI15d	5.366	0.004	0.914	0.77
59	Growth Rate ~ Intercept + GMP + DT.h + PREC + PREC.k15d + T.k15d + T.night + GDD15d + NDVI15d	5.481	0.004	0.918	0.77
60	Growth Rate ~ Intercept + GMP + DT.h + PREC.k15d + T.k15d + T.night	5.582	0.004	0.922	0.76
61	Growth Rate ~ Intercept + GMP + DT.h + PREC + PREC.k15d + T.sd15d + T.night + GDD15d + NDVI15d	5.755	0.004	0.925	0.77
62	Growth Rate ~ Intercept + DMI15d + GMP + DT.h + PREC + PREC.k15d + T.sd15d + T.night + GDD15d + NDVI15d	5.833	0.003	0.929	0.77
63	Growth Rate ~ Intercept + DMI15d + GMP + DT.h + PREC + PREC.k15d + T.k15d + T.night + GDD15d + NDVI15d	5.861	0.003	0.932	0.77
64	Growth Rate ~ Intercept + DMI15d + GMP + DT.h + PREC + PREC.k15d + T.k15d + T.sd15d + GDD15d + NDVI15d	5.986	0.003	0.935	0.77
65	Growth Rate ~ Intercept + GMP + DT.h + PREC.k15d + T.k15d + T.sd15d	6.260	0.003	0.937	0.76
66	Growth Rate ~ Intercept + GMP + DT.h + PREC.k15d + T.night	6.367	0.003	0.940	0.76
67	Growth Rate ~ Intercept + GMP + DT.h + PREC.k15d + T.sd15d	6.378	0.003	0.942	0.76
68	Growth Rate ~ Intercept + DMI15d + GMP + DT.h + PREC.k15d + T.k15d	6.443	0.003	0.945	0.76
69	Growth Rate ~ Intercept + GMP + DT.h + PREC.k15d + T.k15d + GDD15d	6.721	0.002	0.947	0.76
70	Growth Rate ~ Intercept + GMP + DT.h + PREC.k15d	6.755	0.002	0.949	0.76

2016

Rank	Model	ΔAIC$_c$	wAIC$_c$	Cumulative wAIC$_c$	R_{LR}^2
1	Growth Rate ~ Intercept + GMP + DT.h + PREC + T.sd15d + NDVI15d	0.000	0.048	0.045	0.80
2	Growth Rate ~ Intercept + GMP + DT.h + PREC + PREC.k15d + T.sd15d + NDVI15d	0.227	0.042	0.085	0.80
3	Growth Rate ~ Intercept + GMP + DT.h + PREC + PREC.k15d + T.sd15d + GDD15d + NDVI15d	0.297	0.041	0.124	0.80
4	Growth Rate ~ Intercept + GMP + DT.h + T.sd15d + NDVI15d	0.594	0.035	0.158	0.80
5	Growth Rate ~ Intercept + GMP + DT.h + PREC + T.sd15d + GDD15d + NDVI15d	0.612	0.035	0.191	0.80
6	Growth Rate ~ Intercept + DMI15d + GMP + DT.h + PREC + PREC.k15d + T.sd15d + NDVI15d	0.949	0.030	0.219	0.80
7	Growth Rate ~ Intercept + GMP + DT.h + T.sd15d + GDD15d + NDVI15d	0.967	0.029	0.247	0.80
8	Growth Rate ~ Intercept + GMP + DT.h + PREC + PREC.k15d + T.k15d + T.sd15d + NDVI15d	0.973	0.029	0.275	0.80
9	Growth Rate ~ Intercept + GMP + DT.h + PREC + T.k15d + T.sd15d + NDVI15d	1.318	0.025	0.298	0.80
10	Growth Rate ~ Intercept + DMI15d + GMP + DT.h + PREC + T.sd15d + NDVI15d	1.568	0.022	0.319	0.80
11	Growth Rate ~ Intercept + GMP + DT.h + T.k15d + T.sd15d + NDVI15d	1.602	0.021	0.339	0.80
12	Growth Rate ~ Intercept + GMP + DT.h + PREC.k15d + T.sd15d + GDD15d + NDVI15d	1.646	0.021	0.359	0.80
13	Growth Rate ~ Intercept + GMP + DT.h + PREC + T.sd15d + T.night + NDVI15d	1.686	0.020	0.378	0.80
14	Growth Rate ~ Intercept + GMP + DT.h + T.sd15d + T.night + NDVI15d	1.703	0.020	0.398	0.80
15	Growth Rate ~ Intercept + GMP + DT.h + PREC.k15d + T.sd15d + NDVI15d	1.724	0.020	0.417	0.80
16	Growth Rate ~ Intercept + GMP + DT.h + PREC + PREC.k15d + T.k15d + T.sd15d + GDD15d + NDVI15d	1.776	0.020	0.435	0.80
17	Growth Rate ~ Intercept + GMP + DT.h + PREC + PREC.k15d + T.sd15d + T.night + NDVI15d	1.854	0.019	0.453	0.80
18	Growth Rate ~ Intercept + DMI15d + GMP + DT.h + T.sd15d + NDVI15d	1.996	0.018	0.470	0.80
19	Growth Rate ~ Intercept + DMI15d + GMP + DT.h + PREC + PREC.k15d + T.sd15d + GDD15d + NDVI15d	2.122	0.016	0.486	0.80
20	Growth Rate ~ Intercept + DMI15d + GMP + DT.h + PREC + PREC.k15d + T.k15d + T.sd15d + NDVI15d	2.132	0.016	0.501	0.80
21	Growth Rate ~ Intercept + GMP + DT.h + PREC.k15d + T.k15d + T.sd15d + NDVI15d	2.199	0.016	0.516	0.80
22	Growth Rate ~ Intercept + GMP + DT.h + PREC + PREC.k15d + T.sd15d + T.night + GDD15d + NDVI15d	2.369	0.015	0.530	0.80
23	Growth Rate ~ Intercept + GMP + DT.h + PREC + T.k15d + T.sd15d + GDD15d + NDVI15d	2.418	0.014	0.543	0.80
24	Growth Rate ~ Intercept + GMP + DT.h + PREC + PREC.k15d + T.k15d + T.sd15d + T.night + NDVI15d	2.425	0.014	0.557	0.80
25	Growth Rate ~ Intercept + DMI15d + GMP + DT.h + PREC.k15d + T.sd15d + NDVI15d	2.440	0.014	0.570	0.80
26	Growth Rate ~ Intercept + GMP + DT.h + T.k15d + T.sd15d + T.night + NDVI15d	2.561	0.013	0.583	0.80
27	Growth Rate ~ Intercept + GMP + DT.h + T.k15d + T.sd15d + GDD15d + NDVI15d	2.607	0.013	0.595	0.80
28	Growth Rate ~ Intercept + GMP + DT.h + PREC.k15d + T.sd15d + T.night + NDVI15d	2.666	0.013	0.607	0.80
29	Growth Rate ~ Intercept + GMP + DT.h + PREC + T.sd15d + T.night + GDD15d + NDVI15d	2.668	0.013	0.619	0.80
30	Growth Rate ~ Intercept + DMI15d + GMP + DT.h + PREC + T.sd15d + GDD15d + NDVI15d	2.691	0.012	0.631	0.80
31	Growth Rate ~ Intercept + DMI15d + GMP + DT.h + PREC + PREC.k15d + T.sd15d + T.night + NDVI15d	2.734	0.012	0.642	0.80
32	Growth Rate ~ Intercept + GMP + DT.h + T.sd15d + T.night + GDD15d + NDVI15d	2.774	0.012	0.653	0.80
33	Growth Rate ~ Intercept + GMP + DT.h + PREC.k15d + T.k15d + T.sd15d + T.night + NDVI15d	2.881	0.011	0.664	0.80
34	Growth Rate ~ Intercept + GMP + DT.h + PREC + T.k15d + T.sd15d + T.night + NDVI15d	2.894	0.011	0.675	0.80
35	Growth Rate ~ Intercept + GMP + DT.h + PREC.k15d + T.k15d + T.sd15d + GDD15d + NDVI15d	2.953	0.011	0.685	0.80
36	Growth Rate ~ Intercept + DMI15d + GMP + DT.h + T.sd15d + GDD15d + NDVI15d	3.023	0.010	0.695	0.80
37	Growth Rate ~ Intercept + DMI15d + GMP + DT.h + PREC + T.k15d + T.sd15d + NDVI15d	3.129	0.010	0.704	0.80
38	Growth Rate ~ Intercept + DMI15d + GMP + DT.h + T.sd15d + T.night + NDVI15d	3.285	0.009	0.713	0.80

2016

Rank	Model	ΔAIC_c	$wAIC_c$	Cumulative $wAIC_c$	R_{LR}^2
39	Growth Rate ~ Intercept + DMI15d + GMP + DT.h + T.k15d + T.sd15d + NDVI15d	3.336	0.009	0.722	0.80
40	Growth Rate ~ Intercept + DMI15d + GMP + DT.h + PREC + T.sd15d + T.night + NDVI15d	3.350	0.009	0.730	0.80
41	Growth Rate ~ Intercept + DMI15d + GMP + DT.h + PREC.k15d + T.k15d + T.sd15d + NDVI15d	3.391	0.009	0.738	0.80
42	Growth Rate ~ Intercept + GMP + DT.h + PREC.k15d + T.sd15d + T.night + GDD15d + NDVI15d	3.425	0.009	0.747	0.80
43	Growth Rate ~ Intercept + DMI15d + GMP + DT.h + PREC.k15d + T.sd15d + GDD15d + NDVI15d	3.492	0.008	0.754	0.80
44	Growth Rate ~ Intercept + DMI15d + GMP + DT.h + PREC.k15d + T.sd15d + T.night + NDVI15d	3.621	0.008	0.762	0.80
45	Growth Rate ~ Intercept + DMI15d + GMP + DT.h + PREC + PREC.k15d + T.k15d + T.sd15d + GDD15d + NDVI15d	3.649	0.008	0.769	0.80
46	Growth Rate ~ Intercept + GMP + DT.h + PREC + PREC.k15d + T.k15d + T.sd15d + T.night + GDD15d + NDVI15d	3.757	0.007	0.776	0.80
47	Growth Rate ~ Intercept + DMI15d + GMP + DT.h + PREC + PREC.k15d + T.k15d + T.sd15d + T.night + NDVI15d	3.766	0.007	0.783	0.80
48	Growth Rate ~ Intercept + GMP + DT.h + PREC + PREC.k15d + T.sd15d + GDD15d	3.779	0.007	0.790	0.80
49	Growth Rate ~ Intercept + GMP + DT.h + PREC + T.sd15d + GDD15d	3.887	0.007	0.796	0.80
50	Growth Rate ~ Intercept + GMP + DT.h + T.sd15d + GDD15d	4.055	0.006	0.802	0.80
51	Growth Rate ~ Intercept + GMP + DT.h + PREC + T.sd15d	4.080	0.006	0.808	0.80
52	Growth Rate ~ Intercept + DMI15d + GMP + DT.h + PREC + PREC.k15d + T.sd15d + T.night + GDD15d + NDVI15d	4.181	0.006	0.814	0.80
53	Growth Rate ~ Intercept + GMP + DT.h + T.k15d + T.sd15d + T.night + GDD15d + NDVI15d	4.228	0.006	0.819	0.80
54	Growth Rate ~ Intercept + DMI15d + GMP + DT.h + PREC.k15d + T.k15d + T.sd15d + T.night + NDVI15d	4.320	0.005	0.824	0.80
55	Growth Rate ~ Intercept + GMP + DT.h + PREC + T.k15d + T.sd15d + T.night + GDD15d + NDVI15d	4.412	0.005	0.829	0.80
56	Growth Rate ~ Intercept + GMP + DT.h + PREC.k15d + T.k15d + T.sd15d + T.night + GDD15d + NDVI15d	4.451	0.005	0.834	0.80
57	Growth Rate ~ Intercept + DMI15d + GMP + DT.h + T.k15d + T.sd15d + T.night + NDVI15d	4.451	0.005	0.839	0.80
58	Growth Rate ~ Intercept + GMP + DT.h + T.sd15d	4.504	0.005	0.844	0.80
59	Growth Rate ~ Intercept + DMI15d + GMP + DT.h + PREC + T.k15d + T.sd15d + GDD15d + NDVI15d	4.510	0.005	0.849	0.80
60	Growth Rate ~ Intercept + GMP + DT.h + PREC + PREC.k15d + T.sd15d	4.600	0.005	0.853	0.80
61	Growth Rate ~ Intercept + DMI15d + GMP + DT.h + T.k15d + T.sd15d + GDD15d + NDVI15d	4.686	0.005	0.857	0.80
62	Growth Rate ~ Intercept + DMI15d + GMP + DT.h + PREC + T.sd15d + T.night + GDD15d + NDVI15d	4.748	0.004	0.862	0.80
63	Growth Rate ~ Intercept + DMI15d + GMP + DT.h + PREC + T.k15d + T.sd15d + T.night + NDVI15d	4.801	0.004	0.866	0.80
64	Growth Rate ~ Intercept + DMI15d + GMP + DT.h + T.sd15d + T.night + GDD15d + NDVI15d	4.816	0.004	0.870	0.80
65	Growth Rate ~ Intercept + DMI15d + GMP + DT.h + PREC.k15d + T.k15d + T.sd15d + GDD15d + NDVI15d	4.849	0.004	0.874	0.80
66	Growth Rate ~ Intercept + GMP + DT.h + PREC.k15d + T.sd15d + GDD15d	4.850	0.004	0.878	0.80
67	Growth Rate ~ Intercept + GMP + DT.h + PREC + PREC.k15d + T.k15d + T.sd15d	4.883	0.004	0.882	0.80
68	Growth Rate ~ Intercept + GMP + DT.h + PREC + T.k15d + T.sd15d	5.004	0.004	0.885	0.80
69	Growth Rate ~ Intercept + DMI15d + GMP + DT.h + PREC + PREC.k15d + T.sd15d	5.005	0.004	0.889	0.80
70	Growth Rate ~ Intercept + GMP + DT.h + PREC + PREC.k15d + T.k15d + T.sd15d + GDD15d	5.076	0.004	0.893	0.80
71	Growth Rate ~ Intercept + GMP + DT.h + T.k15d + T.sd15d	5.084	0.004	0.896	0.80
72	Growth Rate ~ Intercept + DMI15d + GMP + DT.h + PREC.k15d + T.sd15d + T.night + GDD15d + NDVI15d	5.210	0.004	0.900	0.80
73	Growth Rate ~ Intercept + DMI15d + GMP + DT.h + PREC + T.sd15d	5.403	0.003	0.903	0.80
74	Growth Rate ~ Intercept + GMP + DT.h + T.k15d + T.sd15d + GDD15d	5.540	0.003	0.905	0.80
75	Growth Rate ~ Intercept + GMP + DT.h + PREC + T.k15d + T.sd15d + GDD15d	5.553	0.003	0.908	0.80
76	Growth Rate ~ Intercept + DMI15d + GMP + DT.h + PREC + PREC.k15d + T.k15d + T.sd15d + T.night + GDD15d + NDVI15d	5.599	0.003	0.911	0.80

2016

Rank	Model	ΔAIC$_c$	wAIC$_c$	Cumulative wAIC$_c$	R_{LR}^2
77	Growth Rate ~ Intercept + DMI15d + GMP + DT.h + PREC + PREC.k15d + T.sd15d + GDD15d	5.628	0.003	0.914	0.80
78	Growth Rate ~ Intercept + GMP + DT.h + T.sd15d + T.night	5.643	0.003	0.916	0.80
79	Growth Rate ~ Intercept + DMI15d + GMP + DT.h + T.sd15d	5.646	0.003	0.919	0.80
80	Growth Rate ~ Intercept + GMP + DT.h + PREC + T.sd15d + T.night	5.769	0.003	0.922	0.80
81	Growth Rate ~ Intercept + GMP + DT.h + PREC.k15d + T.sd15d	5.804	0.003	0.924	0.80
82	Growth Rate ~ Intercept + GMP + DT.h + PREC.k15d + T.k15d + T.sd15d	5.810	0.003	0.926	0.80
83	Growth Rate ~ Intercept + DMI15d + GMP + DT.h + PREC + PREC.k15d + T.k15d + T.sd15d	5.851	0.003	0.929	0.80
84	Growth Rate ~ Intercept + GMP + DT.h + PREC + PREC.k15d + T.sd15d + T.night + GDD15d	5.867	0.003	0.931	0.80
85	Growth Rate ~ Intercept + DMI15d + GMP + DT.h + PREC + T.sd15d + GDD15d	5.963	0.002	0.934	0.80
86	Growth Rate ~ Intercept + GMP + DT.h + PREC + T.sd15d + T.night + GDD15d	5.968	0.002	0.936	0.80
87	Growth Rate ~ Intercept + GMP + DT.h + PREC.k15d + T.k15d + T.sd15d + GDD15d	5.971	0.002	0.938	0.80
88	Growth Rate ~ Intercept + GMP + DT.h + T.sd15d + T.night + GDD15d	5.986	0.002	0.940	0.80
89	Growth Rate ~ Intercept + GMP + DT.h + T.k15d + T.sd15d + T.night	6.053	0.002	0.943	0.80
90	Growth Rate ~ Intercept + DMI15d + GMP + DT.h + T.sd15d + GDD15d	6.110	0.002	0.945	0.80
91	Growth Rate ~ Intercept + DMI15d + GMP + DT.h + PREC.k15d + T.sd15d	6.218	0.002	0.947	0.80
92	Growth Rate ~ Intercept + GMP + DT.h + PREC + PREC.k15d + T.sd15d + T.night	6.235	0.002	0.949	0.80

2017

Rank	Model	ΔAIC$_c$	wAIC$_c$	Cumulative wAIC$_c$	R_{LR}^2
1	Growth Rate ~ Intercept + GMP + DT.h + T.night	0.000	0.024	0.023	0.77
2	Growth Rate ~ Intercept + GMP + DT.h	0.362	0.020	0.042	0.77
3	Growth Rate ~ Intercept + GMP + DT.h + T.night + NDVI15d	0.457	0.019	0.060	0.77
4	Growth Rate ~ Intercept + GMP + DT.h + NDVI15d	0.661	0.017	0.076	0.77
5	Growth Rate ~ Intercept + DMI15d + GMP + DT.h + NDVI15d	0.704	0.017	0.092	0.77
6	Growth Rate ~ Intercept + GMP + DT.h + GDD15d	0.726	0.017	0.108	0.77
7	Growth Rate ~ Intercept + DMI15d + GMP + DT.h + T.night	0.732	0.017	0.124	0.77
8	Growth Rate ~ Intercept + GMP + DT.h + T.night + GDD15d	0.780	0.016	0.139	0.77
9	Growth Rate ~ Intercept + DMI15d + GMP + DT.h + T.night + NDVI15d	0.801	0.016	0.154	0.77
10	Growth Rate ~ Intercept + DMI15d + GMP + DT.h	0.837	0.016	0.169	0.77
11	Growth Rate ~ Intercept + GMP + DT.h + GDD15d + NDVI15d	0.910	0.015	0.184	0.77
12	Growth Rate ~ Intercept + DMI15d + GMP + DT.h + T.sd15d + NDVI15d	1.011	0.014	0.198	0.77
13	Growth Rate ~ Intercept + GMP + DT.h + T.night + GDD15d + NDVI15d	1.135	0.014	0.211	0.77
14	Growth Rate ~ Intercept + DMI15d + GMP + DT.h + T.sd15d	1.578	0.011	0.221	0.77
15	Growth Rate ~ Intercept + GMP + DT.h + T.sd15d	1.666	0.010	0.231	0.77
16	Growth Rate ~ Intercept + DMI15d + GMP + DT.h + PREC.k15d + T.night	1.682	0.010	0.241	0.77
17	Growth Rate ~ Intercept + GMP + DT.h + PREC.k15d + T.night	1.725	0.010	0.250	0.77
18	Growth Rate ~ Intercept + GMP + DT.h + T.sd15d + NDVI15d	1.742	0.010	0.260	0.77
19	Growth Rate ~ Intercept + DMI15d + GMP + DT.h + PREC.k15d	1.803	0.010	0.269	0.77
20	Growth Rate ~ Intercept + DMI15d + GMP + DT.h + GDD15d + NDVI15d	1.869	0.009	0.278	0.77
21	Growth Rate ~ Intercept + GMP + DT.h + PREC	1.871	0.009	0.287	0.77
22	Growth Rate ~ Intercept + GMP + DT.h + T.k15d + T.night	1.878	0.009	0.296	0.77
23	Growth Rate ~ Intercept + GMP + DT.h + T.sd15d + T.night	1.882	0.009	0.305	0.77
24	Growth Rate ~ Intercept + DMI15d + GMP + DT.h + PREC.k15d + NDVI15d	1.905	0.009	0.313	0.77
25	Growth Rate ~ Intercept + DMI15d + GMP + DT.h + PREC.k15d + T.night + NDVI15d	1.979	0.009	0.322	0.77
26	Growth Rate ~ Intercept + DMI15d + GMP + DT.h + GDD15d	1.992	0.009	0.330	0.77
27	Growth Rate ~ Intercept + GMP + DT.h + PREC + T.night	2.005	0.009	0.339	0.77
28	Growth Rate ~ Intercept + DMI15d + GMP + DT.h + T.sd15d + T.night + NDVI15d	2.016	0.009	0.347	0.77
29	Growth Rate ~ Intercept + GMP + DT.h + PREC + NDVI15d	2.067	0.009	0.355	0.77
30	Growth Rate ~ Intercept + DMI15d + GMP + DT.h + T.night + GDD15d	2.135	0.008	0.363	0.77
31	Growth Rate ~ Intercept + GMP + DT.h + PREC.k15d	2.136	0.008	0.371	0.77
32	Growth Rate ~ Intercept + GMP + DT.h + PREC.k15d + GDD15d	2.201	0.008	0.378	0.77
33	Growth Rate ~ Intercept + GMP + DT.h + T.sd15d + T.night + NDVI15d	2.204	0.008	0.386	0.77
34	Growth Rate ~ Intercept + DMI15d + GMP + DT.h + T.night + GDD15d + NDVI15d	2.208	0.008	0.393	0.77

2017

Rank	Model	ΔAIC$_c$	wAIC$_c$	Cumulative wAIC$_c$	R_{LR}^2
35	Growth Rate ~ Intercept + GMP + DT.h + PREC.k15d + T.night + GDD15d	2.239	0.008	0.401	0.77
36	Growth Rate ~ Intercept + DMI15d + GMP + DT.h + T.sd15d + T.night	2.276	0.008	0.408	0.77
37	Growth Rate ~ Intercept + GMP + DT.h + T.k15d	2.290	0.008	0.415	0.77
38	Growth Rate ~ Intercept + GMP + DT.h + PREC + GDD15d	2.298	0.008	0.422	0.77
39	Growth Rate ~ Intercept + DMI15d + GMP + DT.h + PREC.k15d + T.sd15d + NDVI15d	2.320	0.008	0.430	0.77
40	Growth Rate ~ Intercept + GMP + DT.h + PREC.k15d + T.night + NDVI15d	2.340	0.007	0.437	0.77
41	Growth Rate ~ Intercept + GMP + DT.h + T.k15d + T.night + NDVI15d	2.365	0.007	0.444	0.77
42	Growth Rate ~ Intercept + GMP + DT.h + PREC + GDD15d + NDVI15d	2.382	0.007	0.451	0.77
43	Growth Rate ~ Intercept + GMP + DT.h + PREC + T.night + NDVI15d	2.412	0.007	0.457	0.77
44	Growth Rate ~ Intercept + DMI15d + GMP + DT.h + T.k15d + T.night	2.420	0.007	0.464	0.77
45	Growth Rate ~ Intercept + DMI15d + GMP + DT.h + PREC + NDVI15d	2.423	0.007	0.471	0.77
46	Growth Rate ~ Intercept + DMI15d + GMP + DT.h + T.k15d + NDVI15d	2.459	0.007	0.478	0.77
47	Growth Rate ~ Intercept + DMI15d + GMP + DT.h + T.k15d + T.night + NDVI15d	2.508	0.007	0.484	0.77
48	Growth Rate ~ Intercept + GMP + DT.h + T.sd15d + GDD15d	2.559	0.007	0.490	0.77
49	Growth Rate ~ Intercept + DMI15d + GMP + DT.h + T.k15d	2.574	0.007	0.497	0.77
50	Growth Rate ~ Intercept + GMP + DT.h + PREC.k15d + NDVI15d	2.585	0.007	0.503	0.77
51	Growth Rate ~ Intercept + GMP + DT.h + PREC.k15d + GDD15d + NDVI15d	2.592	0.007	0.509	0.77
52	Growth Rate ~ Intercept + DMI15d + GMP + DT.h + PREC	2.603	0.007	0.515	0.77
53	Growth Rate ~ Intercept + GMP + DT.h + T.k15d + NDVI15d	2.616	0.006	0.521	0.77
54	Growth Rate ~ Intercept + GMP + DT.h + T.sd15d + GDD15d + NDVI15d	2.623	0.006	0.528	0.77
55	Growth Rate ~ Intercept + DMI15d + GMP + DT.h + PREC.k15d + T.sd15d	2.631	0.006	0.534	0.77
56	Growth Rate ~ Intercept + GMP + DT.h + T.k15d + GDD15d	2.706	0.006	0.540	0.77
57	Growth Rate ~ Intercept + GMP + DT.h + T.k15d + T.night + GDD15d	2.717	0.006	0.545	0.77
58	Growth Rate ~ Intercept + GMP + DT.h + PREC + T.night + GDD15d	2.789	0.006	0.551	0.77
59	Growth Rate ~ Intercept + GMP + DT.h + PREC.k15d + T.night + GDD15d + NDVI15d	2.798	0.006	0.557	0.77
60	Growth Rate ~ Intercept + DMI15d + GMP + DT.h + PREC + T.night	2.809	0.006	0.562	0.77
61	Growth Rate ~ Intercept + DMI15d + GMP + DT.h + PREC.k15d + GDD15d	2.819	0.006	0.568	0.77
62	Growth Rate ~ Intercept + GMP + DT.h + T.sd15d + T.night + GDD15d	2.852	0.006	0.573	0.77
63	Growth Rate ~ Intercept + DMI15d + GMP + DT.h + PREC + T.night + NDVI15d	2.865	0.006	0.579	0.77
64	Growth Rate ~ Intercept + DMI15d + GMP + DT.h + T.sd15d + GDD15d + NDVI15d	2.891	0.006	0.584	0.77
65	Growth Rate ~ Intercept + DMI15d + GMP + DT.h + T.k15d + T.sd15d + NDVI15d	2.895	0.006	0.589	0.77
66	Growth Rate ~ Intercept + GMP + DT.h + T.k15d + GDD15d + NDVI15d	2.912	0.006	0.595	0.77
67	Growth Rate ~ Intercept + DMI15d + GMP + DT.h + PREC + T.sd15d + NDVI15d	2.922	0.006	0.600	0.77
68	Growth Rate ~ Intercept + DMI15d + GMP + DT.h + PREC.k15d + GDD15d + NDVI15d	2.934	0.006	0.605	0.77

2017

Rank	Model	ΔAIC$_c$	wAIC$_c$	Cumulative wAIC$_c$	R_{LR}^2
69	Growth Rate ~ Intercept + DMI15d + GMP + DT.h + PREC.k15d + T.night + GDD15d	2.971	0.005	0.610	0.77
70	Growth Rate ~ Intercept + GMP + DT.h + PREC + T.night + GDD15d + NDVI15d	3.091	0.005	0.615	0.77
71	Growth Rate ~ Intercept + GMP + DT.h + T.k15d + T.night + GDD15d + NDVI15d	3.100	0.005	0.620	0.77
72	Growth Rate ~ Intercept + GMP + DT.h + T.sd15d + T.night + GDD15d + NDVI15d	3.164	0.005	0.625	0.77
73	Growth Rate ~ Intercept + DMI15d + GMP + DT.h + PREC.k15d + T.night + GDD15d + NDVI15d	3.272	0.005	0.629	0.77
74	Growth Rate ~ Intercept + DMI15d + GMP + DT.h + PREC.k15d + T.sd15d + T.night + NDVI15d	3.282	0.005	0.634	0.77
75	Growth Rate ~ Intercept + DMI15d + GMP + DT.h + PREC.k15d + T.sd15d + T.night	3.290	0.005	0.638	0.77
76	Growth Rate ~ Intercept + GMP + DT.h + PREC + T.sd15d + NDVI15d	3.304	0.005	0.642	0.77
77	Growth Rate ~ Intercept + GMP + DT.h + PREC + T.sd15d	3.306	0.005	0.647	0.77
78	Growth Rate ~ Intercept + DMI15d + GMP + DT.h + T.sd15d + GDD15d	3.367	0.004	0.651	0.77
79	Growth Rate ~ Intercept + DMI15d + GMP + DT.h + PREC.k15d + T.k15d + T.night	3.368	0.004	0.655	0.77
80	Growth Rate ~ Intercept + DMI15d + GMP + DT.h + T.k15d + T.sd15d	3.432	0.004	0.659	0.77
81	Growth Rate ~ Intercept + DMI15d + GMP + DT.h + PREC + T.sd15d	3.503	0.004	0.663	0.77
82	Growth Rate ~ Intercept + GMP + DT.h + PREC.k15d + T.sd15d	3.531	0.004	0.667	0.77
83	Growth Rate ~ Intercept + DMI15d + GMP + DT.h + PREC.k15d + T.k15d	3.539	0.004	0.671	0.77
84	Growth Rate ~ Intercept + DMI15d + GMP + DT.h + PREC + PREC.k15d	3.551	0.004	0.675	0.77
85	Growth Rate ~ Intercept + DMI15d + GMP + DT.h + PREC + GDD15d + NDVI15d	3.569	0.004	0.679	0.77
86	Growth Rate ~ Intercept + GMP + DT.h + PREC + PREC.k15d	3.586	0.004	0.682	0.77
87	Growth Rate ~ Intercept + DMI15d + GMP + DT.h + PREC + PREC.k15d + NDVI15d	3.611	0.004	0.686	0.77
88	Growth Rate ~ Intercept + GMP + DT.h + PREC.k15d + T.k15d + T.night	3.633	0.004	0.690	0.77
89	Growth Rate ~ Intercept + DMI15d + GMP + DT.h + PREC.k15d + T.k15d + NDVI15d	3.659	0.004	0.694	0.77
90	Growth Rate ~ Intercept + GMP + DT.h + PREC.k15d + T.sd15d + T.night	3.666	0.004	0.697	0.77
91	Growth Rate ~ Intercept + GMP + DT.h + T.k15d + T.sd15d	3.669	0.004	0.701	0.77
92	Growth Rate ~ Intercept + DMI15d + GMP + DT.h + PREC.k15d + T.k15d + T.night + NDVI15d	3.685	0.004	0.704	0.77
93	Growth Rate ~ Intercept + GMP + DT.h + PREC + PREC.k15d + GDD15d	3.694	0.004	0.708	0.77
94	Growth Rate ~ Intercept + GMP + DT.h + PREC + PREC.k15d + T.night	3.711	0.004	0.712	0.77
95	Growth Rate ~ Intercept + DMI15d + GMP + DT.h + T.k15d + GDD15d + NDVI15d	3.733	0.004	0.715	0.77
96	Growth Rate ~ Intercept + DMI15d + GMP + DT.h + PREC + GDD15d	3.742	0.004	0.719	0.77
97	Growth Rate ~ Intercept + GMP + DT.h + PREC.k15d + T.sd15d + NDVI15d	3.745	0.004	0.722	0.77
98	Growth Rate ~ Intercept + DMI15d + GMP + DT.h + PREC + PREC.k15d + T.night	3.761	0.004	0.726	0.77
99	Growth Rate ~ Intercept + GMP + DT.h + T.k15d + T.sd15d + NDVI15d	3.774	0.004	0.729	0.77
100	Growth Rate ~ Intercept + GMP + DT.h + PREC + T.k15d	3.811	0.004	0.732	0.77
101	Growth Rate ~ Intercept + GMP + DT.h + T.k15d + T.sd15d + T.night	3.815	0.004	0.736	0.77
102	Growth Rate ~ Intercept + DMI15d + GMP + DT.h + T.k15d + T.sd15d + T.night + NDVI15d	3.839	0.004	0.739	0.77

2017

Rank	Model	ΔAIC$_c$	wAIC$_c$	Cumulative wAIC$_c$	R_{LR}^2
103	Growth Rate ~ Intercept + DMI15d + GMP + DT.h + T.k15d + GDD15d	3.843	0.004	0.742	0.77
104	Growth Rate ~ Intercept + DMI15d + GMP + DT.h + T.sd15d + T.night + GDD15d + NDVI15d	3.863	0.003	0.746	0.77
105	Growth Rate ~ Intercept + GMP + DT.h + PREC + T.k15d + T.night	3.895	0.003	0.749	0.77
106	Growth Rate ~ Intercept + GMP + DT.h + PREC + T.sd15d + T.night	3.897	0.003	0.752	0.77
107	Growth Rate ~ Intercept + DMI15d + GMP + DT.h + T.k15d + T.night + GDD15d	3.933	0.003	0.755	0.77
108	Growth Rate ~ Intercept + GMP + DT.h + PREC + PREC.k15d + NDVI15d	3.949	0.003	0.759	0.77
109	Growth Rate ~ Intercept + GMP + DT.h + PREC + PREC.k15d + GDD15d + NDVI15d	3.999	0.003	0.762	0.77
110	Growth Rate ~ Intercept + DMI15d + GMP + DT.h + T.k15d + T.night + GDD15d + NDVI15d	4.021	0.003	0.765	0.77
111	Growth Rate ~ Intercept + DMI15d + GMP + DT.h + T.sd15d + T.night + GDD15d	4.023	0.003	0.768	0.77
112	Growth Rate ~ Intercept + GMP + DT.h + PREC + T.k15d + NDVI15d	4.033	0.003	0.771	0.77
113	Growth Rate ~ Intercept + DMI15d + GMP + DT.h + PREC + PREC.k15d + T.night + NDVI15d	4.044	0.003	0.774	0.77
114	Growth Rate ~ Intercept + DMI15d + GMP + DT.h + T.k15d + T.sd15d + T.night	4.059	0.003	0.777	0.77
115	Growth Rate ~ Intercept + GMP + DT.h + PREC.k15d + T.k15d	4.090	0.003	0.780	0.77
116	Growth Rate ~ Intercept + DMI15d + GMP + DT.h + PREC + T.sd15d + T.night + NDVI15d	4.099	0.003	0.783	0.77
117	Growth Rate ~ Intercept + DMI15d + GMP + DT.h + PREC.k15d + T.sd15d + GDD15d + NDVI15d	4.114	0.003	0.786	0.77
118	Growth Rate ~ Intercept + GMP + DT.h + PREC.k15d + T.sd15d + GDD15d	4.146	0.003	0.788	0.77
119	Growth Rate ~ Intercept + GMP + DT.h + PREC.k15d + T.sd15d + T.night + NDVI15d	4.152	0.003	0.791	0.77
120	Growth Rate ~ Intercept + GMP + DT.h + PREC + T.sd15d + T.night + NDVI15d	4.170	0.003	0.794	0.77
121	Growth Rate ~ Intercept + GMP + DT.h + T.k15d + T.sd15d + T.night + NDVI15d	4.178	0.003	0.797	0.77
122	Growth Rate ~ Intercept + GMP + DT.h + PREC + T.sd15d + GDD15d + NDVI15d	4.186	0.003	0.800	0.77
123	Growth Rate ~ Intercept + DMI15d + GMP + DT.h + PREC.k15d + T.k15d + T.sd15d + NDVI15d	4.200	0.003	0.803	0.77
124	Growth Rate ~ Intercept + GMP + DT.h + PREC + T.sd15d + GDD15d	4.202	0.003	0.805	0.77
125	Growth Rate ~ Intercept + DMI15d + GMP + DT.h + PREC + T.night + GDD15d	4.207	0.003	0.808	0.77
126	Growth Rate ~ Intercept + DMI15d + GMP + DT.h + PREC + T.k15d + NDVI15d	4.209	0.003	0.811	0.77
127	Growth Rate ~ Intercept + GMP + DT.h + PREC.k15d + T.k15d + GDD15d	4.211	0.003	0.814	0.77
128	Growth Rate ~ Intercept + GMP + DT.h + PREC.k15d + T.k15d + T.night + GDD15d	4.213	0.003	0.816	0.77
129	Growth Rate ~ Intercept + DMI15d + GMP + DT.h + PREC + PREC.k15d + T.sd15d + NDVI15d	4.218	0.003	0.819	0.77
130	Growth Rate ~ Intercept + GMP + DT.h + PREC + PREC.k15d + T.night + GDD15d	4.219	0.003	0.822	0.77
131	Growth Rate ~ Intercept + DMI15d + GMP + DT.h + PREC + T.night + GDD15d + NDVI15d	4.261	0.003	0.825	0.77
132	Growth Rate ~ Intercept + GMP + DT.h + PREC.k15d + T.k15d + T.night + NDVI15d	4.271	0.003	0.827	0.77
133	Growth Rate ~ Intercept + GMP + DT.h + PREC + PREC.k15d + T.night + NDVI15d	4.280	0.003	0.830	0.77
134	Growth Rate ~ Intercept + GMP + DT.h + PREC + T.k15d + GDD15d	4.287	0.003	0.833	0.77
135	Growth Rate ~ Intercept + DMI15d + GMP + DT.h + PREC.k15d + T.sd15d + GDD15d	4.319	0.003	0.835	0.77
136	Growth Rate ~ Intercept + GMP + DT.h + PREC + T.k15d + T.night + NDVI15d	4.333	0.003	0.838	0.77

2017

Rank	Model	ΔAIC$_c$	wAIC$_c$	Cumulative wAIC$_c$	R_{LR}^2
137	Growth Rate ~ Intercept + GMP + DT.h + PREC.k15d + T.sd15d + T.night + GDD15d	4.335	0.003	0.841	0.77
138	Growth Rate ~ Intercept + DMI15d + GMP + DT.h + PREC + T.sd15d + T.night	4.366	0.003	0.843	0.77
139	Growth Rate ~ Intercept + DMI15d + GMP + DT.h + PREC + T.k15d	4.371	0.003	0.846	0.77
140	Growth Rate ~ Intercept + GMP + DT.h + PREC + T.k15d + GDD15d + NDVI15d	4.394	0.003	0.848	0.77
141	Growth Rate ~ Intercept + GMP + DT.h + PREC.k15d + T.sd15d + GDD15d + NDVI15d	4.426	0.003	0.851	0.77
142	Growth Rate ~ Intercept + DMI15d + GMP + DT.h + PREC.k15d + T.k15d + T.sd15d	4.480	0.003	0.853	0.77
143	Growth Rate ~ Intercept + DMI15d + GMP + DT.h + PREC + T.k15d + T.night	4.510	0.003	0.855	0.77
144	Growth Rate ~ Intercept + DMI15d + GMP + DT.h + PREC + PREC.k15d + T.sd15d	4.539	0.002	0.858	0.77
145	Growth Rate ~ Intercept + DMI15d + GMP + DT.h + PREC + PREC.k15d + GDD15d	4.546	0.002	0.860	0.77
146	Growth Rate ~ Intercept + GMP + DT.h + PREC.k15d + T.k15d + NDVI15d	4.559	0.002	0.862	0.77
147	Growth Rate ~ Intercept + GMP + DT.h + T.k15d + T.sd15d + GDD15d	4.577	0.002	0.865	0.77
148	Growth Rate ~ Intercept + DMI15d + GMP + DT.h + PREC + T.k15d + T.night + NDVI15d	4.589	0.002	0.867	0.77
149	Growth Rate ~ Intercept + DMI15d + GMP + DT.h + PREC + PREC.k15d + GDD15d + NDVI15d	4.615	0.002	0.869	0.77
150	Growth Rate ~ Intercept + GMP + DT.h + PREC.k15d + T.k15d + GDD15d + NDVI15d	4.619	0.002	0.872	0.77
151	Growth Rate ~ Intercept + GMP + DT.h + T.k15d + T.sd15d + GDD15d + NDVI15d	4.667	0.002	0.874	0.77
152	Growth Rate ~ Intercept + DMI15d + GMP + DT.h + PREC.k15d + T.k15d + GDD15d	4.674	0.002	0.876	0.77
153	Growth Rate ~ Intercept + GMP + DT.h + PREC + PREC.k15d + T.night + GDD15d + NDVI15d	4.726	0.002	0.878	0.77
154	Growth Rate ~ Intercept + GMP + DT.h + PREC + T.k15d + T.night + GDD15d	4.738	0.002	0.880	0.77
155	Growth Rate ~ Intercept + DMI15d + GMP + DT.h + PREC + T.sd15d + GDD15d + NDVI15d	4.772	0.002	0.882	0.77
156	Growth Rate ~ Intercept + DMI15d + GMP + DT.h + PREC.k15d + T.k15d + T.night + GDD15d	4.773	0.002	0.884	0.77
157	Growth Rate ~ Intercept + GMP + DT.h + PREC.k15d + T.k15d + T.night + GDD15d + NDVI15d	4.793	0.002	0.887	0.77
158	Growth Rate ~ Intercept + DMI15d + GMP + DT.h + PREC.k15d + T.k15d + GDD15d + NDVI15d	4.802	0.002	0.889	0.77
159	Growth Rate ~ Intercept + GMP + DT.h + T.k15d + T.sd15d + T.night + GDD15d	4.807	0.002	0.891	0.77
160	Growth Rate ~ Intercept + DMI15d + GMP + DT.h + T.k15d + T.sd15d + GDD15d + NDVI15d	4.811	0.002	0.893	0.77
161	Growth Rate ~ Intercept + DMI15d + GMP + DT.h + PREC + T.k15d + T.sd15d + NDVI15d	4.822	0.002	0.895	0.77
162	Growth Rate ~ Intercept + GMP + DT.h + PREC + T.sd15d + T.night + GDD15d	4.870	0.002	0.897	0.77
163	Growth Rate ~ Intercept + GMP + DT.h + PREC.k15d + T.sd15d + T.night + GDD15d + NDVI15d	4.881	0.002	0.899	0.77
164	Growth Rate ~ Intercept + DMI15d + GMP + DT.h + PREC.k15d + T.sd15d + T.night + GDD15d	4.929	0.002	0.901	0.77
165	Growth Rate ~ Intercept + DMI15d + GMP + DT.h + PREC.k15d + T.sd15d + T.night + GDD15d + NDVI15d	5.035	0.002	0.903	0.77
166	Growth Rate ~ Intercept + DMI15d + GMP + DT.h + PREC + PREC.k15d + T.night + GDD15d	5.042	0.002	0.904	0.77
167	Growth Rate ~ Intercept + DMI15d + GMP + DT.h + PREC.k15d + T.k15d + T.sd15d + T.night	5.066	0.002	0.906	0.77
168	Growth Rate ~ Intercept + GMP + DT.h + PREC + T.k15d + T.night + GDD15d + NDVI15d	5.069	0.002	0.908	0.77
169	Growth Rate ~ Intercept + DMI15d + GMP + DT.h + PREC.k15d + T.k15d + T.night + GDD15d + NDVI15d	5.091	0.002	0.910	0.77
170	Growth Rate ~ Intercept + DMI15d + GMP + DT.h + PREC.k15d + T.k15d + T.sd15d + T.night + NDVI15d	5.099	0.002	0.912	0.77

2017

Rank	Model	ΔAIC$_c$	wAIC$_c$	Cumulative wAIC$_c$	R_{LR}^2
171	Growth Rate ~ Intercept + GMP + DT.h + PREC + PREC.k15d + T.sd15d	5.120	0.002	0.913	0.77
172	Growth Rate ~ Intercept + GMP + DT.h + PREC + T.sd15d + T.night + GDD15d + NDVI15d	5.130	0.002	0.915	0.77
173	Growth Rate ~ Intercept + GMP + DT.h + T.k15d + T.sd15d + T.night + GDD15d + NDVI15d	5.158	0.002	0.917	0.77
174	Growth Rate ~ Intercept + DMI15d + GMP + DT.h + PREC + T.sd15d + GDD15d	5.259	0.002	0.918	0.77
175	Growth Rate ~ Intercept + DMI15d + GMP + DT.h + T.k15d + T.sd15d + GDD15d	5.267	0.002	0.920	0.77
176	Growth Rate ~ Intercept + GMP + DT.h + PREC + PREC.k15d + T.sd15d + NDVI15d	5.275	0.002	0.922	0.77
177	Growth Rate ~ Intercept + GMP + DT.h + PREC + T.k15d + T.sd15d	5.315	0.002	0.923	0.77
178	Growth Rate ~ Intercept + DMI15d + GMP + DT.h + PREC + PREC.k15d + T.k15d	5.318	0.002	0.925	0.77
179	Growth Rate ~ Intercept + DMI15d + GMP + DT.h + PREC + PREC.k15d + T.night + GDD15d + NDVI15d	5.323	0.002	0.926	0.77
180	Growth Rate ~ Intercept + GMP + DT.h + PREC + T.k15d + T.sd15d + NDVI15d	5.341	0.002	0.928	0.77
181	Growth Rate ~ Intercept + DMI15d + GMP + DT.h + PREC + PREC.k15d + T.sd15d + T.night + NDVI15d	5.368	0.002	0.930	0.77
182	Growth Rate ~ Intercept + DMI15d + GMP + DT.h + PREC + T.k15d + T.sd15d	5.374	0.002	0.931	0.77
183	Growth Rate ~ Intercept + DMI15d + GMP + DT.h + PREC + PREC.k15d + T.sd15d + T.night	5.383	0.002	0.933	0.77
184	Growth Rate ~ Intercept + DMI15d + GMP + DT.h + PREC + PREC.k15d + T.k15d + NDVI15d	5.396	0.002	0.934	0.77
185	Growth Rate ~ Intercept + DMI15d + GMP + DT.h + PREC + T.k15d + GDD15d + NDVI15d	5.461	0.002	0.936	0.77
186	Growth Rate ~ Intercept + DMI15d + GMP + DT.h + PREC + PREC.k15d + T.k15d + T.night	5.461	0.002	0.937	0.77
187	Growth Rate ~ Intercept + GMP + DT.h + PREC.k15d + T.k15d + T.sd15d	5.550	0.001	0.939	0.77
188	Growth Rate ~ Intercept + GMP + DT.h + PREC + PREC.k15d + T.k15d	5.553	0.001	0.940	0.77
189	Growth Rate ~ Intercept + GMP + DT.h + PREC.k15d + T.k15d + T.sd15d + T.night	5.619	0.001	0.941	0.77
190	Growth Rate ~ Intercept + DMI15d + GMP + DT.h + PREC + T.k15d + GDD15d	5.621	0.001	0.943	0.77
191	Growth Rate ~ Intercept + GMP + DT.h + PREC + PREC.k15d + T.k15d + T.night	5.633	0.001	0.944	0.77
192	Growth Rate ~ Intercept + GMP + DT.h + PREC + PREC.k15d + T.sd15d + T.night	5.666	0.001	0.945	0.77
193	Growth Rate ~ Intercept + GMP + DT.h + PREC + PREC.k15d + T.sd15d + GDD15d	5.704	0.001	0.947	0.77
194	Growth Rate ~ Intercept + GMP + DT.h + PREC + PREC.k15d + T.k15d + GDD15d	5.716	0.001	0.948	0.77
195	Growth Rate ~ Intercept + DMI15d + GMP + DT.h + T.k15d + T.sd15d + T.night + GDD15d + NDVI15d	5.730	0.001	0.949	0.77

2018

Rank	Model	ΔAIC$_c$	wAIC$_c$	Cumulative wAIC$_c$	R_{LR}^2
1	Growth Rate ~ Intercept + DMI15d + GMP + DT.h + PREC + T.sd15d + T.night + GDD15d	0.000	0.051	0.048	0.79
2	Growth Rate ~ Intercept + DMI15d + GMP + DT.h + T.sd15d + GDD15d	0.201	0.046	0.092	0.79
3	Growth Rate ~ Intercept + DMI15d + GMP + DT.h + PREC + PREC.k15d + T.sd15d + T.night + GDD15d	0.382	0.042	0.132	0.80
4	Growth Rate ~ Intercept + DMI15d + GMP + DT.h + GDD15d	0.542	0.039	0.168	0.79
5	Growth Rate ~ Intercept + DMI15d + GMP + DT.h + PREC + T.sd15d + GDD15d	0.887	0.033	0.199	0.79
6	Growth Rate ~ Intercept + DMI15d + GMP + DT.h + T.sd15d + T.night + GDD15d	1.286	0.027	0.225	0.79
7	Growth Rate ~ Intercept + DMI15d + GMP + DT.h + PREC.k15d + T.sd15d + GDD15d	1.326	0.026	0.250	0.79
8	Growth Rate ~ Intercept + DMI15d + GMP + DT.h + PREC + GDD15d	1.393	0.025	0.274	0.79
9	Growth Rate ~ Intercept + DMI15d + GMP + DT.h + T.sd15d + GDD15d + NDVI15d	1.632	0.022	0.295	0.79
10	Growth Rate ~ Intercept + DMI15d + GMP + DT.h + PREC + T.k15d + T.sd15d + T.night + GDD15d	1.688	0.022	0.316	0.80
11	Growth Rate ~ Intercept + DMI15d + GMP + DT.h + T.k15d + T.sd15d + GDD15d	1.694	0.022	0.336	0.79
12	Growth Rate ~ Intercept + DMI15d + GMP + DT.h + PREC.k15d + GDD15d	1.755	0.021	0.356	0.79
13	Growth Rate ~ Intercept + DMI15d + GMP + DT.h + PREC + T.sd15d + T.night + GDD15d + NDVI15d	1.782	0.021	0.376	0.80
14	Growth Rate ~ Intercept + DMI15d + GMP + DT.h + PREC + PREC.k15d + T.sd15d + GDD15d	1.808	0.021	0.396	0.79
15	Growth Rate ~ Intercept + DMI15d + GMP + DT.h + PREC + T.k15d + T.sd15d + GDD15d	1.873	0.020	0.414	0.79
16	Growth Rate ~ Intercept + DMI15d + GMP + DT.h + PREC + PREC.k15d + T.k15d + T.sd15d + T.night + GDD15d	2.066	0.018	0.432	0.80
17	Growth Rate ~ Intercept + DMI15d + GMP + DT.h + PREC + T.sd15d + GDD15d + NDVI15d	2.227	0.017	0.447	0.79
18	Growth Rate ~ Intercept + DMI15d + GMP + DT.h + PREC.k15d + T.sd15d + T.night + GDD15d	2.264	0.016	0.463	0.79
19	Growth Rate ~ Intercept + DMI15d + GMP + DT.h + GDD15d + NDVI15d	2.273	0.016	0.478	0.79
20	Growth Rate ~ Intercept + DMI15d + GMP + DT.h + PREC + PREC.k15d + T.sd15d + T.night + GDD15d + NDVI15d	2.302	0.016	0.494	0.80
21	Growth Rate ~ Intercept + DMI15d + GMP + DT.h + PREC + PREC.k15d + GDD15d	2.431	0.015	0.508	0.79
22	Growth Rate ~ Intercept + DMI15d + GMP + DT.h + T.k15d + GDD15d	2.484	0.015	0.522	0.79
23	Growth Rate ~ Intercept + GMP + DT.h + PREC + PREC.k15d + T.sd15d + T.night + GDD15d	2.553	0.014	0.535	0.79
24	Growth Rate ~ Intercept + DMI15d + GMP + DT.h + T.night + GDD15d	2.573	0.014	0.549	0.79
25	Growth Rate ~ Intercept + DMI15d + GMP + DT.h + PREC + PREC.k15d + T.k15d + T.sd15d + GDD15d	2.702	0.013	0.561	0.79
26	Growth Rate ~ Intercept + DMI15d + GMP + DT.h + PREC.k15d + T.k15d + T.sd15d + GDD15d	2.790	0.013	0.573	0.79
27	Growth Rate ~ Intercept + DMI15d + GMP + DT.h + PREC.k15d + T.sd15d + GDD15d + NDVI15d	2.884	0.012	0.585	0.79
28	Growth Rate ~ Intercept + DMI15d + GMP + DT.h + PREC + T.night + GDD15d	2.955	0.012	0.596	0.79
29	Growth Rate ~ Intercept + DMI15d + GMP + DT.h + T.sd15d + T.night + GDD15d + NDVI15d	2.978	0.011	0.606	0.79
30	Growth Rate ~ Intercept + DMI15d + GMP + DT.h + PREC + GDD15d + NDVI15d	3.072	0.011	0.617	0.79
31	Growth Rate ~ Intercept + DMI15d + GMP + DT.h + T.k15d + T.sd15d + GDD15d + NDVI15d	3.072	0.011	0.627	0.79
32	Growth Rate ~ Intercept + DMI15d + GMP + DT.h + PREC + T.k15d + GDD15d	3.110	0.011	0.637	0.79
33	Growth Rate ~ Intercept + DMI15d + GMP + DT.h + PREC + T.k15d + T.sd15d + GDD15d + NDVI15d	3.112	0.011	0.648	0.79
34	Growth Rate ~ Intercept + DMI15d + GMP + DT.h + T.k15d + T.sd15d + T.night + GDD15d	3.149	0.011	0.657	0.79
35	Growth Rate ~ Intercept + DMI15d + GMP + DT.h + PREC + PREC.k15d + T.sd15d + GDD15d + NDVI15d	3.293	0.010	0.667	0.79
36	Growth Rate ~ Intercept + GMP + DT.h + PREC + PREC.k15d + T.k15d + T.sd15d + T.night + GDD15d	3.391	0.009	0.676	0.79
37	Growth Rate ~ Intercept + DMI15d + GMP + DT.h + PREC + T.k15d + T.sd15d + T.night + GDD15d + NDVI15d	3.393	0.009	0.684	0.80
38	Growth Rate ~ Intercept + DMI15d + GMP + DT.h + PREC.k15d + GDD15d + NDVI15d	3.579	0.008	0.693	0.79

2018

Rank	Model	ΔAIC$_c$	wAIC$_c$	Cumulative wAIC$_c$	R_{LR}^2
39	Growth Rate ~ Intercept + DMI15d + GMP + DT.h + PREC.k15d + T.k15d + GDD15d	3.694	0.008	0.700	0.79
40	Growth Rate ~ Intercept + DMI15d + GMP + DT.h + PREC.k15d + T.night + GDD15d	3.769	0.008	0.707	0.79
41	Growth Rate ~ Intercept + DMI15d + GMP + DT.h + T.sd15d	3.810	0.008	0.715	0.79
42	Growth Rate ~ Intercept + DMI15d + GMP + DT.h + PREC + PREC.k15d + T.night + GDD15d	3.855	0.007	0.722	0.79
43	Growth Rate ~ Intercept + DMI15d + GMP + DT.h + PREC + PREC.k15d + T.k15d + T.sd15d + T.night + GDD15d + NDVI15d	3.929	0.007	0.728	0.80
44	Growth Rate ~ Intercept + DMI15d + GMP + DT.h + PREC.k15d + T.sd15d + T.night + GDD15d + NDVI15d	4.079	0.007	0.735	0.79
45	Growth Rate ~ Intercept + DMI15d + GMP + DT.h + PREC + PREC.k15d + T.k15d + T.sd15d + GDD15d + NDVI15d	4.100	0.007	0.741	0.80
46	Growth Rate ~ Intercept + DMI15d + GMP + DT.h + PREC + PREC.k15d + T.k15d + GDD15d	4.116	0.006	0.747	0.79
47	Growth Rate ~ Intercept + DMI15d + GMP + DT.h + PREC.k15d + T.k15d + T.sd15d + T.night + GDD15d	4.135	0.006	0.753	0.79
48	Growth Rate ~ Intercept + GMP + DT.h + PREC + PREC.k15d + T.sd15d + T.night + GDD15d + NDVI15d	4.152	0.006	0.759	0.79
49	Growth Rate ~ Intercept + DMI15d + GMP + DT.h + PREC + PREC.k15d + GDD15d + NDVI15d	4.217	0.006	0.765	0.79
50	Growth Rate ~ Intercept + DMI15d + GMP + DT.h + T.k15d + GDD15d + NDVI15d	4.218	0.006	0.771	0.79
51	Growth Rate ~ Intercept + DMI15d + GMP + DT.h + PREC.k15d + T.k15d + T.sd15d + GDD15d + NDVI15d	4.303	0.006	0.776	0.79
52	Growth Rate ~ Intercept + GMP + DT.h + PREC + T.sd15d + T.night + GDD15d	4.340	0.006	0.782	0.79
53	Growth Rate ~ Intercept + DMI15d + GMP + DT.h + T.night + GDD15d + NDVI15d	4.360	0.006	0.787	0.79
54	Growth Rate ~ Intercept + DMI15d + GMP + DT.h	4.431	0.006	0.793	0.79
55	Growth Rate ~ Intercept + DMI15d + GMP + DT.h + PREC.k15d + T.sd15d	4.512	0.005	0.798	0.79
56	Growth Rate ~ Intercept + DMI15d + GMP + DT.h + T.k15d + T.night + GDD15d	4.571	0.005	0.803	0.79
57	Growth Rate ~ Intercept + GMP + DT.h + PREC + PREC.k15d + T.k15d + T.sd15d + GDD15d	4.697	0.005	0.807	0.79
58	Growth Rate ~ Intercept + DMI15d + GMP + DT.h + T.k15d + T.sd15d + T.night + GDD15d + NDVI15d	4.772	0.005	0.812	0.79
59	Growth Rate ~ Intercept + DMI15d + GMP + DT.h + PREC + T.k15d + GDD15d + NDVI15d	4.784	0.005	0.816	0.79
60	Growth Rate ~ Intercept + DMI15d + GMP + DT.h + PREC + PREC.k15d + T.sd15d + T.night	4.795	0.005	0.820	0.79
61	Growth Rate ~ Intercept + DMI15d + GMP + DT.h + PREC + T.night + GDD15d + NDVI15d	4.816	0.005	0.825	0.79
62	Growth Rate ~ Intercept + GMP + DT.h + PREC + PREC.k15d + T.k15d + T.sd15d + T.night + GDD15d + NDVI15d	4.875	0.004	0.829	0.79
63	Growth Rate ~ Intercept + DMI15d + GMP + DT.h + PREC + T.k15d + T.night + GDD15d	4.902	0.004	0.833	0.79
64	Growth Rate ~ Intercept + DMI15d + GMP + DT.h + T.sd15d + T.night	4.914	0.004	0.837	0.79
65	Growth Rate ~ Intercept + DMI15d + GMP + DT.h + PREC + T.sd15d + T.night	4.919	0.004	0.841	0.79
66	Growth Rate ~ Intercept + GMP + DT.h + PREC + T.k15d + T.sd15d + T.night + GDD15d	4.925	0.004	0.846	0.79
67	Growth Rate ~ Intercept + DMI15d + GMP + DT.h + T.sd15d + NDVI15d	4.993	0.004	0.850	0.79
68	Growth Rate ~ Intercept + DMI15d + GMP + DT.h + PREC + T.sd15d	5.192	0.004	0.853	0.79
69	Growth Rate ~ Intercept + DMI15d + GMP + DT.h + PREC.k15d	5.259	0.004	0.857	0.79
70	Growth Rate ~ Intercept + GMP + DT.h + PREC.k15d + T.sd15d + GDD15d	5.392	0.003	0.860	0.79
71	Growth Rate ~ Intercept + GMP + DT.h + PREC + PREC.k15d + T.k15d + T.sd15d + GDD15d + NDVI15d	5.396	0.003	0.863	0.79
72	Growth Rate ~ Intercept + DMI15d + GMP + DT.h + PREC.k15d + T.sd15d + T.night	5.414	0.003	0.866	0.79
73	Growth Rate ~ Intercept + GMP + DT.h + PREC + T.sd15d + T.night + GDD15d + NDVI15d	5.472	0.003	0.869	0.79
74	Growth Rate ~ Intercept + GMP + DT.h + PREC + PREC.k15d + T.sd15d + GDD15d	5.483	0.003	0.873	0.79
75	Growth Rate ~ Intercept + DMI15d + GMP + DT.h + PREC.k15d + T.k15d + GDD15d + NDVI15d	5.523	0.003	0.876	0.79
76	Growth Rate ~ Intercept + GMP + DT.h + PREC.k15d + T.k15d + T.sd15d + GDD15d	5.577	0.003	0.879	0.79

2018

Rank	Model	ΔAIC$_c$	wAIC$_c$	Cumulative wAIC$_c$	R_{LR}^2
77	Growth Rate ~ Intercept + GMP + DT.h + PREC.k15d + T.sd15d + T.night + GDD15d	5.641	0.003	0.881	0.79
78	Growth Rate ~ Intercept + DMI15d + GMP + DT.h + PREC.k15d + T.night + GDD15d + NDVI15d	5.655	0.003	0.884	0.79
79	Growth Rate ~ Intercept + GMP + DT.h + PREC + T.k15d + T.sd15d + GDD15d	5.676	0.003	0.887	0.79
80	Growth Rate ~ Intercept + DMI15d + GMP + DT.h + PREC + PREC.k15d + T.sd15d	5.701	0.003	0.890	0.79
81	Growth Rate ~ Intercept + DMI15d + GMP + DT.h + PREC.k15d + T.k15d + T.night + GDD15d	5.777	0.003	0.893	0.79
82	Growth Rate ~ Intercept + GMP + DT.h + PREC + T.k15d + T.sd15d + GDD15d + NDVI15d	5.801	0.003	0.895	0.79
83	Growth Rate ~ Intercept + DMI15d + GMP + DT.h + T.k15d + T.sd15d	5.805	0.003	0.898	0.79
84	Growth Rate ~ Intercept + DMI15d + GMP + DT.h + PREC + PREC.k15d + T.k15d + T.night + GDD15d	5.811	0.003	0.901	0.79
85	Growth Rate ~ Intercept + DMI15d + GMP + DT.h + PREC + PREC.k15d + T.night + GDD15d + NDVI15d	5.821	0.003	0.903	0.79
86	Growth Rate ~ Intercept + DMI15d + GMP + DT.h + PREC.k15d + T.sd15d + NDVI15d	5.864	0.003	0.906	0.79
87	Growth Rate ~ Intercept + GMP + DT.h + PREC + T.k15d + T.sd15d + T.night + GDD15d + NDVI15d	5.891	0.003	0.908	0.79
88	Growth Rate ~ Intercept + DMI15d + GMP + DT.h + PREC.k15d + T.k15d + T.sd15d + T.night + GDD15d + NDVI15d	5.893	0.003	0.911	0.79
89	Growth Rate ~ Intercept + DMI15d + GMP + DT.h + PREC + PREC.k15d + T.k15d + GDD15d + NDVI15d	5.902	0.003	0.913	0.79
90	Growth Rate ~ Intercept + DMI15d + GMP + DT.h + PREC	5.966	0.003	0.916	0.79
91	Growth Rate ~ Intercept + DMI15d + GMP + DT.h + NDVI15d	6.002	0.003	0.918	0.79
92	Growth Rate ~ Intercept + GMP + DT.h + T.sd15d + GDD15d	6.183	0.002	0.920	0.79
93	Growth Rate ~ Intercept + GMP + DT.h + T.k15d + T.sd15d + GDD15d	6.191	0.002	0.923	0.79
94	Growth Rate ~ Intercept + GMP + DT.h + PREC + PREC.k15d + T.sd15d + GDD15d + NDVI15d	6.218	0.002	0.925	0.79
95	Growth Rate ~ Intercept + GMP + DT.h + PREC.k15d + T.sd15d + GDD15d + NDVI15d	6.221	0.002	0.927	0.79
96	Growth Rate ~ Intercept + DMI15d + GMP + DT.h + PREC + T.sd15d + NDVI15d	6.287	0.002	0.929	0.79
97	Growth Rate ~ Intercept + DMI15d + GMP + DT.h + T.k15d + T.night + GDD15d + NDVI15d	6.324	0.002	0.931	0.79
98	Growth Rate ~ Intercept + DMI15d + GMP + DT.h + T.sd15d + T.night + NDVI15d	6.377	0.002	0.933	0.79
99	Growth Rate ~ Intercept + GMP + DT.h + PREC.k15d + T.k15d + T.sd15d + GDD15d + NDVI15d	6.389	0.002	0.935	0.79
100	Growth Rate ~ Intercept + DMI15d + GMP + DT.h + PREC + T.sd15d + T.night + NDVI15d	6.431	0.002	0.937	0.79
101	Growth Rate ~ Intercept + DMI15d + GMP + DT.h + T.night	6.477	0.002	0.939	0.79
102	Growth Rate ~ Intercept + GMP + DT.h + T.k15d + T.sd15d + GDD15d + NDVI15d	6.486	0.002	0.941	0.79
103	Growth Rate ~ Intercept + DMI15d + GMP + DT.h + PREC.k15d + T.k15d + T.sd15d	6.488	0.002	0.942	0.79
104	Growth Rate ~ Intercept + GMP + DT.h + T.sd15d + GDD15d + NDVI15d	6.496	0.002	0.944	0.79
105	Growth Rate ~ Intercept + DMI15d + GMP + DT.h + T.k15d	6.508	0.002	0.946	0.79
106	Growth Rate ~ Intercept + DMI15d + GMP + DT.h + PREC + PREC.k15d + T.sd15d + T.night + NDVI15d	6.510	0.002	0.948	0.79
107	Growth Rate ~ Intercept + GMP + DT.h + T.sd15d + T.night + GDD15d	6.590	0.002	0.950	0.79
